# Small vertebrate consumption by capuchin monkeys (*Sapajus libidinosus*): Insights into the human predatory pattern

**DOI:** 10.1371/journal.pone.0355204

**Published:** 2026-08-26

**Authors:** Susana Carvalho, Noemi Spagnoletti, João d’Oliveira Coelho, Megan Beardmore-Herd, Olívia Mendonça-Furtado, Michele Verderane, Joana Prieto, Dominique I. Cavallaro, Patrícia Izar, Elisabetta Visalberghi

**Affiliations:** 1 Department of Science, Gorongosa National Park, Sofala, Mozambique; 2 CIBIO/InBIO, Centro de Investigação em Biodiversidade e Recursos Genéticos, Universidade do Porto, Campus de Vairão, Vairão, Portugal; 3 ICArEHB-Interdisciplinary Center for Archaeology and Evolution of Human Behaviour, Universidade do Algarve, Campus Gambelas, Faro, Portugal; 4 Department of Experimental Psychology, Institute of Psychology, University of São Paulo, São Paulo, Brazil; 5 Global Mammal Assessment Lab, Department of Biology and Biotechnology Charles Darwin, Sapienza University of Rome, Rome, Italy; 6 Istituto di Scienze e Tecnologie della Cognizione, Consiglio Nazionale delle Ricerche, Rome, Italy; 7 Primate Models for Behavioural Evolution Lab, Institute of Human Sciences, University of Oxford, Oxford, United Kingdom; 8 Department of Life Sciences, University of Coimbra, Coimbra, Portugal; 9 School of Anthropology, Pontificia Universidad Católica de Chile, Santiago, Chile; Lyon College, UNITED STATES OF AMERICA

## Abstract

The consumption of small vertebrates occurs to varying degrees among many non-human primates. This dietary element may have constituted an important source of nutrition for early hominins, particularly during periods of food scarcity, providing a stepping stone to the exploitation of larger animals. Capuchin monkeys regularly consume small vertebrates across their range. Expanding our knowledge of the contribution of animal matter to these primates’ diets can shed light on the proximate and ultimate drivers of small vertebrate consumption across primate taxa, including humans. We report on the most extensive record of bearded capuchin (*Sapajus libidinosus*) vertebrate predation so far available: 45 months of observations of vertebrate predation in two sympatric groups in Fazenda Boa Vista, in northeastern Brazil. We estimated the effect of provisioning, sex, age class, fruit and insect availability, rainfall, humidity, and temperature on predation rates using a zero-inflated negative binomial algorithm with random effects. We found mammal and saurian consumption is habitual in this population of *S. libidinosus*. Prey brains and viscera tend to be accessed before the rest of the body. Predation rates were higher in males compared to females and in adults compared to infants across groups. Our results also identified small vertebrates as key energy sources for bearded capuchins under decreased fruit availability and show that provisioning reduces predation rates. Future work should assess small vertebrate predation as a possible stepping stone in the emergence of human exploitation of large animals by re-analyzing fossil faunal assemblages for evidence of small vertebrate predation.

## Introduction

The habitual acquisition and consumption of animal matter is a shared behaviour among humans and non-human primates (hereafter primates) [1]. Despite this, humans are the only extant primate known to regularly exploit animals as large as, or larger than, themselves [2]. This strategy has been termed the “human predatory pattern” [3]. The emergence of this behaviour may have enabled the evolution of larger brains, and the adaptive suite of defining human characteristics that came with it [4–12]. Furthermore, in combination with cooperative hunting and technological advances, accessing large animal resources may have acted as a buffer to seasonality in plant food availability [3,13].

Prior to the emergence of the human predatory pattern, small vertebrates (< 5 kg) may have constituted an important source of nutrition for hominins [14]. Being easier and less costly to catch, the opportunistic exploitation of small animals could have been a crucial stepping stone facilitating the later emergence of large prey hunting. Support for this can be found in the zooarchaeological record. Faunal assemblages dating to around 2 million years ago already provide strong evidence of sustained small animal resource exploitation by hominins with limited technological repertoires [15,16]. Later on, there is also evidence of small vertebrate consumption by Holocene North and South American hunter-gatherers [17,18]. Furthermore, small game is a resource regularly accessed by several modern human hunter-gatherer populations, among them the!Kung community of Botswana [19] and the Hadza community of Tanzania [20].

Ingestion of vertebrate tissue has been reported inat least 89 species of modern primates [1]. Among the taxons where this behaviour is most frequently observed are capuchin monkeys (genus *Cebus*, or gracile capuchins, and genus *Sapajus*, or robust capuchins) [1]. Capuchins are characterized as highly opportunistic, generalist omnivores [21–26]. They are able to prey upon fast-moving invertebrates and small vertebrates, as well as extract difficult-to-access food items using advanced cognitive and complex manipulative skills [21–26]. In some capuchin populations, vertebrate consumption accounts for 2–3% of overall feeding time [25,27]. This number can even go up to 15% for some individuals during peak predation months [25,27].

The list of vertebrates that capuchin monkeys prey upon varies depending on site and species (Table 1). To date, this list includes mammals, such as squirrels (*Sciurus* sp.), coatis (*Nasua narica*), rodents (including the large rock cavie, *Kerodon rupestris*), juvenile anteaters (*Tamandua mexicana*) and bats; reptiles and amphibians, including snakes, lizards, frogs, and freshwater turtle eggs; and birds of several species [22,23,25–41]. Capuchin monkeys also capture and feed upon other primates. In Ilha de Germoplasma in eastern Brazil a tufted capuchin monkey (*Cebus apella*; *S. apella*) was seen to kill and feed upon an infant dusky titi monkey (*Plecturocebus moloch*) [42]. A group of the same capuchin species was observed feeding on the carcass of an adult owl monkey (*Aotus brumbacki*) in the Colombian Llanos [43]. Additionally, an adult male capuchin monkey (*Sapajus* sp.) in the Ibura National Forest of Brazil was reported to capture and presumably ingest a common marmoset (*Callithrix jacchus*), although predation was not observed directly [44].

**Table 1:** Vertebrates in the diet of capuchin monkeys.

Prey class	Prey type	Capuchin species	Site	Citations
Saurian *	Lizard	Bearded capuchin (*Sapajus libidinosus*)	Serra da Capivara National Park (Brazil)	[39]
		Golden-bellied capuchin (*Sapajus xanthosternos*)	Una Biological Reserve (Brazil)	[28]
		Blond capuchin (*Sapajus flavius*)	Caatinga dry forest area (Brazil)	[26]
			Reserva Particular do Patrimônio Natural Engenho Gargaú (Brazil)	[41]
		White-faced capuchin (*Cebus capucinus*)	Santa Rosa National Park (Costa Rica)	[22,39]
			Lomas Barbudal (Costa Rica)	[39]
	Snake	Bearded capuchin (*Sapajus libidinosus*)	Serra da Capivara National Park (Brazil)	[29,30]
			Fazenda Boa Vista (Brazil)	[30]
	Turtle egg	White-fronted capuchin (*Cebus albifrons*)	Jaú National Park (Brasil)	[34]
	Iguana	White-faced capuchin (*Cebus capucinus*)	Santa Rosa National Park (Costa Rica)	[39]
	Adult bird	Bearded capuchin (*Sapajus libidinosus*)	Serra da Capivara National Park (Brazil)	[29]
		White-faced capuchin (*Cebus capucinus*)	Santa Rosa National Park (Costa Rica)	[22,25,39]
		*Sapajus* spp. (Captive hybrid)	Tietê Ecological Park (Brazil)	[31]
	Bird egg/nestling	White-faced capuchin (*Cebus capucinus*)	Santa Rosa National Park (Costa Rica)	[22,25,39]
			Lomas Barbudal (Costa Rica)	[39]
			Palo Verde (Costa Rica)	[39]
		*Sapajus* spp. (Captive hybrid)	Tietê Ecological Park (Brazil)	[31]
Mammal	Rodent	Bearded capuchin (*Sapajus libidinosus*)	Serra da Capivara National Park (Brazil)	[29]
			Serra das Almas Private Reserve of Natural Heritage (Brazil)	[32]
		Azaras’s capuchin (*Sapajus cay*)	Cabeceira do Prata Private Reserve (Brazil)	[35]
		*Sapajus* spp. (Captive hybrid)	Tietê Ecological Park (Brazil)	[38]
		White-faced capuchin (*Cebus capucinus*)	Santa Rosa National Park (Costa Rica)	[25,39]
			Lomas Barbudal (Costa Rica)	[39]
	Bat	Bearded capuchin (*Sapajus libidinosus*)	Serra da Capivara National Park (Brazil)	[29]
		White-faced capuchin (*Cebus capucinus*)	Santa Rosa National Park (Costa Rica)	[22,25,39]
			Lomas Barbudal (Costa Rica)	[39]
	Squirrel	White-faced capuchin (*Cebus capucinus*)	Santa Rosa National Park (Costa Rica)	[22,25]
			Lomas Barbudal (Costa Rica)	[39]
			Palo Verde (Costa Rica)	[39]
		Black capuchin (*Sapajus nigritus*)	Santa Genebra (Brazil)	[33]
	Coati	White-faced capuchin (*Cebus capucinus*)	Santa Rosa National Park (Costa Rica)	[22,25,36,37]
			Lomas Barbudal (Costa Rica)	[39]
			Palo Verde (Costa Rica)	[39]
	Marsupial	*Sapajus* spp. (Captive hybrid)	Tietê Ecological Park (Brazil)	[38]
		Large-headed capuchin (*Sapajus macrocephalus*)	Manu Learning Centre Biological Station (Peru)	[45]
	Anteater (juvenile)	White-faced capuchin (*Cebus capucinus*)	Lomas Barbudal (Costa Rica)	[39]
	Primate	Brown or tufted capuchin (*Sapajus apella*)	Ilha de Germoplasma (Brazil)	[42]
			Arrayanes Farm (Colombia)	[43]
		*Sapajus* spp. (unidentified)	Ibura National Forest (Brazil)	[44]
Amphibian	Frog	White-faced capuchin (*Cebus capucinus*)	Santa Rosa National Park (Costa Rica)	[39]

*Clade composed of Lepidosauromorpha (snakes, lizards and rhynchocephalians) and Archosauromorpha (birds and crocodilians) [46].

The regular access to vertebrate prey of smaller or similar size to their own makes the capuchin dietary pattern an interesting model of the human predatory pattern [3], as well as the habitual small animal consumption that may have preceded its emergence [14]. In addition to commonly consuming vertebrates in the wild [22,25,29], capuchins exhibit a high degree of terrestriality [47], have complex food acquisition practices involving tool use and high levels of manual dexterity [48], and have the largest relative brain sizes of the platyrrhine primates [23]. Continuing to expand our knowledge of the contribution of animal matter to these primates’ diets allows us to shed light on the ultimate and proximate drivers of this behaviour, as well as reflect on the role of animal matter in human evolution.

Sex biases in vertebrate predation rates have been reported in some capuchin populations [22,25,29,39,49]. For example, at the long-term site of Santa Rosa National Park, Costa Rica, white-faced capuchin males attempt to and successfully catch more vertebrate prey than females [22,25,39,49]. Similar results have been found in chimpanzees [50–52], and across the primate order more broadly [1,2]. This sexual bias has been hypothesized to be linked to sexual dimorphism in body mass demanding different caloric intake demands from males compared to females [53]. At Fazenda Boa Vista (Brazil), the only field site where bearded capuchins (*S. libidinosus*) have been systematically weighed, the average body mass of adult subordinate male capuchins is 3.5 kg (range: 3.4–3.6 kg) while that of adult female capuchins is 2.1 kg (range: 1.8–2.6 kg) [54]. Thus, adult male capuchins may engage in more vertebrate consumption events than adult females to meet their greater energetic needs [49]. Furthermore, whilst reproduction and lactation increase the energetic needs of females [55], they may buffer this demand by targeting reliable, low-risk food items, such as invertebrates [21], as opposed to vertebrate prey. This is supported by reports of female capuchins consuming more invertebrates than males on average [49].

There is less evidence on the effect of seasonality on vertebrate predation rates among capuchin monkeys. White-faced capuchin monkeys at Santa Rosa (Costa Rica) show increased vertebrate predation rates towards the dry season compared to the wet season, a period which coincides with some prey species’ birthing season [22] and lower plant food abundance [25]. However, predation rates over some prey species, like birds, do not appear to follow seasonal trends [25]. In general, fruits and insects may be preferred over vertebrate prey for their higher net energy yield per unit of foraging time [22]. The exception may occur in periods where fruit or insect availability is low enough that the nutritional yield of hunting vertebrates becomes “worthwhile” [22]. Addressing this question requires testing of the effects of fruit and insect abundance over vertebrate predation rates in capuchins.

Capuchin monkeys appear to be highly socially tolerant in feeding and foraging activities [37,38,56]. Food transfer refers to an event where the original hunter does not monopolize the vertebrate prey, but rather parts/all of it are passed to others either passively or actively [31]. This behavior has been recorded in bearded [32], brown [31] and white-faced capuchins [25]. Understanding the extent to which this behaviour is ubiquitous across capuchin populations requires information from more sites in these taxa’s distribution.

Much of our understanding of capuchin monkey vertebrate consumption stems from studies of gracile capuchins [22,25,34,39]. Less focus has been given to vertebrate predation behaviour in robust capuchin monkeys. However, recent field studies are beginning to overcome this bias. In a two-year-long study in the Caatinga dry forest of Serra da Capivara National Park in Brazil, predation events by wild bearded capuchin monkeys (*Sapajus libidinosus*) were found to be relatively common with an overall rate of 4.6 events/100h [29]. Furthermore, this Caatinga population is known to use probe tools to aid predation of small vertebrates like lizards, snakes, birds and rodents, as well as larger prey, such as rock cavies (*Kerodon rupestris*) [29,57].

This study had the aim of further expanding our understanding of the contribution of animal resources to robust capuchins’ diet. To this end, we collected data on the vertebrate prey accessed by two sympatric groups of wild bearded capuchin monkeys (classified as Near Threatened by the IUCN Red List [58]) from Fazenda Boa Vista, north-eastern Brazil, (one of which was slightly provisioned daily during the dry season) across 45 months. In particular, we tested the following hypotheses:

1 Effect of provisioning on predation rates:

The provisioned group has a higher net caloric intake than the unprovisioned group, as they have increased food availability and decreased food search costs. Therefore, the unprovisioned group will have greater need for vertebrate prey as a source of calories, and their vertebrate predation rates will be higher.

2 Temporal variability in alternative food availability:
  2.1 Vertebrate prey replaces other high-protein food items that are less costly to obtain, such as insects, during periods of scarcity (e.g., during the dry season [59]) [22]. Therefore, vertebrate predation rates will increase during insect-poor periods [22].
  2.2 Vertebrate prey replaces other high-calorie food sources that are less costly to obtain, like fruits, during periods of scarcity [22]. Therefore, vertebrate predation rates will increase when fruit availability decreases [22].
3 Environmental effects over predation rates:
  3.1 Environmental factors (rainfall, humidity, and temperature) will not affect capuchin vertebrate predation rates.
  3.2 Alternatively, environmental factors (rainfall, humidity, and temperature) will have an effect on capuchin vertebrate predation rates.
4 Age biases in predation rates:
  4.1 Since the metabolism of juveniles expends more energy than the metabolism of adults, juvenile vertebrate predation rates will be higher than in adults.
  4.2 Alternatively, due to the difficulties of hunting vertebrate prey, adults, who have more hunting experience, will show higher vertebrate predation rates than juveniles and infants, who have less hunting experience.
5 Sexual biases in predation rates:
  5.1 Since different body mass among individuals of a given species demand different caloric intake [53,60], and the body mass of adult male capuchins is on average 60% larger than adult females [54], adult male capuchins will engage in more vertebrate consumption events than adult females.
  5.2 Alternatively, since greater energetic needs are placed on adult females compared to adult males by reproduction and lactation [55], adult females will engage in more vertebrate consumption events than adult males.
6 Food transfer:

Given capuchin monkeys’ social tolerance in feeding and foraging activities [37,38,56] and previous reports of food transfer events in bearded capuchins [29,32], food transfer events will be observed in the study population.

## Materials and methods

### Site

This study took place in Fazenda Boa Vista (hereafter, FBV; 9º 39’ 36” S, 45º 25’ 10” W), a privately owned area located in the northeastern Brazilian state of Piauí, 21 km northwest of the town of Gilbués. FBV is a transition zone between the Brazilian biomes *Cerrado* and *Caatinga*. It consists of a sandy plain surrounded by 20–100 meter cliffs and interspersed by sandstone ridges and plateaus [61]. The majority of the plain is characterised by a high abundance of palms and medium-height trees, whereas the lowest part of the plain is a marsh conformed of gallery forest vegetation [61]. Meanwhile, the cliffs are dominated by shrubs and small trees, and the sandstone plateaus by low herbaceous vegetation [61]. The climate is characterized by marked rainfall seasonality which influences the availability of water in the ephemeral watercourses of the region and allows the distinction of two climatic seasons: a wet season from October to April and a dry season from May to September [59]. While the annual average rainfall in this area is 1156 mm, only an average of 230 mm of rain falls during the dry season [61].

### Subjects

Our subjects belong to two wild and sympatric groups of bearded capuchin monkeys (*Sapajus libidinosus*), one of which was attracted to visit a study area by provisioning it daily with nuts, corn and water during the study period [59]. On average, each individual of the provisioned group received 825 kJ every day [59]. Both the unprovisioned group (composed by 8–16 individuals) and the provisioned group (composed by 17–19 individuals) have been continuously observed since 2005 as part of the Etho*Cebus* long-term research project on behaviour and ecology of wild bearded capuchin monkeys [62], and were thus well habituated to human presence.

### Observations

Observations were carried out between May 2006 and December 2010 (Table 2). The overall observations of the unprovisioned group accounted for 3012 hours (distributed across 43 months across wet and dry seasons) and those of the provisioned group accounted for 2812 hours (distributed across 45 months across wet and dry seasons) (Table 2). Data on vertebrate consumption events were collected following all occurrence sampling [63] and group scan sampling methods [64]. Since the sampling interval for collection of group scan activity budgets was different among observers, we divided the data collection into two study periods, S1 and S2, corresponding to the length of interval used.

**Table 2:** Observation and demographic details for the two groups of capuchin monkeys studied at Fazenda Boa Vista: dates of observation periods, number of observation hours, study group (CH: Chicão, provisioned group; ZA: Zangado, unprovisioned group), group size, sex composition (♂: Males; ♀: Females), and age class composition (A: Adults, J: Juveniles, I: Infants).

Observation period^a^	Observation hours	Study group	Group size^b^	♂	♀	A	J	I
June 2006 – May 2007^1^	706	CH	12-18	11	7	11	5	2
	349	ZA	8-14	7	7	8	4	2
May 2006 – April 2008^2^	775	CH	17-19	13	8	12	4	5
	1932	ZA	8-14	7	8	9	4	2
Jan. 2009 – Dec. 2010^3^	1331	CH	17-19	11	10	9	8	5
	733	ZA	13-16	12	7	8	6	6

^a^Data sources: ^1^(81); ^2^(82); ^3^(83)

^b^Group size differed across observation periods due to births/deaths/migration.

In S1 (May 2006 to April 2008), we recorded data on activity budgets using a 10 min group scan sampling method at 10 min intervals, for a total of 84,470 scans (provisioned group: n = 42,917; unprovisioned group: n = 41,553). In S2 (April 2009 to December 2010), we recorded activity budgets using 1 min group scan sampling at 5 min intervals for a total of 75,028 scans (provisioned group: n = 62,588; unprovisioned group: n = 12,440). To test whether differences in sampling interval between study periods (S1 and S2) affected our results, we ran a generalized linear mixed model (GLMM) with a Poisson error structure. The response variable was the number of scans per month in which vertebrate consumption was observed, with study period (S1 vs S2) included as a fixed effect and total monthly scans as a covariate. Since we did not find a significant effect of study period in this model (χ² = 1.065, df = 1, p = 0.302), we considered all data collected by scan sampling during both study periods together in further analysis.

A *Consumption Event* was defined as an individual observed taking and at least partially consuming any vertebrate item. We considered nestlings and eggs as vertebrate consumption [25]. We also scored which individual(s) captured the prey and which individual(s) ate it, and described which body parts were eaten. Hunting events were labelled Type I when one individual hunted the prey and Type II when more than one individual hunted the same prey simultaneously. Events in which food-transfer occurred were classified by referring to the individual(s) initially possessing the food item as *possessor(s)*, and the individual(s) to whom the transfer is directed as *recipient(s)* [31].

### Data analysis: Statistical modelling

We modelled the rate of predation events per unit of observation time as a function of the following fixed effect variables:

(1) Rainfall (mm). Recorded at the field station (Hygro-Thermometer Clock model 445702′, EXTECH instruments) daily.
(2) Temperature (ºC). Daily maximum and minimum temperatures were recorded at the field station (Hygro-Thermometer Clock model 445702′, EXTECH instruments).
(3) Humidity (%): Daily minimum and maximum recorded at the field station (Hygro-Thermometer Clock model 445702′, EXTECH instruments).
(4) Fruit availability. Estimated by combining:
  (a) Stratified random sampling method, whereby 100 fruit traps were placed at 30 m intervals, 1 m to the side of a 3 km trail crossing the different physiognomies of the study area. At two-week intervals, fallen material in the traps was quantified and weighed *in situ* and then returned to the same location, so fruits were not removed from their natural environment.. Food availability index (FAI; kg/ha) was estimated based on the dry weight of the material collected from traps and on the cumulative surface area.
  (b) Monthly direct observation and recording of presence/absence of fruit in 254 palm trees of the species catulé (*Attalea barreirensis*) (N = 136) and piassava (*Orbignya* sp.) (N = 118), in the same 3 km trail. This data was used to estimate two monthly FAI, one expressed as the number of palms with fruits/ha and one for total nuts, as the sum of catulé and piassava palms with fruits/ha.
(5) Insect availability. Estimated through stratified random sampling method, whereby 100 pitfall traps were placed at 30 m intervals, 1 m to the side of the same 3 km trail used to estimate fruit availability. At two-week intervals, fallen material in the traps was quantified and weighed in situ and then returned to the same location, so material was not removed from its natural environment. Food availability index (FAI; kg/ha) was estimated based on the dry weight of the material collected from traps and on the cumulative surface area [59].
(6) Sex.
(7) Age.
(8) Group (unprovisioned vs. provisioned).

Correlations among predictor variables were assessed using Spearman’s rank correlation coefficient (ρ) and variance inflation factors (VIFs) (S1 Table). Spearman’s ρ indicated only low to moderate pairwise associations, and all VIF values were below 1.25, suggesting negligible multicollinearity. The strongest association was a moderate correlation between insect availability and rainfall (ρ = 0.45). However, low VIF values indicated that the model was still able to distinguish between the seasonal increase in insect availability and the direct effect of rainfall on capuchin predation activity. In addition, model comparison based on Akaike Information Criterion (AIC) [65] values showed that models including both environmental variables (rainfall, temperature, and humidity) and food availability variables (fruit and insect availability) consistently outperformed models including environmental variables alone.

Our data set had many observations with 0 predation events documented. Since count data with many zero values cannot be transformed to normal [66], we implemented a predictive model capable of directly estimating the predation events observed. This predictive model also deals with excesses of zeros, and with non-linearity, non-homogeneity of variance and an outcome variable bounded at zero [67]. Excess zeros arise from low frequency of occurrences in behavioural studies and are thus influenced by an array of life-history processes and the ecological context. We can also have false zeros, that is when occurrences are happening, but not during the surveying period, or when occurrences happen during surveying but are not detected [68,69]. When working with longitudinal data (characterized by repeated measurements from individuals within and across units of time), it is necessary to explicitly model the random effects structure. This allows for a correct inference of the fixed effects, accounting for non-independence among observations and avoiding pseudoreplication [70,71]. Thus, we relied on zero-inflated generalized linear mixed models (ziglmm) to perform the analysis, more specifically a zero-inflated negative binomial (zinb) algorithm with crossed random effects. To standardize for observation effort, we included the log of the number of scan samples as an offset term (*logtime*), so that the model estimates predation rates per unit observation effort rather than raw counts. Therefore, our model was defined as follows:


$$\begin{array}{cc} Predation\;Events\;\sim & \;off\;set\;(logtime)\;+\;Sex\;+\;Group\;+\;Rainfall\;+\;Fruit\;+\;Insect\;+\;(\text{1}/ID)\;\\ & +\;(\text{1}/Month)\;Zero\;Inflation\;\sim \;Humidity\;+\;Temperature \end{array}$$ (1)

where the random effects of individual ID and month (1–45) are expressed in parentheses.

The negative binomial distribution is technically Poisson-distributed with underlying gamma-distributed heterogeneity [72]. So, it is better suited than a typical Poisson regression to account for overdispersion [73]. All parameters were fitted in R with the glmmTMB package, using maximum likelihood estimation, except for random effects which were estimated via a Laplace approximation [74–76]. After running many different model architectures, we used information-theory for multi-model inference to select the final reduced model. Notice that some architectures could not be modelled because of convergence problems while estimating parameters (e.g., non-positive-definite Hessian matrix; extreme or very small eigenvalues detected; and/or singular convergence). We selected as the final model (eq. 1) the one with the lowest Akaike Information Criterion (AIC) value, as this metric optimises the trade-off between fit and complexity [65,71,77].

The specified model (eq. 1) was applied to all collected data on vertebrate predation (Model 1), as well as two subsets of it: predation events involving mammal prey only (Model 2) and predation events involving non-mammal prey only (Model 3). We refer to non-mammalian prey as Sauria, a clade composed of Lepidosauromorpha (snakes, lizards and rhynchocephalians) and Archosauromorpha (birds and crocodilians) [46]. Models 2 and 3 were applied to compare patterns of mammal vs. saurian predation in the studied populations.

Additionally, we also used Non-parametric tests (Chi-square tests) to assess whether hunting events were more frequently performed by one (Type I) or more individuals (Type II) and whether consumption was more frequently performed by one or more individuals. Statistical significance level was set at *α* = 0*.*05 for all of the analyses performed.

### Ethical note

The research adhered to the Brazilian government permit (SisBio #28689) in accordance with the Brazilian legislation (law #11.794, October 8, 2008) and to the Brazilian National Council for Scientific and Technological Development (CNPq) authorization for Scientific expedition, under the Project CMC 000111, which included the permission from the landowners Marino Gomes de Oliveira and Maria Fonseca Gomes de Oliveira to conduct research in their field site. We adhered to the American Society of Primatologists’ principles for the ethical treatment of primates.

### Inclusivity in global research

Additional information regarding the ethical, cultural, and scientific considerations specific to inclusivity in global research is included in the Supporting Information (S1 Checklist).

## Results

Each group of capuchins was followed from dawn to dusk. During a total of 5,093 observation hours we obtained a total of 446 events of vertebrate consumption (both when in progress or about to begin). Of the 446 events, 281 were recorded by scan sampling and 165 were recorded by all occurrence sampling. The provisioned group accounted for 212 events, and the unprovisioned group for 234 events. We recorded 8 events of vertebrate consumption by infants (3 lizards, 2 rodents, 2 snakes, 1 undetermined prey). For the modelling analyses only the scan sampling events were used. We obtained an overall rate of 6.34 predation events/100h.

### Consumed prey items

Similarly to what has been observed elsewhere [31,35,38], the most common prey of capuchins in FBV are vertebrates of small dimensions like lizards and rodents. The major prey types were diurnal and abundant (pers. com.) reptiles (n = 136: lizards, geckos, snakes, and iguanas), mammals (n = 97: primarily rodents, but also opossums, and two bats) and birds (n = 34: especially nestlings and eggs) (Table 3; see S1 Video).

**Table 3:** Number of prey items consumed in the 281 events of vertebrate consumption recorded by scan sampling in the provisioned and unprovisioned groups. Birds include both nestling and eggs. Und. = Undetermined.

Mammals			Saurian				Und.
Rodents	Marsupials	Bats	Lizards	Birds	Snakes	Iguana	
80	15	2	123	34	12	1	14
28.5%	5.3%	0.7%	43.8%	12.1%	4.3%	0.4%	5.0%

**Fig 1:**
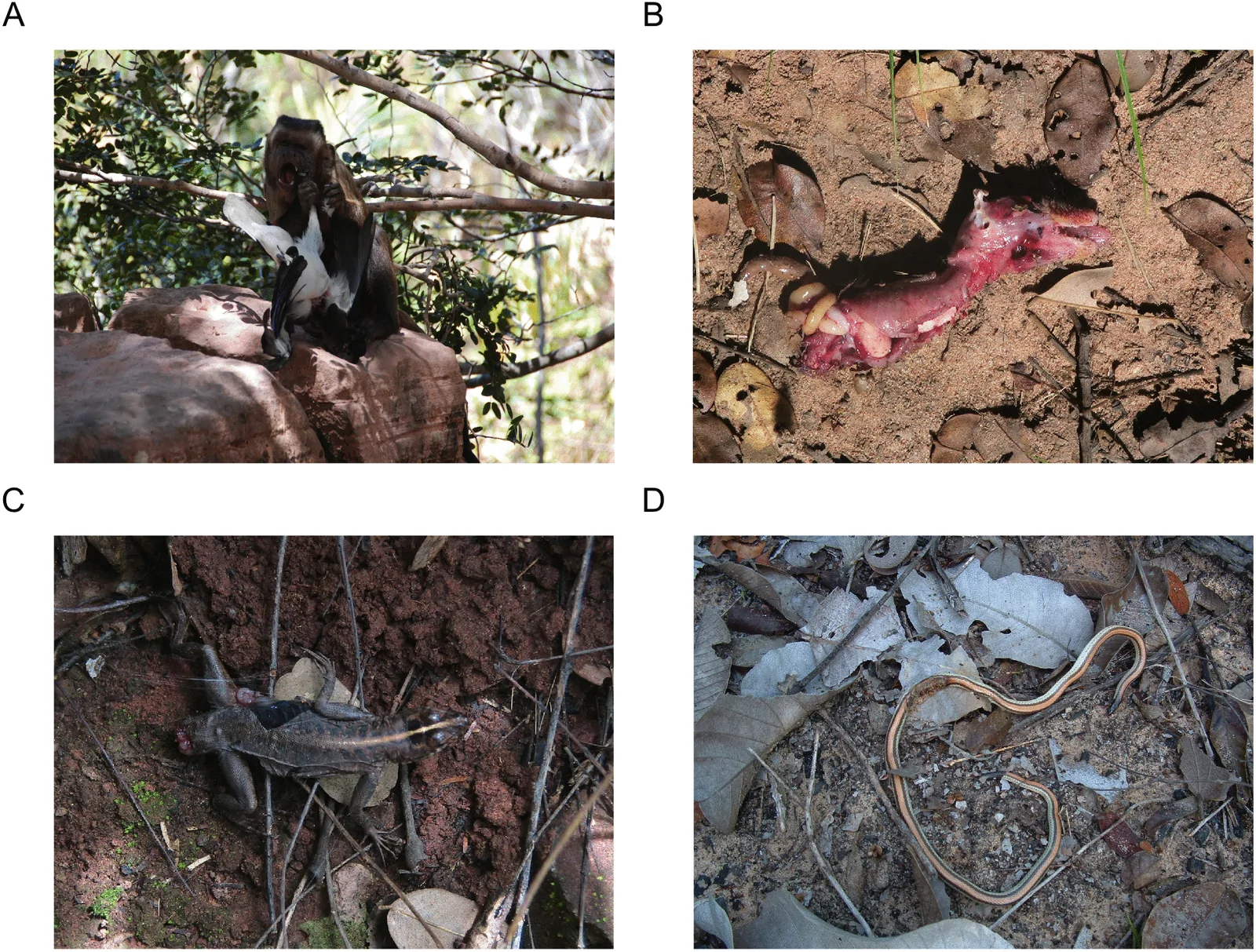
Evidence of vertebrate consumption at FBV. A (top left): Adult male feeding on avian prey (photo by Luciano Candisani); B (top right): Mammal prey (marsupial) preyed upon by an adult male. The skin was ripped off and the viscera exposed (photo by Noemi Spagnoletti); C (bottom left): Lizard preyed upon by an adult female. Viscera were exposed (photo by Eduardo D. R. Silva); D (bottom right): Snake preyed upon by an unidentified individual. The upper part of the snake containing the head was ripped off and the viscera exposed (photo by Noemi Spagnoletti).

Avian prey were usually eaten entirely (Fig 1A). When this was not the case, the monkeys preferred to eat the brain and viscera. For mammalian prey the monkeys preferred to eat the tail and paws, and in some events the eyes as well. The skin covering the forehead and the back of the prey was ripped off in a few cases (Fig 1B). For geckos and lizards, capuchins frequently ate feet, tail, eyes, brain, skin and, in some cases, viscera (Fig 1C). Only male capuchin monkeys were observed consuming snakes. When eating snakes (Fig 1D), they often discarded the head and ate the rest of the body, especially the internal organs. In general, muscular tissue and associated ligaments and tendons were torn apart in small bites, repeatedly masticated and swallowed; tender parts of the prey, such as the viscera and brain, were ingested more rapidly. We never observed meat consumption accompanied by leaf ingestion.

Due to limited numbers of observations in certain categories (e.g., 2 bats, 12 snakes, etc.), we concatenated small prey subsets, enabling statistical analyses and modelling. For example, alongside a global model, we compared mammals versus saurians using two distinct sub-models.

### Model 1: Global model for vertebrate consumption

**Fig 2:**
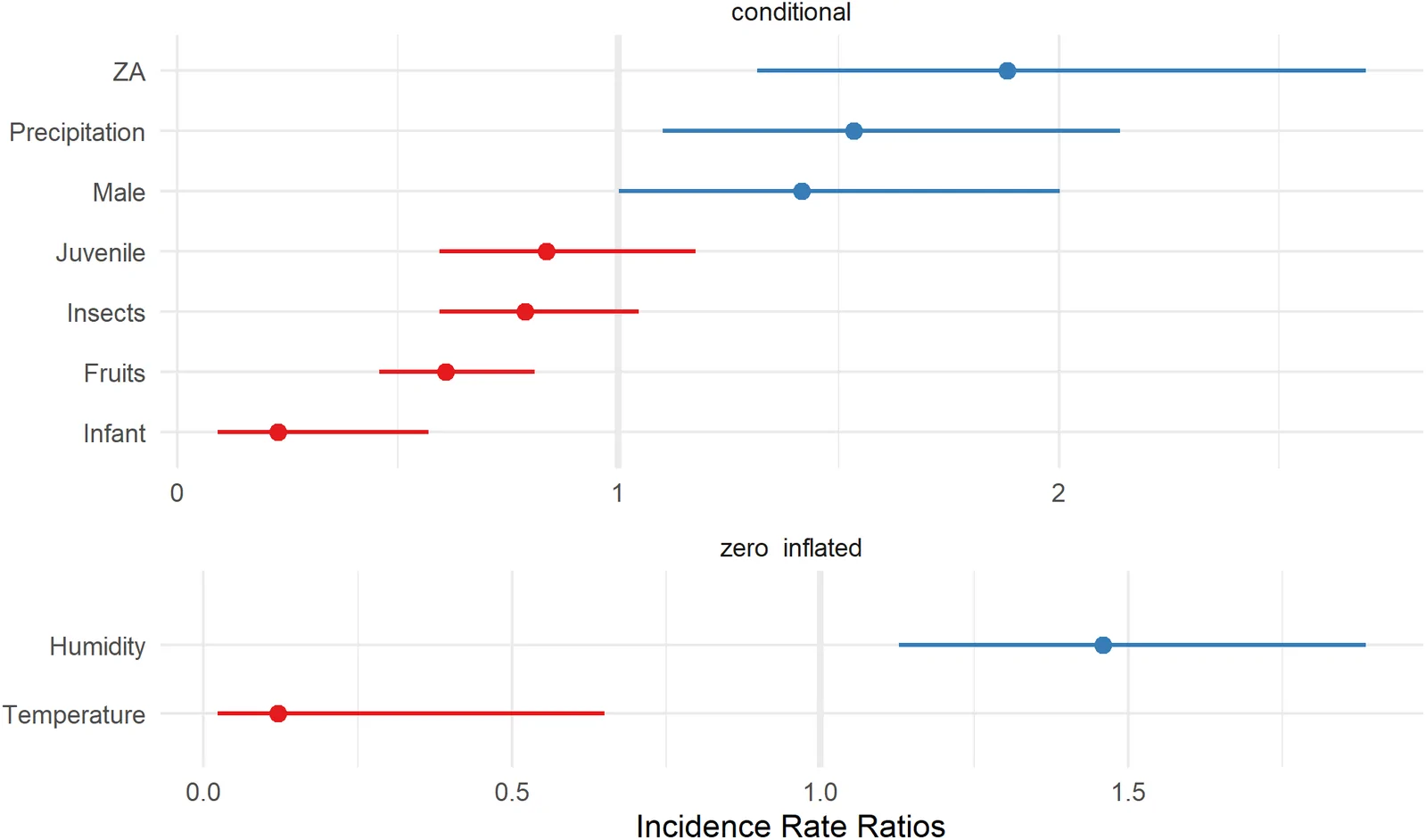
Dot-whisker plot showing the incidence rate estimates for predation events based on all variables in the model.

**Fig 3:**
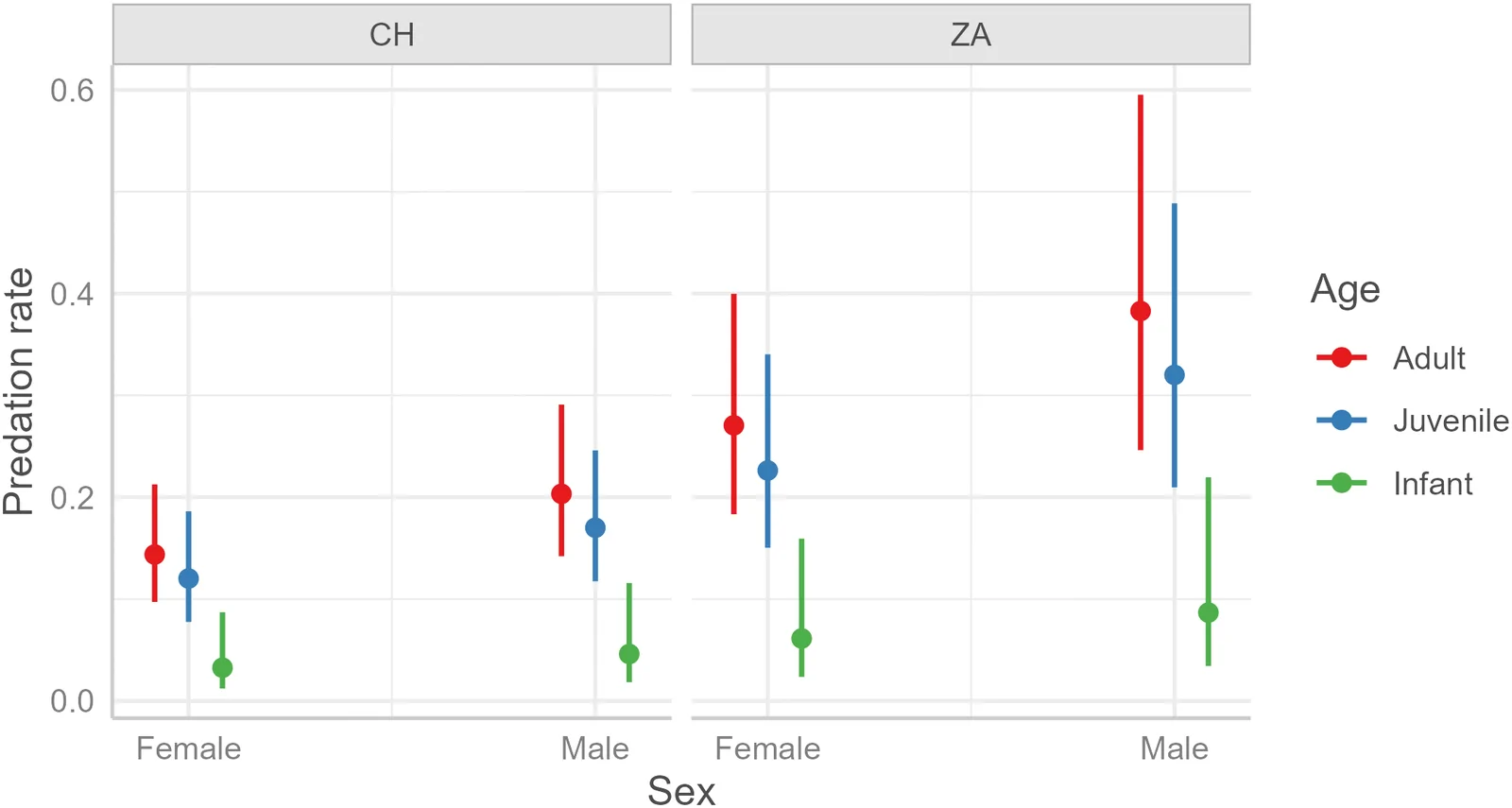
The likelihood of occurrence of a predation event by group (CH: provisioned; ZA: unprovisioned), age class, and sex.

Individuals in the provisioned group consumed significantly less vertebrate prey than those in the unprovisioned group (ZA; *p* < 0.001; Table 4; Fig 2). The estimated rate of predation events per unit of observation time in the non-provisioned group was 1.88 times higher than in the provisioned group. Infants consumed vertebrate prey at only one-fifth the rate of adults (*p* < 0.002), whereas juveniles did not differ significantly from adults (Fig 3). Males showed higher rates of vertebrate consumption than females, with an estimated rate ratio of 1.4 (*p* = 0.049).

**Table 4:** Results of the zero-inflated negative binomial generalized linear mixed model testing predictors of vertebrate predation rate per unit of observation time.

Term	Estimate	SE	Lower CL	Upper CL	Wald’s	*p*-value
*Conditional model (Negative Binomial)*						
*(Intercept)*	−6.6802	0.199334	−7.07089	−6.28951	−33.5126	–
***Sex (Male)***	0.348003	0.176809	0.001463	0.694543	1.968241	0.04904*
*Age (Juvenile)*	−0.17863	0.174004	−0.51967	0.162411	−1.02659	0.304616
***Age (Infant)***	−1.48032	0.468656	−2.39886	−0.56177	−3.15864	0.001585*
***Group (ZA)***	0.632806	0.183204	0.273734	0.991879	3.454114	0.000552*
***Rainfall***	0.428193	0.169339	0.096294	0.760092	2.52861	0.011452*
***Fruits***	−0.49586	0.146094	−0.7822	−0.20952	−3.39411	0.000689*
*Insects*	−0.23761	0.14441	−0.52065	0.045427	−1.64539	0.099889
*Zero Inflation model*						
*(Intercept)*	25.13092	16.93205	−8.05529	58.31714	1.484222	–
***Temperature***	−2.11166	0.858397	−3.79409	−0.42924	−2.46001	0.013893*
***Humidity***	0.377545	0.130974	0.120841	0.634249	2.882603	0.003944*

Parameter estimates are shown for the conditional and zero-inflation components. Observation effort was accounted for by including log(number of scan samples) as an offset term. *n =* 1092. Random effects: ID (36); Month (45). Final model metrics: AIC = 1253.7; BIC = 1323.6; logLik = −612.8; deviance = 1225.7; df residuals = 1078. ZA: unprovisioned group. Asterisks indicate *p < 0.05.* Reference categories: Sex: female; Age: adult; Group: provisioned.

**Fig 4:**
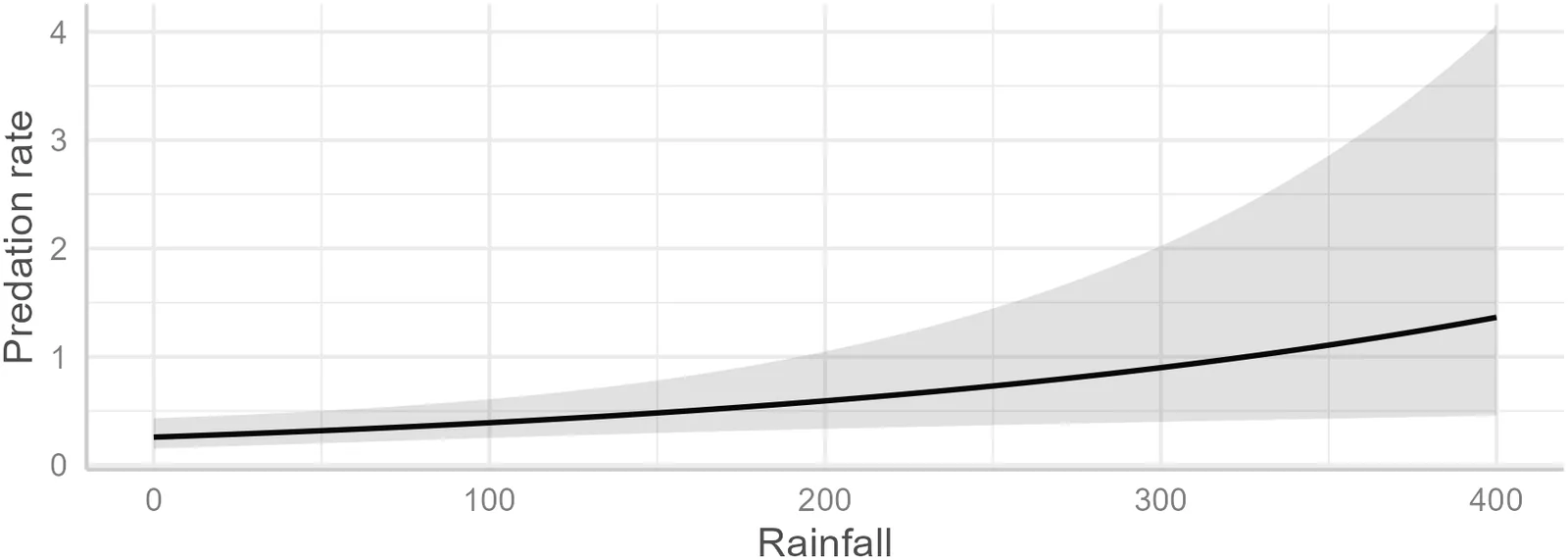
Effect of rainfall (mm) on the likelihood of a capuchin monkey preying on a vertebrate. Adjusted for variables kept fixed as logtime = 4.74; Age Class = Adult; Fruit availability = 289.89; Insect availability = 0; Temperature = 0, Humidity = 0.

**Fig 5:**
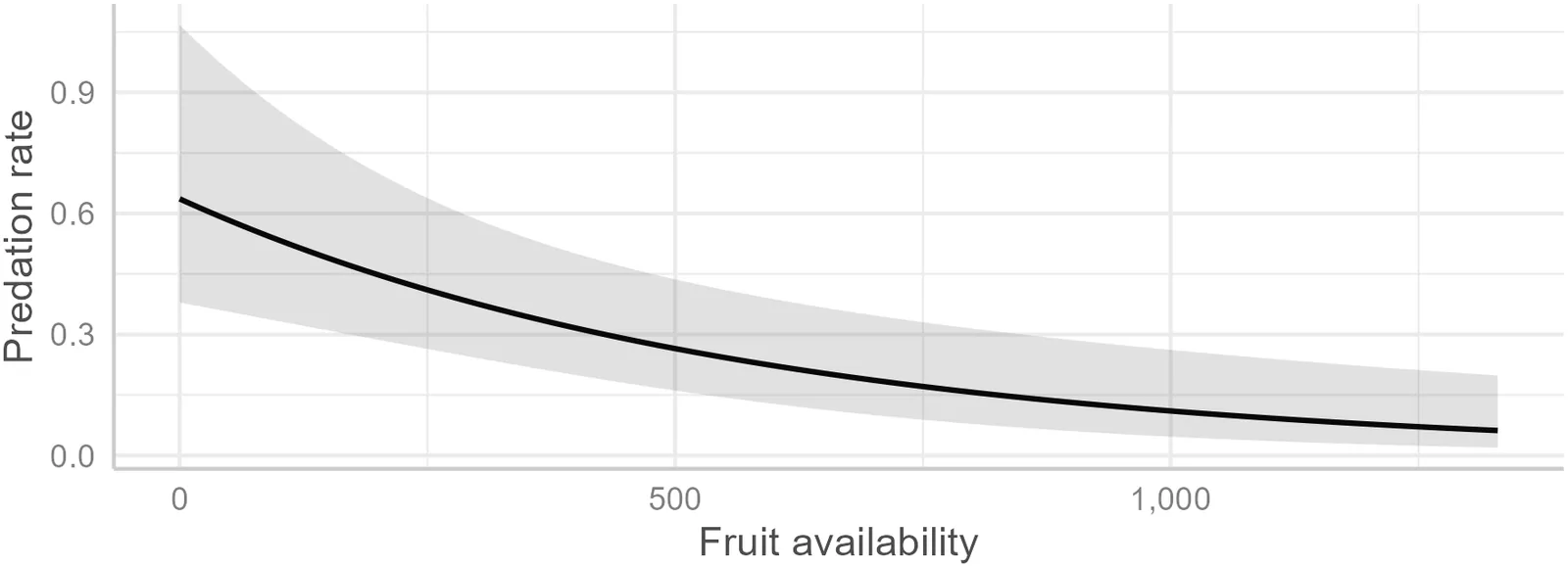
Effect of fruit availability (kg/ha) on capuchin vertebrate predation rate. Adjusted for variables kept fixed as logtime = 4.74; Age Class = Adult; Rainfall = 94.99; Insect availability = 0; Temperature = 0, Humidity = 0.

Rainfall positively affected the rate of predation events per unit observation time (*p* = 0.011; Fig 4). In contrast, fruit availability showed a strong negative association with predation rates (*p* < 0.001; Fig 5). Insect availability was not significantly associated with predation rate (*p* = 0.1). Temperature (*P* = 0.014) and humidity (*P* = 0.004) were used in a separate part of the model—the zero-inflation model. Temperature had a significant negative effect (*p* = 0.014): higher temperatures reduced the probability that an observation fell into the “always zero” state, making predation events more likely to be recorded. In contrast, humidity had a significant positive effect (*p* = 0.004): higher humidity increased the probability of an excess zero, i.e., conditions under which no predation events were observed.

### Model 2: Sub-model of mammal consumption

**Fig 6:**
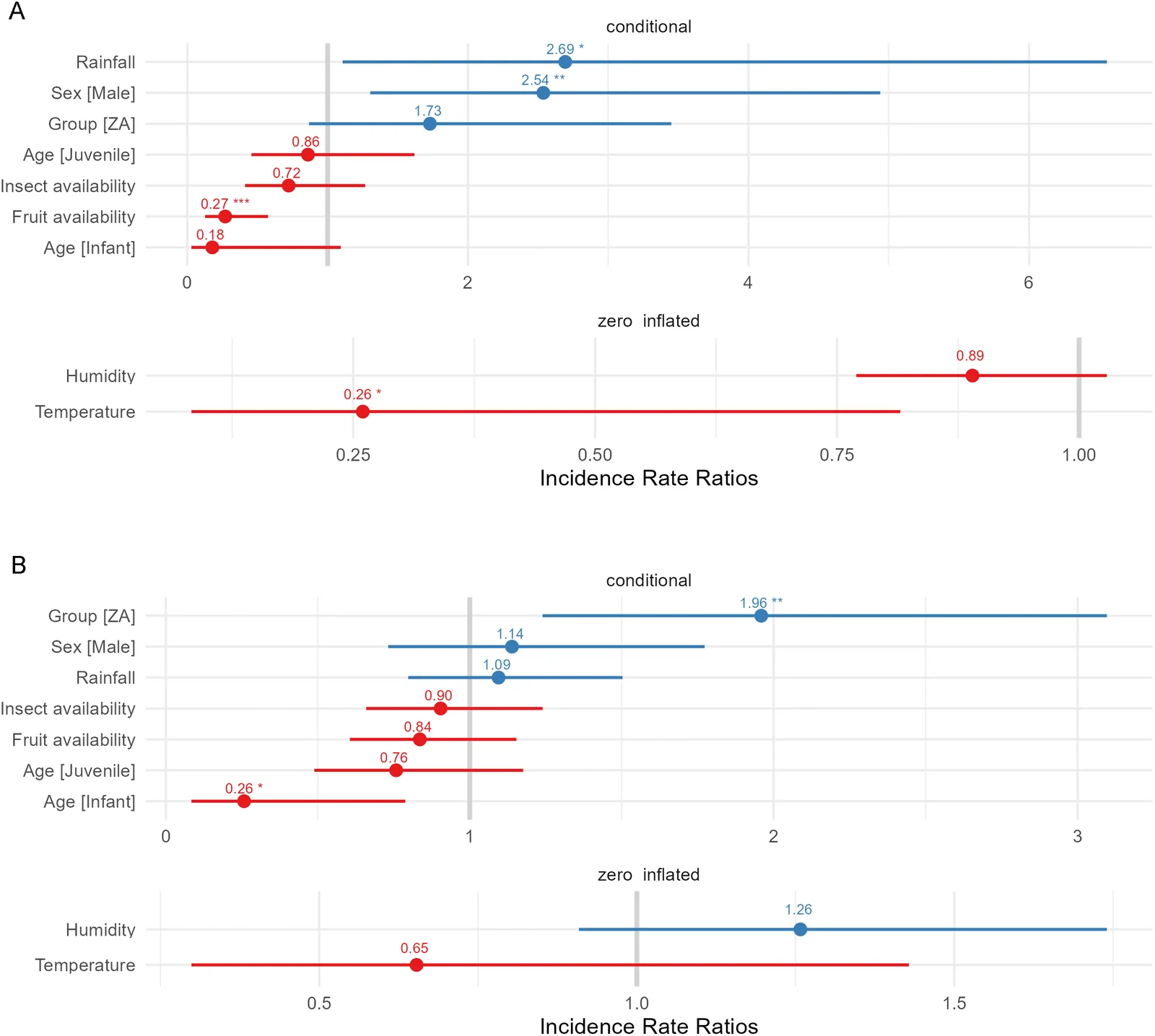
Contrasting sub-models with predation rate split by prey type: a) mammals; b) saurians. ZA: unprovisioned group.

The primary focus of this analysis was to model predation events involving mammals, namely rodents, marsupials, and bats (S2 Table; Fig 6a). We again employed a negative binomial mixed-effects model with a zero-inflation component to address overdispersion and excess zeros in the data, respectively. The model exhibited good fit statistics, with an AIC of 502.6, and several predictors showed significant associations with variation in mammalian predation rates.

Males showed significantly higher mammal predation rates than females (*p* = 0.006) (S2 Table). Age was not a statistically significant predictor of mammal predation rate. The difference between the provisioned and unprovisioned groups was also not significant. Increased rainfall (*p* = 0.029) and higher fruit availability (*p* < 0.001) were both significantly associated with higher and lower predation rates, respectively. Temperature significantly reduced the probability of excess zeros (*p* = 0.021), indicating that predation events were more likely to be observed at higher temperatures, while humidity showed a non-significant trend.

### Model 3: Sub-model of saurian predation

The same zero-inflated negative binomial regression model was employed to investigate the relationship between saurian predation events and group, individual and environment-related predictors (S3 Table; Fig 6b). Saurian predation showed a significant positive association with group (*p* = 0.004), with the unprovisioned group showing a higher saurian predation rate than the provisioned one. We did not find significant relationships between any environmental variable and predation rate. We also did not find a significant effect of sex or age over predation rate. Overall, our results suggest that saurian predation events are most strongly influenced by provisioning.

### Hunting and food transfer events

Hunting events were rarely observed. Out of 280 consumption events recorded by scan sampling for both groups, we observed hunting preceding consumption in 51 instances (see S2 Video). In all other cases, hunting was noticed as the prey was grasped, or inferred to have occurred from the presence of prey seen in a capuchin’s hand (see S3 Video). Consumption of vertebrate prey was significantly higher in type I events (individual, 82.14%, N = 230) than in type II events (collective, 17.86% N = 50) Chi-square, χ^2^ = 17,708, df = 2, *p* = 0.000). Of the 165 vertebrate predation episodes recorded by all occurrence sampling for both groups, 81.21% (n = 134) were episodes in which only one individual was seen consuming the prey item, whereas 18.87% (n = 31) were episodes in which two or more individuals were seen consuming the same prey item. The transfer of meat, or of other parts of the carcass (such as skin, bones, etc.) was observed in 32 of the 50 type II events (64%).

## Discussion

We reported on the most extensive record of bearded capuchin (*S. libidinosus*) vertebrate predation to date: 45 months of vertebrate consumption observations across two sympatric groups in Fazenda Boa Vista (Brazil). We showed that vertebrate consumption is habitual in these groups (*sensu* McGrew, 1992 [78]) and occurs at an average rate of 6.34 predation events per 100 hours. This behaviour was seen across all age classes (adults, juveniles, and infants) and both sexes. Both groups studied here were observed feeding on rodents, bats, lizards, adult birds and snakes, in line with other bearded capuchin populations [29,32]. However, we also recorded opossums, iguanas and bird eggs/nestlings as part of this species’ diet for the first time. Additionally, we observed both hunting events with more than one participating individual, as well as food transference events.

Our results revealed a strong association between food availability and the exploitation of small animal resources. First, in support of hypothesis 1, provisioned individuals exhibited lower vertebrate predation rates than unprovisioned ones. This effect was upheld when looking only at saurian prey, but not when looking only at mammalian prey. Provisioning has been shown to alter primates’ activity budgets, diets and ranging patterns by providing resources of a higher caloric content, availability and predictability than those found in their natural habitat [79,80]. Asquith (1989) [81] raised concerns about the lack of comparative data between provisioned and unprovisioned individuals in primate studies. Our research directly addressed this issue by comparing the predation patterns of provisioned and unprovisioned groups of sympatric capuchins.

Second, in support of hypothesis 2.2, we found vertebrate meat intake varies inversely with fruit abundance. This trend was upheld in the mammal-only model but not in the saurian-only model. No such association was found between insect abundance and vertebrate predation, countering hypothesis 2.1. This suggests small mammals may be particularly important sources of energy in wild bearded capuchins at Fazenda Boa Vista during during periods of fruit scarcity. These results support the “energy shortfall hypothesis”, which posits that animal-source foods are a particularly important source of energy for primates when plant-source foods become less available [1]. A similar mechanism has been proposed as a driver of increased meat consumption in the genus *Homo* [13]. As late Pliocene and early Pleistocene eastern African hominin environments became increasingly seasonally arid [13,82], the availability of plant-source foods that are key to many primates’ diet in forest habitats would have only been available intermittently, whereas animal resources would have remained accessible throughout the year [13]. This may have selected for a higher frequency of predatory behaviours in our lineage [13]. Bearded capuchin reliance on small vertebrates during fruit-poor periods supports this strategy as a possible predecessor and/or complement to large-prey hunting in humans in seasonal environments [3].

However, the population of Fazenda Boa Vista appears to be an exception in this regard: the most extensive review of animal predation across the primate order to date found little evidence in support of the energy shortfall hypothesis, even in species inhabiting seasonally extreme environments like savannas [1]. For instance, the hunting behaviours of chimpanzees in the savanna-woodlands of Fongoli, Senegal, are not explained by fruit scarcity [83]. Furthermore, chimpanzees in some forested environments appear to hunt more often during food-rich rather than food-poor seasons, possibly because they can afford the energetic losses of an unsuccessful hunt when they have another easily obtainable source of energy: fruit [1,50,84]. White-faced capuchins in Santa Rosa (Costa Rica) do consume more vertebrate prey in the dry season, when fruit is scarce, but this is likely due to the concurrent increase in preferred prey availability, as coati infants are born at this time [1,25].

It is important to note that, in this study, we combined the availability of palm nuts with that of other fruits. The kernels of palm nuts constitute a key food item for the capuchins at Fazenda Boa Vista: they are rich in lipids and protein, and provide a reliable source of energy and a stable protein intake [85]. It is therefore possible that the observed inverse relationship between vertebrate predation and fruit availability is strongly influenced by fluctuations in nut consumption and availability. Additionally, it is possible that the measure of fruit availability used in the present study does not reflect variation in capuchin fruit consumption (e.g., capuchins may consistently access some fruits regardless of their availability, or they may not increase their consumption of non-preferred fruits despite their increased availability).

Environmental factors also had a significant effect over capuchin vertebrate consumption patterns, in support for hypothesis 3.2 rather than hypothesis 3.1. Increased rainfall was linked to increased vertebrate consumption rates in this population. Furthermore, predation events were more likely to be recorded at higher temperatures and less likely to be recorded at higher humidity levels. Rather than reflecting a direct effect of these environmental variables over vertebrate predation, our results likely reflect the presence of a mediating variable: the availability of preferred vertebrate prey species. It is possible that periods of higher temperatures and rainfall, as well as lower humidity, coincide with the birthing season of some prey species. These seasons have been linked with increased vertebrate predation rates in other capuchin populations, as they are characterized by more readily abundant and easier-to-catch prey: infants [25].

We also found strong effects of age and sex on capuchin vertebrate consumption rates. Across both study groups, infants–but not juveniles–had lower vertebrate consumption rates than adults, providing support for hypothesis 4.2 rather than hypothesis 4.1. Additionally, males had higher predation rates than females, in support for hypothesis 5.1 rather than hypothesis 5.2. These sex differences are similar to those documented at other long-term capuchin monkey field sites [22,25,29,31,49], as well as in baboons [86–91] and chimpanzees [1]. Improving our understanding of these patterns in robust capuchins allows us insights beyond those provided by chimpanzees, baboons and gracile capuchins, on whom much of the research on the subject has focused. That males appear to capture more vertebrates than females across all the aforementioned primate taxa presents an interesting pattern, especially considering ongoing debates about sex biases in hunting among modern unindustrialized societies [92,93]. Across many of these communities, men hunt more often than women on average [93–96]. Although there is variation between societies and women regularly hunt in many [92,97–99] these sex differences appear to be present and accurately reported [93].

Aside from increased caloric intake demanded by male bearded capuchin’s body size compared to that of females [49,53], the observed sex differences in predation rates may be explained by a risk-averse foraging strategy in females compared to males [52]. For instance, all snake predation events were performed by males in our study population, which may reflect female avoidance of potentially dangerous prey. In chimpanzees, females feed significantly more on insects, such as termites and ants, than males [52,100]. This has been proposed as a strategy that combines longer investment of time to access high quantities of protein and calories from safer, more reliable and less costly sources of food than vertebrate hunting [52,100,101]. Gilby *et al*., (2017) [52] posited this strategy might have been present in the last common ancestor between the *Pan* lineage and the human lineage, possibly preceding the appearance of the sexual division of labor in modern humans. In unindustrialized societies today, female risk aversion has also been posited as a driver of the sexual division of labor [102]. By extending the comparison beyond chimpanzees and humans, capuchins may provide insights on the extent to which sex biases in vertebrate predation may be a general response to the risks and energetic trade-offs of hunting. Like chimpanzees, female white-faced capuchins tend to consume more invertebrates than males [49] and insects are a vital source of energy for them, particularly during lactation [21]. We did not evaluate differences in invertebrate predation rate between bearded capuchin males and females; future research should aim to bridge this gap. Convergent sex-biases in invertebrate and vertebrate access across distantly related primate taxa might suggest that female avoidance of high-risk prey may be a recurrent strategy among vertebrate-eating primates.

The majority of the hunting events we observed, as well as the consumption of captured prey, were carried out by one individual rather than by two or more. Instances of prey transfer did occur in our population, in support of hypothesis 6. This mirrors observations in the bearded capuchins from Serra da Capivara National Park [29] and Serra das Almas Private Reserve of Natural Heritage [32] in Brazil. However, many of the small vertebrates captured by a group of hunters were monopolized and consumed by only one individual. Factors such as dominance rank may influence who gains priority access to prey, as subordinate individuals may be supplanted [25], while kinship ties could play a role in determining whether prey is shared [103]. Nevertheless, most predation events were performed alone and in the absence of other group members. This suggests that collaborative hunting may not be necessary for the capture of small prey in bearded capuchins. Instead, solitary hunting and eating may minimize intraspecific competition for limited food. Many small vertebrates may not provide enough nutrients, proteins and energy for more than one individual, potentially discouraging cooperative hunting and sharing. For example, larger prey like adult squirrels appeared to be shared by white-faced capuchins in Santa Rosa, whereas nestlings were usually entirely eaten by one captor [25]. Similarly, smaller prey sizes have been reported to discourage cooperative hunting in chimpanzees [104].

Capuchin monkeys in the present study consumed vertebrate’s brains and viscera first, and then (but not always) the rest of the body. Capuchins’ preferential access of these tissues may respond to their high lipid content compared to other prey bodily tissues [105,106]. For example, chimpanzees at Gombe National Park consistently consume the brains of subadult prey and the viscera of adult prey, whose skulls are more robust and harder to crack, before moving to the rest of their body [107,108]. This behavior possibly allows them to maximize the fats and nutrients obtained before the carcass is stolen by conspecifics [107,108]. A similar consumption order has been observed in Taï [51] and Fongoli [109] chimpanzees. The scavenging of in-bone nutrients, such as those provided by the brain, have been posited as one of the stepping-stones towards the emergence of the human predatory pattern [3]. Like bearded capuchins and chimpanzees, hominins may also have preferentially accessed the brains of small prey, and later began scavenging these in-bone nutrients from large carcasses before they were able to hunt large prey [3]. However, our results differ from patterns observed in the white-faced capuchins of Santa Rosa, Costa Rica, who appear to consume the viscera first and typically discard the head [27].

While primates accessing prey of the same size or larger than themselves is only seen in humans [3], the consumption of small vertebrates is seen across a large number of taxa [1]. On one hand, this may reflect an ancient opportunistic strategy for the obtention of energy, nutrients and/or proteins. On the other hand, small vertebrate predation may have emerged through convergent evolution in several branches of the primate order. Hunting small prey does not necessarily require tools or cooperation with group members to be successful, which may contribute to the persistent presence of this behaviour in the primate order [1], including in hominins [14–18]. Despite the emergence of the human predatory pattern, the consumption of small vertebrates has persisted in the human lineage, and small game forms part of the diet of many unindustrialized societies today [19,20]. The presence of this form of vertebrate consumption across primate taxa supports the possibility that small prey provides an important, reliable and less costly source of nutrients than larger prey.

### Future directions

We recommend that early faunal assemblages composed of small vertebrates should be re-examined closely for evidence of damage inflicted by predation. Future capuchin monkey studies investigating vertebrate consumption should record the order in which different parts of the animal are consumed and document the taphonomy of these processes, so that we may identify the traces that small vertebrate predation leaves behind after the animal is consumed and abandoned, and thus build a better picture of the signatures we should be searching for in the fossil record. This will allow us to identify how patterns of consumption have changed through time in assemblages with hominins and other fossil primate presence.

In addition, based on our results and the open questions remaining, we recommend the following directions or suggestions for future research:

(1) Future studies on wild primate dietary patterns must strongly consider the potential influence of planned or unplanned (e.g., crop raiding, feeding on garbage) provisioning on predatory behaviours, as our results suggest primates may be less inclined to access animal foods when provisioned.
(2) Future research should aim to clarify whether the association between fruit abundance and vertebrate consumption observed in the Fazenda Boa Vista capuchins truly supports the energy shortfall hypothesis, or if said pattern is a result of opportunistic predation of seasonally abundant small mammals. Additionally, future studies should aim to directly quantify temporal variations in fruit consumption in capuchins and test their effect on vertebrate consumption rates.
(3) Future work should distinguish between the effects of rainfall, temperature, and humidity on capuchin vertebrate predation rates and the potential effect of increased prey availability during seasonal birthing periods. This may be done by directly measuring the effects of vertebrate prey abundance over capuchin vertebrate predation rates, for example.
(4) Future research might look into whether prey size may be a predictor of the likelihood of group hunting and sharing in bearded capuchins, as suggested by our observations.
(5) Further research should study the sequence in which other capuchin species consume prey body parts to provide a more nuanced understanding of the factors shaping the contrasting prey-consumption strategies observed in our study population and the capuchin populations in other sites [107].

## Supporting information

S1 File Cleaning and pre-processing. (DOCX)

S2 File Study Data. (CSV)

S1 Fig Full dataset, showing all predation events per individual and month. The size of the circles indicates the number of events (from 0 to 6) in each month and the circle color indicates the age class to which the individual belongs each month.
(TIF)

S2 Fig Predations events across the study period 56 months, starting in May 2006. **N**otice the 11 months gap from May 2008 to March 2009. Group information is also provided.
(TIF)

S3 Fig Full dataset after cleaning. Includes 10 fewer individuals, showing all predation events per individual and month. The sex of each individual is indicated by color (red = male, blue = female).
(TIF)

S1 Table Variance Inflation Factors (VIF) for the Consumption Model. VIF values < 5 indicate low multicollinearity; values near 1 indicate nearly no correlation between predictors.
(DOCX)

S2 Table Sub-model of mammalian consumption. Number of observations: 1092, random effects: ID (36); Month (45). Final model metrics: AIC = 502.6; BIC = 572.5; logLik = −237.3; deviance = 474.6; df residuals = 1078.
(DOCX)

S3 Table Sub-model of saurian consumption. Number of observations: 1092, random effects: ID (36); Month (45). Final model metrics: AIC = 920.3; BIC = 990.2; logLik = −446.1; deviance = 892.3; df residuals = 1078.
(DOCX)

S1 Video Vertebrate consumption. (a) A young capuchin eating a vertebrate; (b) Capuchin feeding on a lizard. This video was filmed by Alessandro Albani for the documentary “The bearded capuchin monkeys of Fazenda Boa Vista” (https://www.youtube.com/watch?v=7ojU43JgpYo), not recorded during the study, but it illustrates what we report here.
(ZIP)

S2 Video A hunting event of Type II (collective). Four young capuchins hidden in a bush attack an Iguana (*Iguana iguana*) passing nearby. One grasps and detaches the far end of the iguana tail. Meat transfer should have occurred while the capuchins were into the bush. However, it is important to note that sooner or later we observed all four of them eating parts of the iguana. This video was filmed by Alessandro Albani for the documentary “The bearded capuchin monkeys of Fazenda Boa Vista” (https://www.youtube.com/watch?v=7ojU43JgpYo), not recorded during the study, but it illustrates what we report here.
(ZIP)

S3 Video Food transfer. (a) A Subadult male has captured a great kiskadee (*Pitangus sulphuratus*) and moves in the canopy holding its carcass. (b) Other group members show interest towards it and approach the possessor. (c) Food transfer may occur with the possessor tolerating the recipient to take a piece of the prey. (d) An infant, who has obtained mostly bones with very little meat, is chewing the same kiskadee initially captured by the subadult male. This video was filmed by Alessandro Albani for the documentary “The bearded capuchin monkeys of Fazenda Boa Vista” (https://www.youtube.com/watch?v=7ojU43JgpYo), not recorded during the study, but it illustrates what we report here.
(ZIP)

S1 Checklist Inclusivity in global research information. Additional information regarding the ethical, cultural, and scientific considerations specific to inclusivity in global research in this study.
(DOCX)
