# Supplementary material for: Small vertebrate consumption by capuchin monkeys (Sapajus libidinosus): Insights into the human predatory pattern
Source: PLoS One. 2026 Aug 26;21(8):e0355204. doi: 10.1371/journal.pone.0355204 (PMC13514029; doi:10.1371/journal.pone.0355204)
Supplement: Supplementary file 2 [file pone.0355204.s015.pdf]

PLOS One

Small vertebrate consumption by capuchin monkeys (Sapajus libidinosus): Insights into the human predatory pattern

--Manuscript Draft--

**Manuscript Number:**	PONE-D-25-66742R1
**Article Type:**	Research Article
**Full Title:**	Small vertebrate consumption by capuchin monkeys (Sapajus libidinosus): Insights into the human predatory pattern
**Short Title:**	Small vertebrate consumption by capuchin monkeys
**Corresponding Author:**	Susana Carvalho, PhD<br>Gorongosa National Park<br>Beira, MOZAMBIQUE
**Keywords:**	capuchin behaviour, predatory patterns, foraging ecology, vertebrate consumption, availability of resources
**Abstract:**	The consumption of small vertebrates occurs to varying degrees among many non-human primates and may have constituted an important source of nutrition for early hominins, particularly during periods of food scarcity, providing a stepping stone to the exploitation of larger animals. Capuchin monkeys regularly consume small vertebrates across their range. Expanding our knowledge of the contribution of animal matter to these primates' diets can shed light on the proximate and ultimate drivers of small vertebrate consumption across primate taxa and help evaluate hypotheses on the role of small prey exploitation in human evolution. We report on the most extensive record of bearded capuchin (Sapajus libidinosus) vertebrate predation so far available: 45 months of observations of vertebrate predation in two sympatric groups in Fazenda Boa Vista, Brazil, one of which was provisioned daily. We estimated the effect of provisioning, sex, age class, fruit and insect availability, rainfall, humidity, and temperature on predation rates using a zero-inflated negative binomial algorithm with random effects. We found mammal and saurian consumption is habitual in this population of S. libidinosus, and prey brains and viscera tend to be accessed before the rest of the body. Predation rates were higher in males compared to females and in adults compared to infants across groups. Our results also identified small vertebrates as key energy sources for bearded capuchins under decreased fruit availability and show that provisioning reduces predation rates. Future work should assess small vertebrate predation as a possible stepping stone in the emergence of human exploitation of large animals by re-analyzing fossil faunal assemblages for evidence of small vertebrate predation and identifying the traces left by the exploitation of small prey in capuchins and other extant primates through neo-taphonomy.
**Order of Authors:**	Susana Carvalho, PhD
	Noemi Spagnoletti, PhD
	João D'Oliveira Coelho, PhD
	Megan Beardmore-Herd, PhD
	Olivia Mendonça-Furtado, PhD
	Michele Verderane, PhD
	Joana Prieto, MSc
	Dominique I. Cavallaro, MSc
	Patricia Izar, PhD
	Elisabetta Visalberghi, PhD
**Response to Reviewers:**	Reviewer #1<br><br>1. Hypothesis #6, Necessity:<br>I believe that this hypothesis is more linked to the absence or reduction of the fruit and

invertebrate availability than to environmental factors. We need to think (ecologically) that such variables can indirectly affect the fruit and invertebrate availabilities. So, the better fashion - I think - to test or think is associating with the food resources. The variable influencing diet and behaviour is food items and not rainfall, temperature, and humidity. These can or not influence the appearance of fruit and invertebrates in a given site. Furthermore, to test this hypothesis, perhaps it is better to relate vertebrate consumption with fruit consumption. Thus, the authors could demonstrate the necessity to exploit other food resources.

Response: Thank you for your feedback. We have edited the framing of this hypothesis so that it does not address necessity, but rather simply tests for environmental effects over predation rates in a way suggested by Reviewer #2. We hope the new framing of our hypothesis can better address this concern.

2. Environmental variables:
The manuscript works with three variables – rainfall, temperature, and humidity – to potentially explain the variation in vertebrate consumption by capuchins. However, two of these variables (rainfall and humidity) will have an effect on fruit and insect availability. So, I do not believe that both can contribute to the understanding of the findings. Thus, to test

Response:

3. Correlations among predictor variables were assessed using Spearman's rank correlation coefficient and variance inflation factors. Spearman's ρ indicated only low to moderate pairwise associations, and all VIF values were below 1.25, suggesting negligible multicollinearity. The strongest association was a moderate correlation between insect availability and rainfall ($\rho=0.45$). However, low VIF values indicated that the model was still able to distinguish between the seasonal increase in insect availability and the direct effect of rainfall on capuchin predation activity. In addition, model comparison based on AIC values showed that models including both environmental variables (rainfall, temperature, and humidity) and food availability variables (fruit and insect availability) consistently outperformed models including environmental variables alone. Because our a priori model selection procedure was based on comparing biologically motivated candidate models and retaining the model with the strongest AIC support, both the environmental variables and the fruit and insect availability variables were therefore kept in the final model. We have added an explicit clarification of this procedure in the methods (subsection Data analysis: statistical modelling) and a supplementary table with VIF values (S1 Table).

4. Energy shortfall hypothesis:
I believe that a correlation between vertebrate consumption vs. fruit consumption could help us to understand this hypothesis. The fruit availability is a great measure. But, it does not demonstrate whether the fruit consumption is (positively or negatively) linked to vertebrate intake. However, it needs to know if fruit consumption is associated with vertebrate consumption.

Response: Thank you for your feedback. While we appreciate that fruit consumption would have been a very interesting covariate to include in our model, we are not able to collect the necessary data retrospectively. However, we included and expanded on this limitation in our Discussion.

5. Humidity:
I would like to see a discussion related to the influence of humidity on the probability of an excess zero in the predation events. If such a variable was measured and incorporated in the study (i.e., data analysis) is necessary to discuss it.

Response: Thank you for your feedback. We have now included a discussion of the influence of humidity on this variable in our Discussion section.

6. Figures:
I do not know the reason, but the figures are not included in the manuscript to review. So, it was not possible to evaluate some scenarios inserted in the text.

Response: We have now included all relevant figures.

Reviewer #2

1. Introduction:
The introduction to this manuscript would benefit from major revisions to tighten it up. While relevant literature is cited, the current structure reads more like an information dump instead of a focused funnel toward the hypotheses. Redundant and overly dense sentences obscure the core theoretically background and the importance of the hypotheses the authors propose to text. Additionally, several references appear to be incorrectly formatted starting in the Into and persisting throughout the manuscript. For instance, line 169 reads "When reviewed by (1),…" where an author name should appear. The paper as whole would benefit from a careful review for these in-text citations.

Response: Thank you for your feedback. The introduction was majorly revised and edited according to these comments, better structured as the "focused funnel toward the hypotheses" the reviewer suggested. We have also reviewed all in-text citations and edited incorrect formatting.

2. Hypotheses:
The authors present a thoughtful hypothesis framework to address the drivers of vertebrate predation in these groups of capuchins. Testing predation rates between provision and non provisioned groups is well conceived. The protein in availability hypothesis is reasonable since predation might be driven by the need for macro and micronutrient needs.

Hypothesis 1 and hypothesis 2.1 appeared to be contradictory as written. Hypothesis one predicts that males will have higher vertebrate predation rates because of their increased body mass. Whereas hypothesis 2.1 predicts that females will have higher vertebrate predation rates than adult males because of reproduction and lactation needs. Would a better test of hypothesis 2.1 be that pregnant or lactating females would have increased vertebrate predation rates than non-pregnant non lactating females? Or, and it's not clear if this possible from that dataset, since you know individuals in the dataset could you compare individuals of similar body mass?

Hypotheses 5 and 6 should be presented as a null and alternative. As written these two hypotheses are testing if predation rates are influenced by environmental factors. The authors are then interpreting whether it's opportunistic or necessity. In my opinion this would better be presented as a null with an alternative that environment impacts predation rates and save the discussion of opportunity and necessity in the context of different prey types for the discussion.

The last hypothesis concerns food transfer and hunting and wild capuchins. This hypothesis is presented differently than the previous hypotheses, with the hypothesis buried within the text of the paragraph. I wonder if the authors could weave in the discussion of hunting and food transfer among primates in general and capuchin specifically somewhere in the main part of the introduction prior to introducing their hypotheses.

Response:

Thank you for your feedback. Regarding hypotheses 1 and 2.1 (now presented as 1.1 and 1.2 in the revised manuscript), testing whether pregnant or lactating females have increased vertebrate predation rates than non-pregnant or non-lactating females is not possible from the dataset. However, we have now presented the two hypotheses as alternatives concerning sex biases on predation rates: either males will have higher predation rates, or females will (or neither of the two). We hope this revised format can clarify the need for alternative hypotheses regarding sex biases in predation rates.

Regarding hypotheses 5 and 6 (now presented as 5.1 and 5.2), we have re-framed these as null and alternative as suggested by the reviewer.

Regarding the hypothesis on food transfer and hunting: we have included this

hypothesis with the others and added the discussion on hunting and food transfer in the main part of the introduction prior to the hypotheses, as suggested by the reviewer.

3. Methods:
The statistical modeling approach is used here appears appropriate and well justified. Maybe I missed this, but why is rainfall in the conditional model but not in the zero inflation model? Was that an a priori decision or was it from AIC comparisons? I assume, perhaps incorrectly, that humidity and rainfall or rainfall and fruit/insect availability covary somewhat. Did the authors test for correlations among predictors? Could this be addressed with a sentence or two in the methods?

Response:

Rainfall is in the conditional model but not in the zero inflation model because this model structure improved BIC and AIC substantially (and it also helped a lot with the overall modelling architecture, as models tended to not be able to converge in the opposite solution).

Correlations among predictor variables were assessed using Spearman's rank correlation coefficient and variance inflation factors. Spearman's ρ indicated only low to moderate pairwise associations, and all VIF values were below 1.25, suggesting negligible multicollinearity. The strongest association was a moderate correlation between insect availability and rainfall ($\rho=0.45$). However, low VIF values indicated that the model was still able to distinguish between the seasonal increase in insect availability and the direct effect of rainfall on capuchin predation activity. In addition, model comparison based on AIC values showed that models including both environmental variables (rainfall, temperature, and humidity) and food availability variables (fruit and insect availability) consistently outperformed models including environmental variables alone. Because our a priori model selection procedure was based on comparing biologically motivated candidate models and retaining the model with the strongest AIC support, both the environmental variables and the fruit and insect availability variables were therefore kept in the final model. We have addressed this explicitly in the methods (subsection Data analysis: statistical modelling) and added a supplementary table with VIF values (S1 Table).

4. Results:
With the caveat that I can't evaluate the results completely without the figures, the results are clearly reported.

Response: We have now included all relevant figures.

5. Discussion:
I have no major concerns about the discussion. A minor suggestion is to integrate Table 5 into the results.

Response: Thank you for your feedback. However, we believe Table 5 is better suited for a visual summary of results in the Discussion section than for the Results. The Results already thoroughly detail the information in Table 5, so including it in this section would be repetitive. In contrast, the presence of Table 5 in the Discussion makes the dissection of the main results of this paper easier for the reader, as there are many hypotheses addressed in this article.

Academic editor

1. Please ensure that your manuscript meets PLOS ONE's style requirements, including those for file naming. The PLOS ONE style templates can be found at https://journals.plos.org/plosone/s/file?id=wjVg/PLOSOne_formatting_sample_main_body.pdf and https://journals.plos.org/plosone/s/file?id=ba62/PLOSOne_formatting_sample_title_authors_affiliations.pdf.

Response: All style templates have been checked and applied. Image files have been re-formatted as per instructions.

	2. Please include a complete copy of PLOS' questionnaire on inclusivity in global research in your revised manuscript. Our policy for research in this area aims to improve transparency in the reporting of research performed outside of researchers' own country or community. The policy applies to researchers who have travelled to a different country to conduct research, research with Indigenous populations or their lands, and research on cultural artefacts. The questionnaire can also be requested at the journal's discretion for any other submissions, even if these conditions are not met. Please find more information on the policy and a link to download a blank copy of the questionnaire here: https://journals.plos.org/plosone/s/best-practices-in-research-reporting . Please upload a completed version of your questionnaire as Supporting Information when you resubmit your manuscript.<br><br>Response: The questionnaire has been completed and is uploaded as Checklist_S1.pdf. We reference this checklist in our updated Methods section, subsection "Inclusivity in global research".<br><br>3. In your Methods section, please provide additional information regarding the permits you obtained for the work. Please ensure you have included the full name of the authority that approved the field site access and, if no permits were required, a brief statement explaining why.<br><br>Response: Our "Ethical note" section has been updated to provide additional information on the permits obtained for the work and the authority that approved field access.<br><br>4. Thank you for stating in your Funding Statement:<br><br>"This work was supported by University La Sapienza of Rome (NS), EU FP6 NEST Programme ANALOGY (NS: No 029088), FAPESP (MPV: 06/51578-9, OMF: 08/52293-3 and PI: 06/51577-2 and 08/55684-3), CNPq; CAPES (NS: 017/2012), ISTC-CNR, Ethoikos srl. The funders had no role in study design, data collection and analysis, decision to publish, or preparation of the manuscript.".<br><br>Please provide an amended statement that declares *all* the funding or sources of support (whether external or internal to your organization) received during this study, as detailed online in our guide for authors at http://journals.plos.org/plosone/s/submit-now. Please also include the statement "There was no additional external funding received for this study." in your updated Funding Statement.<br><br>Please include your amended Funding Statement within your cover letter. We will change the online submission form on your behalf.<br><br>Response: We have included the amended Funding Statement in our cover letter (Cover Letter Plos One with Amended Funding Statement.docx).<br><br>5. Please upload a copy of Figure 1-6, to which you refer in your text. If the figure is no longer to be included as part of the submission please remove all reference to it within the text.<br><br>Response: We have now uploaded copies of Figures 1-6 as .tiff files and in correct formatting.<br><br>6. If the reviewer comments include a recommendation to cite specific previously published works, please review and evaluate these publications to determine whether they are relevant and should be cited. There is no requirement to cite these works unless the editor has indicated otherwise.<br><br>Response: All citation recommendations by reviewers were evaluated as relevant and were included as references in the updated manuscript.
**Additional Information:**	
**Question**	**Response**
**Financial Disclosure**	Yes

Enter a financial disclosure statement that describes the sources of funding for the work included in this submission. Review the submission guidelines for detailed requirements. View published research articles from *PLOS ONE* for specific examples.<br><br>This statement is required for submission and **will appear in the published article** if the submission is accepted. Please make sure it is accurate.<br><br>**Funded studies**<br>Enter a statement with the following details:<br>• Initials of the authors who received each award<br>• Grant numbers awarded to each author<br>• The full name of each funder<br>• URL of each funder website<br>• Did the sponsors or funders play any role in the study design, data collection and analysis, decision to publish, or preparation of the manuscript?<br><br>Did you receive funding for this work?	
Please add funding details.<br>as follow-up to "**Financial Disclosure**<br><br>Enter a financial disclosure statement that describes the sources of funding for the work included in this submission. Review the submission guidelines for detailed requirements. View published research articles from *PLOS ONE* for specific examples.<br><br>This statement is required for submission and **will appear in the published article** if the submission is accepted. Please make sure it is accurate.	This work was supported by University La Sapienza of Rome (NS), EU FP6 NEST Programme ANALOGY (NS: No 029088), FAPESP (MPV: 06/51578-9, OMF: 08/52293-3 and PI: 06/51577-2 and 08/55684-3), CNPq; CAPES (NS: 017/2012), ISTC-CNR, Ethoikos srl. The funders had no role in study design, data collection and analysis, decision to publish, or preparation of the manuscript.

Powered by Editorial Manager® and ProduXion Manager® from Aries Systems Corporation

**Funded studies**<br>Enter a statement with the following details:<br>• Initials of the authors who received each award<br>• Grant numbers awarded to each author<br>• The full name of each funder<br>• URL of each funder website<br>• Did the sponsors or funders play any role in the study design, data collection and analysis, decision to publish, or preparation of the manuscript?<br><br>Did you receive funding for this work?"	
Please select the country of your main research funder (please select carefully as in some cases this is used in fee calculation).<br>as follow-up to "**Financial Disclosure**<br><br>Enter a financial disclosure statement that describes the sources of funding for the work included in this submission. Review the submission guidelines for detailed requirements. View published research articles from *PLOS ONE* for specific examples.<br><br>This statement is required for submission and **will appear in the published article** if the submission is accepted. Please make sure it is accurate.<br><br>**Funded studies**<br>Enter a statement with the following details:<br>• Initials of the authors who received each award<br>• Grant numbers awarded to each author<br>• The full name of each funder<br>• URL of each funder website<br>• Did the sponsors or funders play any role in the study design, data collection and analysis, decision to publish, or preparation of the manuscript?<br><br>Did you receive funding for this work?"	ITALY - IT
**Competing Interests**	The authors have declared that no competing interests exist.

Use the instructions below to enter a competing interest statement for this submission. On behalf of all authors, disclose any competing interests that could be perceived to bias this work—acknowledging all financial support and any other relevant financial or non-financial competing interests.<br><br>This statement is required for submission and **will appear in the published article** if the submission is accepted. Please make sure it is accurate and that any funding sources listed in your Funding Information later in the submission form are also declared in your Financial Disclosure statement.<br><br>View published research articles from *PLOS ONE* for specific examples.<br><br>**NO authors have competing interests**<br><br>Enter: *The authors have declared that no competing interests exist.*<br><br>**Authors with competing interests**<br><br>Enter competing interest details beginning with this statement:<br><br>*I have read the journal's policy and the authors of this manuscript have the following competing interests: [insert competing interests here]*<br><br>* typeset	
**Data Availability**<br><br>Authors are required to make all data underlying the findings described fully available, without restriction, and from the time of publication. PLOS allows rare exceptions to address legal and ethical concerns. See the PLOS Data Policy and FAQ for detailed information.	Yes - all data are fully available without restriction

A Data Availability Statement describing where the data can be found is required at submission. Your answers to this question constitute the Data Availability Statement and **will be published in the article**, if accepted.<br><br>**Important:** Stating 'data available on request from the author' is not sufficient. If your data are only available upon request, select 'No' for the first question and explain your exceptional situation in the text box.<br><br>Do the authors confirm that all data underlying the findings described in their manuscript are fully available without restriction?	
Additional data availability information:	
**Ethics Statement**<br><br>Enter an ethics statement for this submission. This statement is required if the study involved:<br><br>• Human participants<br>• Human specimens or tissue<br>• Vertebrate animals or cephalopods<br>• Vertebrate embryos or tissues<br>• Field research<br><br>Write "N/A" if the submission does not require an ethics statement.<br><br>General guidance is provided below. Consult the submission guidelines for detailed instructions. **Make sure that all information entered here is included in the Methods section of the manuscript.**	This study includes field research that collected data on non-human primates and adhered to all the regulations and ethical guidelines of the countries involved in the study. The study was observational and no direct interactions were held between researchers and non-human primates. This study did not involve the collection of plant, animal or other materials from a natural setting. The research adhered to the Brazilian government permit (SisBio #28689) in accordance with the Brazilian legislation (law #11.794, October 8, 2008) and to the Brazilian National Council for Scientific and Technological Development (CNPq) authorization for Scientific expedition, under the Project CMC 000111, which included the permission from the landowners Marino Gomes de Oliveira and Maria Fonseca Gomes de Oliveira to conduct research in their field site. We adhered to the American Society of Primatologists' principles for the ethical treatment of primates.

Powered by Editorial Manager® and ProduXion Manager® from Aries Systems Corporation

**Format for specific study types**<br><br>**Human Subject Research (involving human participants and/or tissue)**<br>• Give the name of the institutional review board or ethics committee that approved the study<br>• Include the approval number and/or a statement indicating approval of this research<br>• Indicate the form of consent obtained (written/oral) or the reason that consent was not obtained (e.g. the data were analyzed anonymously)<br><br>**Animal Research (involving vertebrate animals, embryos or tissues)**<br>• Provide the name of the Institutional Animal Care and Use Committee (IACUC) or other relevant ethics board that reviewed the study protocol, and indicate whether they approved this research or granted a formal waiver of ethical approval<br>• Include an approval number if one was obtained<br>• If the study involved *non-human primates*, add *additional details* about animal welfare and steps taken to ameliorate suffering<br>• If anesthesia, euthanasia, or any kind of animal sacrifice is part of the study, include briefly which substances and/or methods were applied<br><br>**Field Research**<br><br>Include the following details if this study involves the collection of plant, animal, or other materials from a natural setting:<br>• Field permit number<br>• Name of the institution or relevant body that granted permission	
Data Availability<br><br>Before publication, Authors are required to make fully available and without restriction all data underlying their findings. Sharing the data underlying your findings increases the visibility, credibility, and impact of your work, while supporting transparency and future scientific discovery. Please see our PLOS Data Policy page for detailed information on this policy.</p><br>A Data Availability	All relevant data are within the paper and its Supporting Information files.

Statement, detailing where the data can be accessed, is required at first submission. Insert your Data Availability Statement in the box below.</p><br><br>Please see the data reporting of our submission guidelines for instructions on what you need to include in your Data Availability Statement. Examples of acceptable Data Availability Statements are included here for reference:</p><br> <br> - The minimal data set [and accompanying code, where generated] is available at [repository name] via [DOI hyperlink] <br> - No data was generated by this study. The following existing data sources were used: [dataset name] from [dataset location] available via [web address or DOI hyperlink]. <br> - For Study Protocols: No datasets were generated or analysed during the current study. All relevant data from this study will be made available upon study completion. <br> <br><br><i>PLOS allows rare exemptions to address legal and ethical concerns. If you have legal or ethical restrictions, please use the box below to detail these in full sentences for the Journal team to consider.</i></p><br><br>This statement will be typeset if the manuscript is accepted for publication.</p>	

Powered by Editorial Manager® and ProduXion Manager® from Aries Systems Corporation

Manuscript Click here to access/download;Manuscript;Manuscript.docx

Small vertebrate consumption by capuchin monkeys (*Sapajus libidinosus*): Insights into the human predatory pattern

Susana Carvalho[1,2¶*], Noemi Spagnoletti[3,4,5¶], João d'Oliveira Coelho[1,2,6], Megan Beardmore-Herd[2,6], Olívia Mendonça-Furtado[3], Michele Verderane[3], Joana Prieto[7], Dominique I. Cavallaro[8], Patrícia Izar[3], Elisabetta Visalberghi[5]

[1] Department of Science, Gorongosa National Park, Sofala, Mozambique

[2] CIBIO/InBIO, Centro de Investigação em Biodiversidade e Recursos Genéticos, Universidade do Porto, Campus de Vairão, Vairão, Portugal

[3] Institute of Psychology, Department of Experimental Psychology, University of São Paulo, Av. Prof. Mello Moraes 1721, CEP 05508-030, Cidade Universitária, São Paulo, Brazil

[4] Global Mammal Assessment Lab, Department of Biology and Biotechnology Charles Darwin, Sapienza University of Rome, Rome, Italy

[5] Istituto di Scienze e Tecnologie della Cognizione, Consiglio Nazionale delle Ricerche, Rome, Italy

[6] Primate Models for Behavioural Evolution Lab, Institute of Human Sciences, University of Oxford, UK

[7] Department of Life Sciences, University of Coimbra, Coimbra, Portugal

[8] School of Anthropology, Pontificia Universidad Católica de Chile, Santiago de Chile, Chile

*Corresponding author.

E-mail: susana.carvalho@gorongosa.net

¶These authors contributed equally to this work

ORCID

Noemi Spagnoletti: 0000-0002-8285-5963

Olivia Mendonça Furtado: 0000-0002-0643-5414

Joana Prietro: 0009-0000-8089-3416

Michele Verderane: 0000-0001-8467-8385

Elisabetta Visalberghi: 0000-0001-7407-5468

Susana Carvalho: 0000-0003-4542-3720

Dominique Cavallaro: 0009-0009-8667-7330

Megan Beardmore-Herd: 0009-0005-7681-6510

João M. P. Coelho: 0000-0002-4339-0550

Patrícia Izar: 0000-0001-6741-0731

Abstract

The consumption of small vertebrates occurs to varying degrees among many non-human primates and may have constituted an important source of nutrition for early hominins, particularly during periods of food scarcity, providing a stepping stone to the exploitation of larger animals. Capuchin monkeys regularly consume small vertebrates across their range. Expanding our knowledge of the contribution of animal matter to these primates' diets can shed light on the proximate and ultimate drivers of small vertebrate consumption across primate taxa and help evaluate hypotheses on the role of small prey exploitation in human evolution. We report on the most extensive record of bearded capuchin (*Sapajus libidinosus*) vertebrate predation so far available: 45 months of observations of vertebrate predation in two sympatric groups in Fazenda Boa Vista, Brazil, one of which was provisioned daily. We estimated the effect of provisioning, sex, age class, fruit and insect

availability, rainfall, humidity, and temperature on predation rates using a zero-inflated negative binomial algorithm with random effects. We found mammal and saurian consumption is habitual in this population of *S. libidinosus*, and prey brains and viscera tend to be accessed before the rest of the body. Predation rates were higher in males compared to females and in adults compared to infants across groups. Our results also identified small vertebrates as key energy sources for bearded capuchins under decreased fruit availability and show that provisioning reduces predation rates. Future work should assess small vertebrate predation as a possible stepping stone in the emergence of human exploitation of large animals by re-analyzing fossil faunal assemblages for evidence of small vertebrate predation and identifying the traces left by the exploitation of small prey in capuchins and other extant primates through neo-taphonomy.

Key words: capuchin behaviour, predatory patterns, foraging ecology, vertebrate consumption, availability of resources

Introduction

The habitual acquisition and consumption of animal matter is a shared behaviour among humans and non-human primates (hereafter primates) [1]. Despite this, humans are the only extant primate known to regularly exploit animals as large as, or larger than, themselves for energetic gains [2]. This strategy has been termed the "human predatory pattern" and is hypothesised to have originated from percussion-based scavenging of large animal carcasses to access inside-bone nutrients [3]. The increased energy content associated with this dietary shift may have enabled the evolution of larger brains, and the adaptive suite of defining human characteristics that came with

it [4–12]. Furthermore, in combination with cooperative hunting and technological advances, accessing animal resources would have acted as a buffer to seasonality in plant food availability [3,13].

Prior to the emergence of the human predatory pattern, small vertebrates (< 5 kg) may have constituted an important source of nutrition for hominins [14]. Being easier and less costly to catch than larger prey, as well as more widely distributed, the opportunistic exploitation of small animals could have been a crucial stepping stone, later evolving into the regular, tool-assisted exploitation of larger prey through passive or active scavenging and hunting. Support for this can be found in the zooarchaeological record, with faunal assemblages dating to around 2 million years ago providing direct evidence of sustained terrestrial and aquatic small animal resource exploitation by hominins with limited technological repertoires [15,16]. Furthermore, small game is a resource regularly accessed by several modern human hunter-gatherer populations, among them the !Kung of Botswana [17] and the Hadza of Tanzania [18]. There is also zooarchaeological evidence of the consumption of small vertebrates by Holocene North-American hunter-gatherers [19] as well as populations from the Patagonian Andes, in Argentina [20].

In the most recent review of meat eating by primates available, ingestion of vertebrate tissue was noted to occur in at least 89 species, and was most common amongst highly encephalized species like capuchin monkeys (*Cebus* and *Sapajus*), baboons (*Papio*), bonobos (*Pan paniscus*), and chimpanzees (*Pan troglodytes*) [1]. Across these primates, the prey classes most frequently targeted were small vertebrates, including birds, reptiles, amphibians, and mammals, although consumption rates varied between and within species [21]. Chimpanzees are reported to prey upon the widest range of vertebrate species [1,2], followed by bonobos [1]. Baboons also have broad prey

repertoires, which, for some species, include relatively large mammals such as goats, sheep, and dogs [22–24].

Capuchin monkeys are currently classified into four species of gracile capuchins (genus *Cebus*) and eight of robust capuchins (genus *Sapajus*) [25,26]. All members are highly opportunistic, generalist omnivores known for their dietary flexibility, their ability to prey upon fast-moving (and often stinging and biting) invertebrates and small vertebrates, and their extraction and processing of difficult-to-access food items using advanced cognitive and complex manipulative skills [27–32]. They also have high levels of behavioural flexibility, allowing them to adapt to distinct local conditions [33–35]. As a result of this, capuchin monkeys occupy a wide geographic distribution throughout Central and South America, from Honduras to northern Argentina [25,26], and cover a wide range of habitats, spanning from dry seasonal forests to semi-arid regions, mangroves, and tropical evergreen forests [29].

In some capuchin populations, vertebrate hunting and capture rates are within ranges similar to several chimpanzee populations [1] and vertebrate consumption may account for 2-3% of overall feeding time and up to 15% for some individuals during peak predation months [31,36]. The list of vertebrates that capuchin monkeys prey upon varies depending on site and species (Table 1), and continues to expand as available evidence grows. To date, this list includes mammals, such as squirrels (*Sciurus* sp.), coatis (*Nasua narica*), rodents (including the large rock cavie, *Kerodon rupestris*), juvenile anteaters (*Tamandua mexicana*) and bats; reptiles and amphibians, including snakes, lizards, frogs, and freshwater turtle eggs; and birds of several species [28,29,31,32,36–50]. Capuchin monkeys also capture and feed upon other primates. In Ilha de Germoplasma in eastern Brazil a tufted capuchin monkey (*Cebus apella*; *S. apella*) was seen to kill and feed upon an infant dusky titi monkey (*Plecturocebus moloch*) [51]. A group of the same capuchin

species was observed feeding on the carcass of an adult owl monkey (*Aotus brumbacki*) in what appeared to be a scavenging event in the Colombian Llanos [52]. Additionally, an adult male capuchin monkey (*Sapajus* sp.) in the Ibura National Forest of Brazil was reported to capture and presumably ingest a common marmoset (*Callithrix jacchus*), although predation was not observed directly [53].

Table 1. Vertebrates in the diet of capuchin monkeys.

Prey class	Prey type	Capuchin species	Site	Citations
Saurian *	Lizard	Bearded capuchin (*Sapajus libidinosus*)	Serra da Capivara National Park (Brazil)	[38]
		Golden-bellied capuchin (*Sapajus xanthosternos*)	Una Biological Reserve (Brazil)	[37]
		Blond capuchin (*Sapajus flavius*)	Caatinga dry forest area (Brazil)	[32]
			Reserva Particular do Patrimônio	[50]

			Natural Engenho Gargaú (Brazil)	
		White-faced capuchin (*Cebus capucinus*)	Santa Rosa National Park (Costa Rica)	[28,48]
			Lomas Barbudal (Costa Rica)	[48]
	Snake	Bearded capuchin (*Sapajus libidinosus*)	Serra da Capivara National Park (Brazil)	[38,39]
			Fazenda Boa Vista (Brazil)	[39]
	Turtle egg	White-fronted capuchin (*Cebus albifrons*)	Jaú National Park (Brasil)	[43]
	Iguana	White-faced	Santa Rosa	[48]

		capuchin (*Cebus capucinus*)	National Park (Costa Rica)	
	Adult bird	Bearded capuchin (*Sapajus libidinosus*)	Serra da Capivara National Park (Brazil)	[38]
		White-faced capuchin (*Cebus capucinus*)	Santa Rosa National Park (Costa Rica)	[28,31,48]
		Sapajus spp. (Captive hybrid)	Tietê Ecological Park (Brazil)	[40]
	Bird egg/nestling	White-faced capuchin (*Cebus capucinus*)	Santa Rosa National Park (Costa Rica)	[28,31,48]
			Lomas Barbudal (Costa Rica)	[48]
			Palo Verde	[48]

			(Costa Rica)	
		Sapajus spp. (Captive hybrid)	Tietê Ecological Park (Brazil)	[40]
Mammal	Rodent	Bearded capuchin (*Sapajus libidinosus*)	Serra da Capivara National Park (Brazil)	[38]
			Serra das Almas Private Reserve of Natural Heritage (Brazil)	[41]
		Azaras's capuchin (*Sapajus cay*)	Cabeceira do Prata Private Reserve (Brazil)	[44]
		Sapajus spp. (Captive hybrid)	Tietê Ecological Park (Brazil)	[47]
		White-faced	Santa Rosa	[31,48]

		capuchin (*Cebus capucinus*)	National Park (Costa Rica)	
			Lomas Barbudal (Costa Rica)	[48]
	Bat	Bearded capuchin (*Sapajus libidinosus*)	Serra da Capivara National Park (Brazil)	[38]
		White-faced capuchin (*Cebus capucinus*)	Santa Rosa National Park (Costa Rica)	[28,31,48]
			Lomas Barbudal (Costa Rica)	[48]
	Squirrel	White-faced capuchin (*Cebus capucinus*)	Santa Rosa National Park (Costa Rica)	[28,31]
			Lomas	[48]

			Barbudal (Costa Rica)	
			Palo Verde (Costa Rica)	[48]
		Black capuchin (*Sapajus nigritus*)	Santa Genebra (Brazil)	[42]
	Coati	White-faced capuchin (*Cebus capucinus*)	Santa Rosa National Park (Costa Rica)	[28,31,45,46]
			Lomas Barbudal (Costa Rica)	[48]
			Palo Verde (Costa Rica)	[48]
	Marsupial	*Sapajus* spp. (Captive hybrid)	Tietê Ecological Park (Brazil)	[47]
		Large-headed capuchin (*Sapajus*	Manu Learning Centre	[54]

		macrocephalus)	Biological Station (Peru)	
	Anteater (juvenile)	White-faced capuchin (*Cebus capucinus*)	Lomas Barbudal (Costa Rica)	[48]
	Primate	Brown or tufted capuchin (*Sapajus apella*)	Ilha de Germoplasma (Brazil)	[51]
			Arrayanes Farm (Colombia)	[52]
		Sapajus spp. (unidentified)	Ibura National Forest (Brazil)	[53]
Amphibian	Frog	White-faced capuchin (*Cebus capucinus*)	Santa Rosa National Park (Costa Rica)	[48]

*Clade composed of Lepidosauromorpha (snakes, lizards and rhynchocephalians) and Archosauromorpha (birds and crocodilians) [55].

The regular access to vertebrate prey of smaller and even similar size to their own (e.g., primates [52,53], rock cavy [41], anteater juveniles [48]) makes the capuchin dietary pattern an interesting model of the human predatory pattern [3] and the habitual small animal consumption that may have preceded its emergence [14]. In addition to commonly consuming vertebrates in the wild [28,31,38], capuchins exhibit a high degree of terrestriality (27% of their daily time is spent on the ground [56]), have complex food acquisition practices involving tool use and high levels of manual dexterity [57], and have the largest relative brain sizes of the platyrrhine primates [29]. Continuing to expand our knowledge of the contribution of animal matter to these primates' diets and the contexts in which vertebrate predation occurs in underrepresented wild populations allows us to shed light on the ultimate and proximate drivers of this behaviour, and reflect on the role of animal matter in human evolution.

Sex biases in vertebrate predation rates have been reported in some capuchin populations [28,31,38,48,58]. For example, at the long-term site of Santa Rosa National Park, Costa Rica, white-faced capuchin males have been observed attempting predation on more occasions than females, catching more vertebrate prey [28,48,58], and having higher predation success rates [31]. Similar results have been found in chimpanzees [59–61], and across the primate order more broadly [1,2]. This sexual bias has been hypothesized to be linked to sexual dimorphism in body mass demanding different caloric intake demands from males compared to females [62]. At Fazenda Boa Vista, the only field site where bearded capuchins (*S. libidinosus*) have been systematically weighed, the average body mass of adult subordinate male capuchins is 3.5 kg (range: 3.4–3.6 kg) while that of adult female capuchins is 2.1 kg (range: 1.8–2.6 kg) [63]. Thus, adult male capuchins may engage in more vertebrate consumption events than adult females to meet their greater energetic needs [58]. Furthermore, whilst reproduction and lactation increase

the energetic needs of females [64], they may buffer this demand by targeting reliable, low-risk food items, such as invertebrates [27], as opposed to vertebrate prey, the hunting which likely requires greater energy expenditure and results in unpredictable returns. This is supported by reports of female capuchins consuming more invertebrates than males on average [58].

There is less evidence on the effect of seasonality on vertebrate predation rates among capuchin monkeys. White-faced capuchin monkeys at Santa Rosa show increased vertebrate predation rates towards the dry season compared to the wet season, a period which coincides with some prey species' birthing season [28], as well as lower fruit availability and overall food abundance [31]. However, predation rates over some prey species, like birds, do not appear to follow seasonal trends [31].

In addition, capuchin monkeys appear to be highly socially tolerant in feeding and foraging activities [46,47,65]. Food transfer, where the original hunter does not monopolize the vertebrate prey and parts/all of it are passed to others either passively or actively [40], has been recorded in bearded capuchins from Serra da Capivara National Park [38] and Serra das Almas Private Reserve of Natural Heritage [41] in Brazil, as well as brown [40] and white-faced capuchins in other sites [31], and some chimpanzee populations [66,67]. Understanding the extent to which this behaviour is ubiquitous across capuchin populations requires information from more sites in these taxa's distribution.

Much of our understanding of capuchin monkey vertebrate consumption stems from studies of gracile capuchins (genus *Cebus*) [28,31,43,48]. Less focus has been given to vertebrate predation behaviour in robust capuchin monkeys (genus *Sapajus*), and, until recently, a large part of the available evidence on the subject was anecdotal and collected

across short periods of times [37,41,44,47,51–53]. However, recent field studies are beginning to overcome this bias. In a two-year-long study in the dry Caatinga habitat of Serra da Capivara National Park in Brazil, predation events by wild bearded capuchin monkeys (*Sapajus libidinosus*) were found to be relatively common with an overall rate of 4.6 events/100h [38], similar to those reported for the gracile white-faced capuchins of Santa Rosa, Costa Rica [28,31]. Furthermore, this Caatinga population is known to use probe tools to aid predation of small vertebrates, including lizards and snakes, followed by birds and rodents, but also larger prey, such as rock cavies (*Kerodon rupestris*), though only unsuccessful attempts have been observed for the latter [38,68].

With the aim of further expanding our understanding of the contribution of animal resources to robust capuchins' diet, we collected data on the vertebrate prey accessed by two sympatric groups of wild bearded capuchin monkeys (*S. libidinosus*; classified as Near Threatened by the IUCN Red List [73]) from Fazenda Boa Vista, north-eastern Brazil, (one of which was slightly provisioned daily during the dry season) across 45 months (May 2006–December 2010). Specifically, we described the type of prey captured, determined the rate of vertebrate predation in each group, and evaluated the effect of food provisioning, sex, age class and non-vertebrate food availability (fruit and insects) over predation rates. Additionally, to contribute to the understanding of whether this food transfer behaviours are ubiquitous in bearded wild capuchin populations, we also studied hunting and food transfer events in the Fazenda Boa Vista capuchins, noting the number and identity of individuals involved in each hunting event and in consuming the captured prey.

In particular, we tested the following hypotheses:

1. Sexual biases in predation rates:

1.1 Since different body mass among individuals of a given species demand different caloric intake [62,69], and the body mass of adult male capuchins is on average 60% larger than adult females [63], we predicted that adult male capuchins would engage in more vertebrate consumption events than adult females.

1.2 Alternatively, since greater energetic needs are placed on adult females compared to adult males by reproduction and lactation [64], adult females will engage in more vertebrate consumption events than adult males.

2. Effect of provisioning on predation rates:

The provisioned group has a higher net caloric intake than the unprovisioned group, as they have increased food availability and decreased food search costs. Therefore, the unprovisioned group will have greater need for vertebrate prey as a source of calories, and their vertebrate predation rates will be higher.

3. Age biases in predation rates:

3.1 Since the metabolism of juveniles expends more energy than the metabolism of adults, juvenile vertebrate predation rates will be higher than in adults.

3.2 Alternatively, due to the difficulties of hunting vertebrate prey, adults, who have more hunting experience, will show higher vertebrate predation rates than juveniles and infants, who have less hunting experience.

4. Temporal variability in alternative food availability:

4.1 Vertebrate prey replaces other high-protein food items that are less costly to obtain, such as insects, during periods of scarcity (e.g., during the dry season [70]). Therefore, vertebrate predation rates will increase during insect-poor periods.

4.2 Vertebrate prey replaces other high-calorie food sources that are less costly to obtain, like fruits, during periods of scarcity. Therefore, vertebrate predation rates will increase when fruit availability decreases.

5. Environmental effects over predation rates:

5.1 Environmental factors (rainfall, humidity and temperature) will not affect capuchin vertebrate predation rates.

5.2 Alternatively, environmental factors (rainfall, humidity and temperature) will have an effect on capuchin vertebrate predation rates.

6. Food transfer:

Given capuchin monkeys' social tolerance in feeding and foraging activities [46,47,65] and previous reports of food transfer events in bearded capuchins [38,41], food transfer events will be observed in the study population.

Materials and methods

Site

This study took place in Fazenda Boa Vista (hereafter, FBV; 9º 39' 36'' S, 45º 25' 10'' W), a privately owned area located in the northeastern Brazilian state of Piauí, 21 km northwest of the town of Gilbués. FBV is a transition zone between the Brazilian

biomes *Cerrado* and *Caatinga*. It consists of a sandy plain surrounded by 20-100 meter cliffs and interspersed by sandstone ridges and plateaus [71]. The majority of the plain is characterised by a high abundance of palms and medium-height trees, whereas the lowest part of the plain is a marsh conformed of gallery forest vegetation [71]. Meanwhile, the cliffs are dominated by shrubs and small trees, and the sandstone plateaus by low herbaceous vegetation [71]. The climate is characterized by marked rainfall seasonality which influences the availability of water in the ephemeral watercourses of the region and allows the distinction of two climatic seasons: a wet season from October to April and a dry season from May to September [70]. While the annual average rainfall in this area is 1156 mm, only an average of 230 mm of rain falls during the dry season [71].

Subjects

Our subjects belong to two wild and sympatric groups of bearded capuchin monkeys (*Sapajus libidinosus*), one of which was attracted to visit a study area by provisioning it daily with nuts, corn and water during the study period [70]. On average, each individual of the provisioned group received 825 kJ every day [70]. Both the unprovisioned group (composed by 8–16 individuals) and the provisioned group (composed by 17–19 individuals) have been continuously observed since 2005 as part of the Etho*Cebus* long-term research project on behaviour and ecology of wild bearded capuchin monkeys [72], and were thus well habituated to human presence.

Observations

Observations were carried out between May 2006 and December 2010 (Table 2). The overall observations of the unprovisioned group accounted for 3012 hours (distributed across 43 months across wet and dry seasons) and those of the provisioned

group accounted for 2812 hours (distributed across 45 months across wet and dry seasons) (Table 2). Data on vertebrate consumption events were collected following all occurrence sampling [74] and group scan sampling methods [75]. Since the sampling interval for collection of group scan activity budgets was different among observers, we divided the data collection into two study periods, S1 and S2, corresponding to the length of interval used.

In S1 (May 2006 to April 2008), we recorded data on activity budgets using a 10 min group scan sampling method at 10 min intervals, for a total of 84,470 scans (provisioned group: n=42,917; unprovisioned group: n=41,553). In S2 (April 2009 to December 2010), we recorded activity budgets using 1 min group scan sampling at 5 min intervals for a total of 75,028 scans (provisioned group: n=62,588; unprovisioned group: n=12,440). To test whether differences in sampling interval between study periods (S1 and S2) affected our results, we ran a generalized linear mixed model (GLMM) with a Poisson error structure. The response variable was the number of scans per month in which vertebrate consumption was observed, with study period (S1 vs S2) included as a fixed effect and total monthly scans as a covariate. Since we did not find a significant effect of study period in this model (χ^2=1.065, df=1, p=0.302), we considered all data collected by scan sampling during both study periods together in further analysis.

Table 2. Observation and demographic details for the two groups of capuchin monkeys studied at Fazenda Boa Vista: dates of observation periods, number of observation hours, study group (CH: Chicão, provisioned group; ZA: Zangado, unprovisioned group), group size, sex composition (♂: Males; ♀: Females), and age class composition (A: Adults, J: Juveniles, I: Infants).

Observation period[a]	Observation hours	Study group	Group size[b]	♂	♀	A	J	I
June 2006 - May 2007[1]	706	CH	12-18	11	7	11	5	2
	349	ZA	8-14	7	7	8	4	2
May 2006 - April 2008[2]	775	CH	17-19	13	8	12	4	5
	1932	ZA	8-14	7	8	9	4	2
Jan. 2009 - Dec. 2010[3]	1331	CH	17-19	11	10	9	8	5
	733	ZA	13-16	12	7	8	6	6

[a]Data sources: [1](81); [2](82); [3](83)

[b] Group size differed across observation periods due to births/deaths/migration.

A *Consumption Event* was defined as an individual observed taking and at least partially consuming any vertebrate item. We considered nestlings and eggs as vertebrate consumption [31]. We also scored which individual(s) captured the prey and which individual(s) ate it, and described which body parts were eaten. Hunting events were labelled Type I when one individual hunted the prey and Type II when more than one individual hunted the same prey simultaneously. Events in which food-transfer occurred were classified by referring to the individual(s) initially possessing the food item as *possessor(s)*, and the individual(s) to whom the transfer is directed as *recipient(s)* [40].

Data analysis: statistical modelling

We modelled the rate of predation events per unit of observation time as a function of the following fixed effect variables (model specified in eq. 1):

(1) Rainfall (mm). Recorded at the field station (Hygro-Thermometer Clock model 445702′, EXTECH instruments) daily.

(2) Temperature (ºC). Daily maximum and minimum temperatures were recorded at the field station (Hygro-Thermometer Clock model 445702′, EXTECH instruments).

(3) Humidity (%): Daily minimum and maximum recorded at the field station (Hygro-Thermometer Clock model 445702′, EXTECH instruments).

(4) Fruit availability. Estimated by combining:

 (a) Stratified random sampling method, whereby 100 fruit traps were placed at 30 m intervals, 1 m to the side of a 3 km trail crossing the different physiognomies of the study area. At two-week intervals, fallen material in the traps was quantified and weighed *in situ* and then returned to the same location, so fruits were not removed from their natural environment.. Food availability index (FAI; kg/ha) was estimated based on the dry weight of the material collected from traps and on the cumulative surface area.

 (b) Monthly direct observation and recording of presence/absence of fruit in 254 palm trees of the species catulé (*Attalea barreirensis*) (N = 136) and piassava (*Orbignya* sp.) (N = 118), in the same 3 km trail. This data was used to estimate two monthly FAI, one expressed as the number of palms with fruits/ha and one for total nuts, as the sum of catulé and piassava palms with fruits/ha.

(5) Insect availability. Estimated through stratified random sampling method, whereby 100 pitfall traps were placed at 30 m intervals, 1 m to the side of the same 3 km trail used to estimate fruit availability. At two-week intervals, fallen material in the traps was quantified and weighed in situ and then returned to the same location, so material was not removed from

its natural environment. Food availability index (FAI; kg/ha) was estimated based on the dry weight of the material collected from traps and on the cumulative surface area [70].

(6) Sex.

(7) Age.

(8) Group (unprovisioned vs. provisioned).

Correlations among predictor variables were assessed using Spearman's rank correlation coefficient (ρ) and variance inflation factors (VIFs) (S1 Table). Spearman's ρ indicated only low to moderate pairwise associations, and all VIF values were below 1.25, suggesting negligible multicollinearity. The strongest association was a moderate correlation between insect availability and rainfall (ρ=0.45). However, low VIF values indicated that the model was still able to distinguish between the seasonal increase in insect availability and the direct effect of rainfall on capuchin predation activity. In addition, model comparison based on Akaike Information Criterion (AIC) [76] values showed that models including both environmental variables (rainfall, temperature, and humidity) and food availability variables (fruit and insect availability) consistently outperformed models including environmental variables alone.

Our data set had many observations with 0 predation events documented. Since count data with many zero values cannot be transformed to normal [77], we implemented a predictive model capable of directly estimating the predation events observed. This predictive model also deals with excesses of zeros, and with non-linearity, non-homogeneity of variance and an outcome variable bounded at zero [78]. Excess zeros arise from low frequency of occurrences in behavioural studies and are thus influenced by an array of life-history processes and the ecological context. We can also have false zeros, that is when occurrences are happening, but not during the surveying period, or

when occurrences happen during surveying but are not detected [79,80]. When working with longitudinal data (characterized by repeated measurements from individuals within and across units of time), it is necessary to explicitly model the random effects structure. This allows for a correct inference of the fixed effects, accounting for non-independence among observations and avoiding pseudoreplication [81,82]. Thus, we relied on zero-inflated generalized linear mixed models (ziglmm) to perform the analysis, more specifically a zero-inflated negative binomial (zinb) algorithm with crossed random effects. To standardize for observation effort, we included the log of the number of scan samples as an offset term (*logtime*), so that the model estimates predation rates per unit observation effort rather than raw counts. Therefore, our model was defined as follows:

$$\begin{aligned}&Predation\ Events \sim offset(\log time) + Sex + Group + Rainfall + Fruit + Insect + (1|ID) + (1|Month)\\&Zero\ Inflation \sim Humidity + Temperature\end{aligned}$$

(eq. 1)

where the random effects of individual ID and month (1-45) are expressed in parentheses.

The negative binomial distribution is technically Poisson-distributed with underlying gamma-distributed heterogeneity [83]. So, it is better suited than a typical Poisson regression to account for overdispersion [84]. All parameters were fitted in R with the glmmTMB package, using maximum likelihood estimation, except for random effects which were estimated via a Laplace approximation [85–87]. After running many different model architectures, we used information-theory for multi-model inference to select the final reduced model. Notice that some architectures could not be modelled because of convergence problems while estimating parameters (e.g., non-positive-definite Hessian matrix; extreme or very small eigenvalues detected; and/or singular convergence). We

selected as the final model (eq. 1) the one with the lowest Akaike Information Criterion (AIC) value, as this metric optimises the trade-off between fit and complexity [76,82,88].

The specified model (eq. 1) was applied to all collected data on vertebrate predation (Model 1), as well as two subsets of it: predation events involving mammal prey only (Model 2) and predation events involving non-mammal prey only (Model 3). We refer to non-mammalian prey as Sauria, a clade composed of Lepidosauromorpha (snakes, lizards and rhynchocephalians) and Archosauromorpha (birds and crocodilians) [55]. Models 2 and 3 were applied to compare patterns of mammal vs. saurian predation in the studied populations.

Additionally, we also used Non-parametric tests (Chi-square tests) to assess whether hunting events were more frequently performed by one (Type I) or more individuals (Type II) and whether consumption was more frequently performed by one or more individuals. Statistical significance level was set at α = 0.05 for all of the analyses performed.

Ethical note

The research adhered to the Brazilian government permit (SisBio #28689) in accordance with the Brazilian legislation (law #11.794, October 8, 2008) and to the Brazilian National Council for Scientific and Technological Development (CNPq) authorization for Scientific expedition, under the Project CMC 000111, which included the permission from the landowners Marino Gomes de Oliveira and Maria Fonseca Gomes de Oliveira to conduct research in their field site. We adhered to the American Society of Primatologists' principles for the ethical treatment of primates.

Inclusivity in global research

Additional information regarding the ethical, cultural, and scientific considerations specific to inclusivity in global research is included in the Supporting Information (S1 Checklist).

Results

Each group of capuchins was followed from dawn to dusk. During a total of 5,093 observation hours we obtained a total of 446 events of vertebrate consumption (both when in progress or about to begin). Of the 446 events, 281 were recorded by scan sampling and 165 were recorded by all occurrence sampling. The provisioned group accounted for 212 events, and the unprovisioned group for 234 events. We recorded 8 events of vertebrate consumption by infants (3 lizards, 2 rodents, 2 snakes, 1 undetermined prey). For the modelling analyses only the scan sampling events were used. We obtained an overall rate of 6.34 predation events/100h.

Consumed prey items

Similarly to what has been observed elsewhere [40,44,47], the most common prey of capuchins in FBV are vertebrates of small dimensions like lizards and rodents. The major prey types were diurnal and abundant (pers. com.) reptiles (n=136: lizards, geckos, snakes, and iguanas), mammals (n=97: primarily rodents, but also opossums, and two bats) and birds (n=34: especially nestlings and eggs) (Table 3; see S1 Video).

Table 3. Number of prey items consumed in the 281 events of vertebrate consumption recorded by scan sampling in the provisioned and unprovisioned groups. Birds include both nestling and eggs. Und. = Undetermined.

Mammals			Saurian				Und.
Rodents	Marsupials	Bats	Lizards	Birds	Snakes	Iguana	
80	15	2	123	34	12	1	14
28.5%	5.3%	0.7%	43.8%	12.1%	4.3%	0.4%	5.0%

Avian prey were usually eaten (Fig. 1A). When this was not the case, the monkeys preferred to eat the brain and viscera. For mammalian prey the monkeys preferred to eat the tail and paws, and in some events the eyes as well. The skin covering the forehead and the back of the prey was ripped off in a few cases (Fig. 1B). For geckos and lizards, capuchins frequently ate feet, tail, eyes, brain, skin and, in some cases, viscera (Fig. 1C). Only male capuchin monkeys were observed consuming snakes. When eating snakes (Fig. 1D), they often discarded the head and ate the rest of the body, especially the internal organs. In general, muscular tissue and associated ligaments and tendons were torn apart in small bites, repeatedly masticated and swallowed; tender parts of the prey, such as the viscera and brain, were ingested more rapidly. We never observed meat consumption accompanied by leaf ingestion.

Due to limited numbers of observations in certain categories (e.g., 2 bats, 12 snakes, etc.), we concatenated small prey subsets, enabling statistical analyses and modelling. For example, alongside a global model, we compared mammals versus saurians using two distinct sub-models.

Fig. 1. Evidence of vertebrate consumption at FBV. A (top left): Adult male feeding on avian prey (photo by Luciano Candisani); B (top right): Mammal prey (marsupial) preyed upon by an adult male. The skin was ripped off and the viscera exposed (photo by Noemi Spagnoletti); C (bottom left): Lizard preyed upon by an adult female. Viscera were exposed (photo by Eduardo D. R. Silva); D (bottom right): Snake preyed upon by an unidentified individual. The upper part of the snake containing the head was ripped off and the viscera exposed (photo by Noemi Spagnoletti).

Model 1: Global model for vertebrate consumption

Individuals in the provisioned group consumed significantly less vertebrate prey than those in the unprovisioned group (ZA; $p < 0.001$; Table 4; Fig. 2). The estimated rate of predation events per unit of observation time in the non-provisioned group was 1.88 times higher than in the provisioned group. Infants consumed vertebrate prey at only one-fifth the rate of adults ($p < 0.002$), whereas juveniles did not differ significantly from adults (Fig. 3). Males showed higher rates of vertebrate consumption than females, with an estimated rate ratio of 1.4 ($p = 0.049$).

Table 4. Results of the zero-inflated negative binomial generalized linear mixed model testing predictors of vertebrate predation rate per unit of observation time.

**Term**	**Estimate**	**SE**	**Lower CL**	**Upper CL**	**Wald's**	***p*-value**
Conditional model (Negative Binomial)						
(Intercept)	-6.6802	0.199334	-7.07089	-6.28951	-33.5126	-
Sex (Male)	0.348003	0.176809	0.001463	0.694543	1.968241	0.04904*

Age (Juvenile)	-0.17863	0.174004	-0.51967	0.162411	-1.02659	0.304616
Age (Infant)	-1.48032	0.468656	-2.39886	-0.56177	-3.15864	0.001585*
Group (ZA)	0.632806	0.183204	0.273734	0.991879	3.454114	0.000552*
Rainfall	0.428193	0.169339	0.096294	0.760092	2.52861	0.011452*
Fruits	-0.49586	0.146094	-0.7822	-0.20952	-3.39411	0.000689*
Insects	-0.23761	0.14441	-0.52065	0.045427	-1.64539	0.099889
Zero Inflation model						
(Intercept)	25.13092	16.93205	-8.05529	58.31714	1.484222	-
Temperature	-2.11166	0.858397	-3.79409	-0.42924	-2.46001	0.013893*
Humidity	0.377545	0.130974	0.120841	0.634249	2.882603	0.003944*

Parameter estimates are shown for the conditional and zero-inflation components. Observation effort was accounted for by including log(number of scan samples) as an offset term. n = 1092. Random effects: ID (36); Month (45). Final model metrics: AIC = 1253.7; BIC = 1323.6; logLik = -612.8; deviance = 1225.7; df residuals = 1078. ZA: unprovisioned group. Asterisks indicate $p < 0.05$. Reference categories: Sex: female; Age: adult; Group: provisioned.

Fig. 2. Dot-whisker plot showing the incidence rate estimates for predation events based on all variables in the model.

Fig. 3. The likelihood of occurrence of a predation event by group (CH: provisioned; ZA: unprovisioned), age class, and sex.

Rainfall positively affected the rate of predation events per unit observation time (p = 0.011; Fig. 4). In contrast, fruit availability showed a strong negative association with predation rates ($p < 0.001$; Fig. 5). Insect availability was not significantly associated with predation rate (p = 0.1). Temperature (P = 0.014) and humidity (P = 0.004) were used in a separate part of the model—the zero-inflation model. Temperature had a significant negative effect (p = 0.014): higher temperatures reduced the probability that an observation fell into the "always zero" state, making predation events more likely to be recorded. In contrast, humidity had a significant positive effect (p = 0.004): higher humidity increased the probability of an excess zero, i.e., conditions under which no predation events were observed.

Fig. 4. Effect of rainfall (mm) on the likelihood of a capuchin monkey preying on a vertebrate. Adjusted for variables kept fixed as logtime = 4.74; Age Class = Adult; Fruit availability = 289.89; Insect availability = 0; Temperature = 0, Humidity = 0.

Fig. 5. Effect of fruit availability (kg/ha) on capuchin vertebrate predation rate. Adjusted for variables kept fixed as logtime = 4.74; Age Class = Adult; Rainfall = 94.99; Insect availability = 0; Temperature = 0, Humidity = 0.

Model 2: Sub-model of mammal consumption

The primary focus of this analysis was to model predation events involving mammals, namely rodents, marsupials, and bats (S2 Table; Fig. 6A). We again employed a negative binomial mixed-effects model with a zero-inflation component to address overdispersion and excess zeros in the data, respectively. The model exhibited good fit statistics, with an AIC of 502.6, and several predictors showed significant associations with variation in mammalian predation rates.

Males showed significantly higher mammal predation rates than females ($p = 0.006$) (S2 Table). Age was not a statistically significant predictor of mammal predation rate. The difference between the provisioned and unprovisioned groups was also not significant. Increased rainfall ($p = 0.029$) and higher fruit availability ($p < 0.001$) were both significantly associated with higher and lower predation rates, respectively. Temperature significantly reduced the probability of excess zeros ($p = 0.021$), indicating that predation events were more likely to be observed at higher temperatures, while humidity showed a non-significant trend.

Model 3: Sub-model of saurian predation

The same zero-inflated negative binomial regression model was employed to investigate the relationship between saurian predation events and group, individual and environment-related predictors (S3 Table; Fig. 6B). Saurian predation showed a significant positive association with group ($p = 0.004$), with the unprovisioned group showing a higher saurian predation rate than the provisioned one. We did not find significant relationships between any environmental variable and predation rate. We also did not find a significant effect of sex or age over predation rate. Overall, our results suggest that saurian predation events are most strongly influenced by provisioning.

Fig. 6. Contrasting sub-models with predation rate split by prey type: A. mammals; B. saurians. ZA: unprovisioned group.

Hunting and food transfer events

Hunting events were rarely observed. Out of 280 consumption events recorded by scan sampling for both groups, we observed hunting preceding consumption in 51 instances (see S2 Video). In all other cases, hunting was noticed as the prey was grasped, or inferred to have occurred from the presence of prey seen in a capuchin's hand (see S3 Video). Consumption of vertebrate prey was significantly higher in type I events (individual, 82.14%, N = 230) than in type II events (collective, 17.86% N = 50) Chi-square, χ^2= 17,708, df=2, p=0.000). Of the 165 vertebrate predation episodes recorded by all occurrence sampling for both groups, 81.21% (n=134) were episodes in which only one individual was seen consuming the prey item, whereas 18.87% (n=31) were episodes in which two or more individuals were seen consuming the same prey item. The transfer of meat, or of other parts of the carcass (such as skin, bones, etc.) was observed in 32 of the 50 type II events (64%).

Discussion

We reported on the most extensive record of bearded capuchin (*S. libidinosus*) vertebrate predation currently available: 45 months of vertebrate consumption observations across two sympatric groups in Fazenda Boa Vista (Brazil). We showed that vertebrate consumption is habitual in these groups (*sensu* McGrew, 1992 [89]) and occurs at an average rate of 6.34 predation events per 100 hours. This behaviour was seen across

all age classes (adults, juveniles and infants) and both sexes. Like bearded capuchins in other areas of Brazil (e.g., Serra da Capivara National Park [38]; Serra das Almas Private Reserve of Natural Heritage [41], and in support of other observations of the Fazenda Boa Vista capuchins [39]), both groups studied here were observed feed on rodents, bats, lizards, adult birds and snakes. For the first time in bearded capuchins, we also recorded opossums, iguanas and bird eggs/nestlings as part of this species' diet. Additionally, we observed both hunting events with more than one participating individual, as well as food transference events.

Our results revealed a strong association between food availability and the exploitation of small animal resources (see also Table 5 below). First, provisioned individuals exhibited lower vertebrate predation rates than unprovisioned ones. This effect was upheld when looking only at saurian prey, but not when looking only at mammalian prey. Provisioning has been shown to alter primates' activity budgets, diets and ranging patterns by providing resources of a higher caloric content, availability and predictability than those found in their natural habitat [90,91]. Asquith (1989) [92] raised concerns about the lack of comparative data between provisioned and unprovisioned individuals in primate studies. Our research directly addresses this issue by using a quasi-experimental approach comparing the predation patterns of provisioned and unprovisioned groups of sympatric capuchins. Future studies on wild primate dietary patterns must strongly consider the potential influence of planned or unplanned (e.g., crop raiding, feeding on garbage) provisioning on predatory behaviours, as our results suggest primates may be less inclined to access animal foods when provisioned.

Table 5. Factors considered in this study, their prediction(s) in terms of vertebrate consumption, and whether results support each of them.

**Category**	**Predictions on vertebrate predation rate (VPR)**	**Prediction supported?**
1. Sexual biases in predation rates.	1.1 VPR in adult males > adult females.	Yes.
	1.2 VPR in adult males < adult females.	No.
2. Effect of provisioning on predation rates.	VPR in unprovisioned group > provisioned group.	Yes.
3. Age biases in predation rates.	3.1 VPR in juveniles > adults.	No.
	3.2 VPR in juveniles and infants < adults.	No for juveniles.<br>Yes for infants.
4. Temporal variability in alternative food availability.	4.1 Insect availability is negatively correlated with VPR.	No.

	4.2 Fruit availability is negatively correlated with VPR.	Yes.
5. Environmental effects over predation rates.	5.1 Environmental factors (rainfall, humidity and temperature) will not have an effect on VPR.	No.
	5.2 Environmental factors (rainfall, humidity and temperature) will affect VPR.	Yes.
6. Food transfer.	Food transfer events will be observed.	Yes.

Second, we found vertebrate meat intake varies inversely with fruit abundance, a trend upheld in the mammal-only model but not in the saurian-only model. This suggests small mammals may be important sources of energy in wild bearded capuchins at Fazenda Boa Vista, and may replace other sources of calories (i.e., fruits) during periods of scarcity. This aligns with the “energy shortfall hypothesis”, which posits that animal-source foods are a particularly important source of energy for primates when plant-source foods become less available [1]. A similar mechanism has been proposed as a driver of increased meat consumption in the genus *Homo* [13]: as late Pliocene and early Pleistocene eastern African hominin environments became increasingly seasonally arid [13,58,93], the availability of plant-source foods that are key to many primates’ diet in

stable, humid, forested habitats would have only been available intermittently, whereas animal resources (flesh and in-bone nutrients like marrow and brain) would have remained accessible throughout the year, thus selecting for a higher frequency of predatory behaviours in our lineage [13]. Bearded capuchin reliance on small vertebrates during fruit-poor periods supports this strategy as a possible predecessor and/or complement to large-prey hunting in humans in seasonal environments, possibly along with opportunistic scavenging of within-bone nutrients [3]. Evidence from the fossil and archeological record [15,16,19,20] and contemporary forager societies [17,18] indicates that such small-vertebrate exploitation has had a lasting presence in the human lineage.

However, the population of Fazenda Boa Vista appears to be an exception among extant primates: the most extensive review of animal predation across the primate order to date found little evidence in support of the energy shortfall hypothesis [1], even in species inhabiting seasonally extreme environments similar to those reconstructed for early Pleistocene hominins, like baboons [1] and savanna chimpanzees [1,94]. For instance, the hunting behaviours of chimpanzees in the savanna-woodlands of Fongoli are not explained by fruit scarcity [94]. Furthermore, chimpanzees in some forested environments appear to hunt more often during food-rich rather than food-poor seasons, possibly because they can afford the energetic losses of an unsuccessful hunt when they have another easily obtainable source of energy: fruit [1,59,95]. White-faced capuchins in Santa Rosa (Costa Rica) do consume more vertebrate prey in the dry season, when fruit is scarce, but this is likely due to the concurrent increase in preferred prey availability, as coati infants are born at this time [1,31]. Future research should aim to clarify whether the association between fruit abundance and vertebrate consumption in the Fazenda Boa Vista capuchins truly supports the energy shortfall hypothesis, or if the observed pattern is a result of opportunistic predation of seasonally abundant small mammals.

It is important to note that, in this study, we combined the availability of palm nuts with that of other fruits. The kernels of palm nuts constitute a key food item for the capuchins at Fazenda Boa Vista: they are rich in lipids and protein, and provide a reliable source of energy and a stable protein intake [96]. It is therefore possible that the observed inverse relationship between vertebrate predation and fruit availability is strongly influenced by fluctuations in nut consumption and availability. This requires further testing. Additionally, it is possible that the measure of fruit availability used in the present study does not reflect variation in capuchin fruit consumption (e.g., capuchins may consistently access some fruits regardless of their availability, or they may not increase their consumption of non-preferred fruits despite their increased availability). Therefore, future studies should aim to directly quantify temporal variations in fruit consumption in capuchins and test their effect on vertebrate consumption rates.

Environmental factors also had a significant effect over capuchin vertebrate consumption patterns. Increased rainfall was linked to increased vertebrate consumption rates in this population. Furthermore, predation events were more likely to be recorded at higher temperatures and less likely to be recorded at higher humidity levels. Rather than reflecting a direct effect of these environmental variables over vertebrate predation, our results likely reflect the presence of a mediating variable: the availability of preferred vertebrate prey species. It is possible that periods of higher temperatures and rainfall, as well as lower humidity, coincide with the birthing season of some prey species. These seasons have been linked with increased vertebrate predation rates in other capuchin populations, as they are characterized by more readily abundant and easier-to-catch prey: infants [31]. Future work should aim to disentangle these effects by directly measuring the effects of vertebrate prey abundance over capuchin vertebrate predation rates.

We also found strong effects of age and sex on capuchin vertebrate consumption rates. Across both study groups, infants–but not juveniles–had lower vertebrate consumption rates than adults, and males had higher predation rates than females. These sex differences are similar to those documented at other long-term capuchin monkey field sites [28,31,38,40,58], as well as in baboons [97–102] and chimpanzees [1]. Improving our understanding of these patterns in robust capuchins allows us insights beyond those provided by chimpanzees, baboons and gracile capuchins, on whom much of the research on the subject has focused. That males appear to capture more vertebrates than females across all the aforementioned primate taxa presents an interesting pattern, especially considering ongoing debates about sex biases in hunting among modern unindustrialized societies [103,104]. Across many of these communities, men hunt more often than women on average [104–107]. Although there is variation between societies and women regularly hunt in many [103,108–111] these sex differences appear to be present and accurately reported [104].

Aside from increased caloric intake demanded by male bearded capuchin's body size compared to that of females [58,62], the observed sex differences in predation rates may be explained by a risk-averse foraging strategy in females compared to males [61]. For instance, all snake predation events were performed by males in our study population, which may reflect female avoidance of potentially dangerous prey. In chimpanzees, females feed significantly more on insects, such as termites and ants, than males [112,113]. This has been proposed as a strategy that combines longer investment of time to access high quantities of protein and calories from sources (A) more reliable and less energetically costly than vertebrate hunting, which has an important percentage of failure, (B) under less competition, as invertebrates are more widely available than vertebrates and males seek them less, and (C) that pose a lower risk of injury to females and infants,

as well as of losing prey to males, than hunting [112–114]. Gilby *et al*., (2017) [112] posited this strategy might have been present in the last common ancestor between the *Pan* lineage, to which chimpanzees belong, and the human lineage, hypothesizing sex differences in hunting behaviour would have preceded the appearance of the sexual division of labor among modern humans. In unindustrialized societies today, female risk aversion has also been posited as a driver of the sexual division of labor [115]. By extending the comparison beyond chimpanzees and humans, capuchins may provide insights on the extent to which whether sex biases in vertebrate predation may be a general response to the risks and energetic trade-offs of hunting. Like chimpanzees, female white-faced capuchins tend to consume more invertebrates than males [58] and insects are a vital source of energy for them, particularly during lactation [27]. We did not evaluate differences in invertebrate predation rate between bearded capuchin males and females; future research should aim to bridge this gap. Convergent sex-biases in invertebrate and vertebrate access across distantly related primate taxa might suggest that female avoidance of high-risk prey may be a recurrent strategy among vertebrate-eating primates.

The majority of the hunting events we observed, as well as the consumption of captured prey, were carried out by one individual rather than by two or more. Instances of prey transfer did occur in our population, as in the bearded capuchins from Serra da Capivara National Park [38] and Serra das Almas Private Reserve of Natural Heritage [41]. However, many of the small vertebrates captured by a group of hunters were monopolized and consumed by only one individual. Factors such as dominance rank may influence who gains priority access to prey, as subordinate individuals may be supplanted [31], while kinship ties could play a role in determining whether prey is shared [116]. Nevertheless, most predation events were performed alone and in the absence of other group members, suggesting that collaborative hunting is not necessary for the capture of

small prey in bearded capuchins. Instead, solitary hunting and eating may minimize intraspecific competition for limited food. Many small vertebrates may not provide enough nutrients, proteins and energy for more than one individual, potentially discouraging cooperative hunting and sharing. For example, larger prey like adult squirrels appeared to be shared by white-faced capuchins in Santa Rosa, whereas nestlings were usually entirely eaten by one captor [31]. Similarly, smaller prey sizes have been reported to discourage cooperative hunting in chimpanzees [117]. Future research might look into whether prey size may be a predictor of the likelihood of group hunting and sharing in bearded capuchins.

Capuchin monkeys in the present study consumed vertebrate's brains and viscera first, and then (but not always) the rest of the body. Capuchins' preferential access of these tissues may respond to their high lipid (and protein, in the case of viscera like liver) content compared to other prey bodily tissues [118,119]. For example, chimpanzees at Gombe National Park consistently consume the brains of subadult prey and the viscera of adult prey, whose skulls are more robust and harder to crack, before moving to the rest of their body, possibly to maximize the fats and nutrients obtained before the carcass is stolen by conspecifics [120,121]. A similar consumption order has been observed in Taï [66] and Fongoli [122] chimpanzees. The scavenging of in-bone nutrients, such as those provided by the brain, have been posited as one of the stepping-stones towards the emergence of the human predatory pattern [3]. Like bearded capuchins and chimpanzees, hominins may also have preferentially accessed the brains of small prey, and later began scavenging these in-bone nutrients from large carcasses, as proposed by Thompson *et al.*, (2019) [3], before they were able to hunt prey larger than themselves. However, our results differ from those of [36] on the white-faced capuchins of Santa Rosa, Costa Rica, who appear to consume the viscera first and typically discard the head. Further research on the

sequence in which different primates consume prey body parts will provide a more nuanced understanding of the factors shaping their contrasting prey-consumption strategies [120].

While primates accessing prey of the same size or larger than themselves is only seen in humans [3], the consumption of small vertebrates is seen across a large number of taxa [1]. On one hand, this may reflect an ancient opportunistic strategy for the obtention of energy, nutrients and/or proteins. On the other hand, small vertebrate predation may have emerged through convergent evolution in several branches of the primate order, as multiple species recurred to the same solution to address similar ecological challenges. Hunting small prey does not necessarily require tools or cooperation with group members to be successful, which may contribute to the persistent presence of this behaviour in the primate order [1], including in hominins [14,15,17,19,20]. Despite the emergence of the human predatory pattern, the consumption of small vertebrates has persisted in the human lineage, and small game forms part of the diet of many unindustrialized societies today [16,18]. The presence of this form of vertebrate consumption across primate taxa supports the possibility that small prey provides an important, reliable and less costly source of nutrients than larger prey, the capture of which has a very high rate of failure and requires larger energetic investments.

Future directions

We recommend that early faunal assemblages composed of small vertebrates should be re-examined closely for evidence of damage inflicted by predation. Future capuchin monkey studies investigating vertebrate consumption should record the order in which different parts of the animal are consumed and document the taphonomy of these processes, so that we may identify the traces that small vertebrate predation leaves behind

after the animal is consumed and abandoned, and thus build a better picture of the signatures we should be searching for in the fossil record. This will allow us to identify how patterns of consumption have changed through time in assemblages with hominins and other fossil primate presence.

Acknowledgements

We thank the Fonseca de Oliveira family for permission to work on their land, as well as the Etho*Cebus* Project team, supporting the logistics of the research. We are also grateful to our field assistants, Jozemar de Oliveira, Arisomar de Oliveira, Renato de Olivera, Marino Júnior and Marcos Fonseca de Oliveira.

References

1. Watts DP. Meat eating by nonhuman primates: A review and synthesis. J Hum Evol. 2020;149: 102882. doi:10.1016/j.jhevol.2020.102882

2. Butynski TM. Vertebrate predation by primates: a review of hunting patterns and prey. J Hum Evol. 1982;11: 421–430. doi:10.1016/S0047-2484(82)80095-X

3. Thompson JC, Carvalho S, Marean CW, Alemseged Z. Origins of the Human Predatory Pattern: The Transition to Large-Animal Exploitation by Early Hominins. Curr Anthropol. 2019;60: 1–23. doi:10.1086/701477

4. Aiello LC, Wheeler P. The Expensive-Tissue Hypothesis: The Brain and the Digestive System in Human and Primate Evolution. Curr Anthropol. 1995;36: 199–221. Available: https://www.jstor.org/stable/2744104

5. Aiello LC, Bates N, Joffe T. In defense of the Expensive Tissue Hypothesis. In: Falk D, Gibson KR, editors. Evolutionary Anatomy of the Primate Cerebral Cortex. Cambridge: Cambridge University Press; 2001. pp. 57–78. doi:10.1017/CBO9780511897085.006

6. Antón SC, Potts R, Aiello LC. Evolution of early Homo: An integrated biological perspective. Science. 2014;345: 1236828. doi:10.1126/science.1236828

7. Foley R. The Evolutionary Consequences of Increased Carnivory in Hominids. In: Stanford CB, Bunn HT, editors. Meat-Eating & Human Evolution. Oxford University Press; 2001. p. 0. doi:10.1093/oso/9780195131390.003.0016

8. Hagen EH, Blackwell AD, Lightner AD, Sullivan RJ. Homo medicus: The transition to meat eating increased pathogen pressure and the use of pharmacological plants in Homo. Am J Biol Anthropol. 2023;180: 589–617. doi:10.1002/ajpa.24718

9. Kaplan H, Hill K, Lancaster J, Hurtado AM. A theory of human life history evolution: Diet, intelligence, and longevity. Evol Anthropol Issues News Rev. 2000;9: 156–185. doi:10.1002/1520-6505(2000)9:4%3C156::AID-EVAN5%3E3.0.CO;2-7

10. Leonard WR, Snodgrass JJ, Robertson ML. Effects of Brain Evolution on Human Nutrition and Metabolism. Annu Rev Nutr. 2007;27: 311–327. doi:10.1146/annurev.nutr.27.061406.093659

11. Pontzer H. Ecological Energetics in Early Homo. Curr Anthropol. 2012;53: S346–S358. doi:10.1086/667402

12. Roebroeks W. Guts and Brains: An Integrative Approach to the Hominin Record. Leiden University Press; 2007. doi:10.24415/9789087280147

13. Foley R. Another unique species: patterns in human evolutionary ecology. Harlow: Longman Scientific & Technical; 1987.

14. Braun DR. Pliocene Hominin Behavior: New Insights. Curr Anthropol. 2019;60: 10–12. doi:10.1086/701477

15. Archer W, Braun DR, Harris JWK, McCoy JT, Richmond BG. Early Pleistocene aquatic resource use in the Turkana Basin. J Hum Evol. 2014;77: 74–87. doi:10.1016/j.jhevol.2014.02.012

16. Ferraro JV, Plummer TW, Pobiner BL, Oliver JS, Bishop LC, Braun DR, et al. Earliest Archaeological Evidence of Persistent Hominin Carnivory. PLoS ONE. 2013;8: e62174. doi:10.1371/journal.pone.0062174

17. Lee RB. The !Kung San: Men, Women and Work in a Foraging Society. CUP Archive; 1979.

18. Hawkes K, O'Connell JF, Blurton Jones NG. More Lessons from the Hadza about Men's Work. Hum Nat. 2014;25: 596–619. doi:10.1007/s12110-014-9212-5

19. Reinhard KJ, Ambler JR, Szuter CR. Hunter-gatherer use of small animal food resources: coprolite evidence. Int J Osteoarchaeol. 2007;17: 416–428. doi:10.1002/oa.883

20. Andrade A, Fernández PM. Rodent consumption by hunter-gatherers in north Patagonian Andean forests (Argentina): Insights from the small vertebrate

taphonomic analysis of two late Holocene archaeological sites. J Archaeol Sci Rep. 2017;11: 390–399. doi:10.1016/j.jasrep.2016.12.004

21. Hosaka K, Nakamura M, Takahata Y. Longitudinal changes in the targets of chimpanzee (Pan troglodytes) hunts at Mahale Mountains National Park: how and why did they begin to intensively hunt red colobus (Piliocolobus rufomitratus) in the 1980s? Primates. 2020;61: 391–401. doi:10.1007/s10329-020-00803-8

22. Holmern T, Nyahongo J, Røskaft E. Livestock loss caused by predators outside the Serengeti National Park, Tanzania. Biol Conserv. 2007;135: 518–526. doi:10.1016/j.biocon.2006.10.049

23. Mosissa T, Ameha A, Mosissa T. Assessment of the abundance of Anubis baboons (Papio anubis) and conflict with humans in Trigni Forest of Gida Ayana District, Western Ethiopia. J Ecol Nat Environ. 2017;9: 1–14. doi:10.5897/JENE2017.0622

24. Sogbohossou EA, Iongh HH de, Sinsin B, Snoo GR de, Funston PJ. Human–carnivore conflict around Pendjari Biosphere Reserve, northern Benin. Oryx. 2011;45: 569–578. doi:10.1017/S0030605310001109

25. Alfaro JWL, Silva JDESE, Rylands AB. How different are robust and gracile capuchin monkeys? An argument for the use of sapajus and cebus. Am J Primatol. 2012;74: 273–286. doi:10.1002/ajp.22007

26. Martins-Junior AMG, Carneiro J, Sampaio I, Ferrari SF, Schneider H. Phylogenetic relationships among Capuchin (Cebidae, Platyrrhini) lineages: An old event of sympatry explains the current distribution of *Cebus* and *Sapajus*. Genet Mol Biol. 2018;41: 699–712. doi:https://doi.org/10.1590/1678-4685-GMB-2017-0012

27. Bergstrom ML, Hogan JD, Melin AD, Fedigan LM. The nutritional importance of invertebrates to female Cebus capucinus imitator in a highly seasonal tropical dry forest. Am J Phys Anthropol. 2019;170: 207–216. doi:10.1002/ajpa.23913

28. Fedigan LM. Vertebrate Predation in Cebus capucinus: Meat Eating in a Neotropical Monkey. Folia Primatol (Basel). 1990;54: 196–205. doi:10.1159/000156444

29. Fragaszy DM, Visalberghi E, Fedigan LM. The Complete Capuchin: The Biology of the Genus Cebus. Cambridge University Press; 2004.

30. Parker ST, Gibson KR. Object manipulation, tool use and sensorimotor intelligence as feeding adaptations in cebus monkeys and great apes. J Hum Evol. 1977;6: 623–641. doi:10.1016/S0047-2484(77)80135-8

31. Rose LM. Vertebrate Predation and Food-Sharing in Cebus and Pan. Int J Primatol. 1997;18: 727–765. doi:10.1023/A:1026343812980

32. Souza-Alves JP, Arruda de Almeida H, Rufino MG. Animal protein intake by blonde capuchin monkeys in a Brazilian semi-arid environment. J Nat Hist. 2026;60: 97–103. doi:10.1080/00222933.2025.2598134

33. Kinzey WG. New World Primates: Ecology, Evolution, and Behavior. Aldine de Gruyter; 1997.

34. Izar P, Verderane MP, Peternelli-dos-Santos L, Mendonça-Furtado O, Presotto A, Tokuda M, et al. Flexible and conservative features of social systems in

tufted capuchin monkeys: comparing the socioecology of Sapajus libidinosus and Sapajus nigritus. Am J Primatol. 2012;74: 315–331. doi:10.1002/ajp.20968

35. Rímoli J, Strier K, Ferrari S. Seasonal and longitudinal variation in the behavior of free-ranging black tufted capuchins Cebus nigritus (Goldfuss, 1809) in a fragment of Atlantic Forest in Southeastern Brazil. 2008;9: 130–146.

36. Rose LM. Meat and the Early Human Diet: Insights from Neotropical Primate Studies. In: Stanford CB, Bunn HT, editors. Meat-Eating & Human Evolution. Oxford University PressNew York, NY; 2001. pp. 141–159. doi:10.1093/oso/9780195131390.003.0008

37. Canale GR, Kierulff MCM, Chivers DJ. A Critically Endangered Capuchin Monkey (Sapajus xanthosternos) Living in a Highly Fragmented Hotspot. In: Marsh LK, Chapman CA, editors. Primates in Fragments: Complexity and Resilience. New York, NY: Springer; 2013. pp. 299–311. doi:10.1007/978-1-4614-8839-2_20

38. Falótico T. Vertebrate Predation and Tool-Aided Capture of Prey by Savannah Wild Capuchin Monkeys (Sapajus libidinosus). Int J Primatol. 2022;44: 9–20. doi:10.1007/s10764-022-00320-z

39. Falótico T, Verderane MP, Mendonça-Furtado O, Spagnoletti N, Ottoni EB, Visalberghi E, et al. Food or threat? Wild capuchin monkeys (Sapajus libidinosus) as both predators and prey of snakes. Primates J Primatol. 2018;59: 99–106. doi:10.1007/s10329-017-0631-x

40. Ferreira R, Resende BD, Mannu M, Ottoni EB, Izar P. Bird predation and prey-transfer in brown capuchin monkeys (*Cebus apella*). Neotropical Primates. 2002;10: 84–89. doi:10.62015/np.2002.v10.510

41. Filho RF, Veiga S, Bezerra B. Bearded capuchin (Sapajus libidinosus) predation on a rock cavy (Kerodon rupestris) followed by prey sharing. Primates. 2021;62: 463–466. doi:10.1007/s10329-021-00894-x

42. Galetti M. Predation on the squirrel, Sciurus aestuans by capuchin monkeys, Cebus apella. Predation Squirrel Sciurus Aestuans Capuchin Monkeys Cebus Apella. 1990;54: 152–154.

43. Gentil E, Bezerra B, Medeiros LA de, Barnett AA. Predation by white-fronted capuchin monkeys, Cebus albifrons on eggs of three species of freshwater turtles in Brazilian Amazonia: solitary nests are also depredated. J Nat Hist. 2021;55: 1983–1997. doi:10.1080/00222933.2021.1977863

44. Milano MZ, Monteiro-Filho ELA. Predation on small mammals by capuchin monkeys, Cebus cay. Neotropical Primates. 2009;16: 78–80. doi:10.1896/044.016.0210

45. Newcomer MW, De Farcy DD. White-Faced Capuchin (Cebus capucinus) Predation on a Nestling Coati (Nasua narica). J Mammal. 1985;66: 185–186. doi:10.2307/1380983

46. Perry S, Rose L. Begging and transfer of coati meat by white-faced capuchin monkeys,Cebus capucinus. Primates. 1994;35: 409–415. doi:10.1007/BF02381950

47. Resende BD, Greco VLG, Ottoni EB, Izar P. Some observations on the predation of small mammals by tufted capuchin monkeys (*Cebus apella*). Neotropical Primates. 2003;11: 103–104. doi:10.62015/np.2003.v11.543

48. Rose LM, Perry S, Panger MA, Jack K, Manson JH, Gros-Louis J, et al. Interspecific Interactions Between Cebus capucinus and Other Species: Data from Three Costa Rican Sites. Int J Primatol. 2003;24: 759–796. doi:10.1023/A:1024624721363

49. Terborgh J. Five New World Primates: A Study in Comparative Ecology. Princeton University Press; 1983. doi:10.2307/j.ctt7ztr6n

50. Rodrigues KC, Delfim FR, Castro CSS de, França FGR, Filho EL, Mesquita DO, et al. Strobilurus torquatus Wiegmann, 1834 (Squamata: Tropiduridae): new records from the Brazilian state of Paraíba and a geographic distribution map. Check List. 2013;9: 614–617. doi:10.15560/9.3.614

51. Sampaio DT, Ferrari SF. Predation of an Infant Titi Monkey (Callicebus moloch) by a Tufted Capuchin (Cebus apella). Folia Primatol (Basel). 2005;76: 113–115. doi:10.1159/000083617

52. Carretero-Pinzón X, Defler TR, Ferrari SF. Observation of Black-Capped Capuchins (Cebus apella) Feeding on an Owl Monkey (Aotus brumbacki) in the Colombian Llanos. Neotropical Primates. 2008;15: 62–63. doi:10.1896/044.015.0210

53. Albuquerque NM, Silvestre SM, Cardoso TS, Ruiz-Esparza JM, Rocha PA, Beltrão-Mendes R, et al. Capture of a common marmoset (Callithrix jacchus) by

a capuchin monkey (Sapajus sp.) in the Ibura National Forest, Sergipe (Brazil). Neotropical Primates. 2014;21: 219–221. doi:10.1896/044.021.0216

54. Colan-Rodriguez RW, Herrera-Huayhua CM, Mendoza-Soto JA, Quispe-Saenz C, Reyes-Lizarraga GM, Echevarria-Macassi LA, et al. Predation on two brown-eared woolly opossums (Caluromys lanatus) by a group of large-headed capuchin monkeys (Sapajus macrocephalus) and notes on their hierarchical feeding behaviour. Neotropical Primates. 2023;29. doi:10.62015/np.2023.v29.819

55. Ezcurra MD, Scheyer TM, Butler RJ. The Origin and Early Evolution of Sauria: Reassessing the Permian Saurian Fossil Record and the Timing of the Crocodile-Lizard Divergence. PLOS ONE. 2014;9: e89165. doi:10.1371/journal.pone.0089165

56. Wright KA, Biondi L, Visalberghi E, Ma Z, Izar P, Fragaszy D. Positional behavior and substrate use in wild adult bearded capuchin monkeys (Sapajus libidinosus). Am J Primatol. 2019;81: e23067. doi:10.1002/ajp.23067

57. Truppa V, Carducci P, Sabbatini G. Object grasping and manipulation in capuchin monkeys (genera Cebus and Sapajus). Biol J Linn Soc. 2019;127: 563–582. doi:10.1093/biolinnean/bly131

58. Rose LM. Sex differences in diet and foraging behavior in white-faced capuchins (Cebus capucinus). Int J Primatol. 1994;15: 95–114. doi:10.1007/BF02735236

59. Watts DP, Mitani JC. Hunting Behavior of Chimpanzees at Ngogo, Kibale National Park, Uganda. Int J Primatol. 2002;23: 1–28. doi:10.1023/A:1013270606320

60. Gilby IC, Emery Thompson M, Ruane JD, Wrangham R. No evidence of short-term exchange of meat for sex among chimpanzees. J Hum Evol. 2010;59: 44–53. doi:10.1016/j.jhevol.2010.02.006

61. Gilby IC, Machanda ZP, O'Malley RC, Murray CM, Lonsdorf EV, Walker K, et al. Predation by female chimpanzees: Toward an understanding of sex differences in meat acquisition in the last common ancestor of *Pan* and *Homo*. J Hum Evol. 2017;110: 82–94. doi:10.1016/j.jhevol.2017.06.015

62. Key C, Ross C. Sex differences in energy expenditure in non–human primates. Proc R Soc Lond B Biol Sci. 1999;266: 2479–2485. doi:10.1098/rspb.1999.0949

63. Fragaszy DM, Izar P, Liu Q, Eshchar Y, Young LA, Visalberghi E. Body mass in wild bearded capuchins, (Sapajus libidinosus): Ontogeny and sexual dimorphism. Am J Primatol. 2016;78: 473–484. doi:10.1002/ajp.22509

64. McCabe GM, Fedigan LM. Effects of Reproductive Status on Energy Intake, Ingestion Rates, and Dietary Composition of Female Cebus capucinus at Santa Rosa, Costa Rica. Int J Primatol. 2007;28: 837–851. doi:10.1007/s10764-007-9159-z

65. Westergaard GC, Suomi SJ. Transfer of tools and food between groups of tufted capuchins (Cebus apella). Am J Primatol. 1997;43: 33–41. doi:10.1002/(SICI)1098-2345(1997)43:1%3C33::AID-AJP2%3E3.0.CO;2-Z

66. Boesch C, Boesch H. Hunting behavior of wild chimpanzees in the Taï National Park. Am J Phys Anthropol. 1989;78: 547–573. doi:10.1002/ajpa.1330780410

67. Boesch C, Kohou G, Néné H, Vigilant L. Male competition and paternity in wild chimpanzees of the Taï forest. Am J Phys Anthropol. 2006;130: 103–115. doi:10.1002/ajpa.20341

68. Falótico T, Ottoni EB. Sexual bias in probe tool manufacture and use by wild bearded capuchin monkeys. Behav Processes. 2014;108: 117–122. doi:10.1016/j.beproc.2014.09.036

69. Edwards W, Lonsdorf EV, Pontzer H. Total energy expenditure in captive capuchins (Sapajus apella). Am J Primatol. 2017;79: e22638. doi:10.1002/ajp.22638

70. Spagnoletti N, Visalberghi E, Verderane MP, Ottoni E, Izar P, Fragaszy D. Stone tool use in wild bearded capuchin monkeys, *Cebus libidinosus*. Is it a strategy to overcome food scarcity? Anim Behav. 2012;83: 1285–1294. doi:10.1016/j.anbehav.2012.03.002

71. Spagnoletti N, Visalberghi E, Ottoni E, Izar P, Fragaszy D. Stone tool use by adult wild bearded capuchin monkeys (*Cebus libidinosus*). Frequency, efficiency and tool selectivity. J Hum Evol. 2011;61: 97–107. doi:10.1016/j.jhevol.2011.02.010

72. Visalberghi E, Fragaszy D. The Etho- *Cebus* Project: Stone-tool use by wild capuchin monkeys. 1st ed. In: Sanz CM, Call J, Boesch C, editors. Tool Use in Animals. 1st ed. Cambridge University Press; 2013. pp. 203–222. doi:10.1017/CBO9780511894800.013

73. IUCN. Sapajus libidinosus: Martins, A.B., Fialho, M.S., Jerusalinsky, L., Valença-Montenegro, M.M., Bezerra, B.M., Laroque, P.O., de Melo, F.R. & Lynch Alfaro, J.W.: The IUCN Red List of Threatened Species 2021:

e.T136346A192593226. 2015. doi:10.2305/IUCN.UK.2021-1.RLTS.T136346A192593226.en

74. Martin PR, Bateson P. Measuring behaviour: an introductory guide. 3rd ed. Cambridge: Cambridge University Press; 2007.

75. Altmann J. Observational Study of Behavior: Sampling Methods. 1974 [cited 16 Sept 2025]. doi:10.1163/156853974X00534

76. Akaike H. Information Theory and an Extension of the Maximum Likelihood Principle. In: Parzen E, Tanabe K, Kitagawa G, editors. Selected Papers of Hirotugu Akaike. New York, NY: Springer New York; 1998. pp. 199–213. doi:10.1007/978-1-4612-1694-0_15

77. Bolker BM, Brooks ME, Clark CJ, Geange SW, Poulsen JR, Stevens MHH, et al. Generalized linear mixed models: a practical guide for ecology and evolution. Trends Ecol Evol. 2009;24: 127–135. doi:10.1016/j.tree.2008.10.008

78. Buckley YM. Generalized linear models. 1st ed. In: Fox GA, Negrete-Yankelevich S, Sosa VJ, editors. Ecological Statistics. 1st ed. Oxford University PressOxford; 2015. pp. 131–148. doi:10.1093/acprof:oso/9780199672547.003.0007

79. Blasco-Moreno A, Pérez-Casany M, Puig P, Morante M, Castells E. What does a zero mean? Understanding false, random and structural zeros in ecology. Methods Ecol Evol. 2019;10: 949–959. doi:10.1111/2041-210X.13185

80. Martin TG, Wintle BA, Rhodes JR, Kuhnert PM, Field SA, Low-Choy SJ, et al. Zero tolerance ecology: improving ecological inference by modelling the source

of zero observations. Ecol Lett. 2005;8: 1235–1246. doi:10.1111/j.1461-0248.2005.00826.x

81. Zuur AF, Ieno EN. A protocol for conducting and presenting results of regression-type analyses. Methods Ecol Evol. 2016;7: 636–645. doi:10.1111/2041-210X.12577

82. Harrison XA, Donaldson L, Correa-Cano ME, Evans J, Fisher DN, Goodwin CED, et al. A brief introduction to mixed effects modelling and multi-model inference in ecology. PeerJ. 2018;6: e4794. doi:10.7717/peerj.4794

83. Fox GA, Negrete-Yankelevich S, Sosa VJ, editors. Ecological Statistics: Contemporary theory and application. 1st ed. Oxford University PressOxford; 2015. doi:10.1093/acprof:oso/9780199672547.001.0001

84. Lindén A, Mäntyniemi S. Using the negative binomial distribution to model overdispersion in ecological count data. Ecology. 2011;92: 1414–1421. doi:10.1890/10-1831.1

85. Brooks ME, Kristensen K, Benthem KJ van, Magnusson A, Berg CW, Nielsen A, et al. Modeling zero-inflated count data with glmmTMB. bioRxiv; 2017. p. 132753. doi:10.1101/132753

86. Brooks M, Kristensen K, Benthem K van, Magnusson A, Berg C, Nielsen A, et al. glmmTMB Balances Speed and Flexibility Among Packages for Zero-inflated Generalized Linear Mixed Modeling. R J. 2017. Available: https://digitalcommons.unl.edu/r-journal/675

87. R Core Team. R: A language and environment for statistical computing [computer program]. Vienna: R Foundation for Statistical Computing; 2022.

88. Richards SA. Dealing with overdispersed count data in applied ecology. J Appl Ecol. 2008;45: 218–227. doi:10.1111/j.1365-2664.2007.01377.x

89. McGrew WC. Chimpanzee Material Culture: Implications for Human Evolution. Cambridge University Press; 1992.

90. Saj T, Sicotte P, Paterson JD. Influence of Human Food Consumption on the Time Budget of Vervets. Int J Primatol. 1999;20: 977–994. doi:10.1023/A:1020886820759

91. Sengupta A, McConkey KR, Radhakrishna S. Primates, Provisioning and Plants: Impacts of Human Cultural Behaviours on Primate Ecological Functions. PLOS ONE. 2015;10: e0140961. doi:10.1371/journal.pone.0140961

92. Asquith PJ. Provisioning and the study of free-ranging primates: History, effects, and prospects. Am J Phys Anthropol. 1989;32: 129–158. doi:10.1002/ajpa.1330320507

93. Bobe R, Behrensmeyer AK, Chapman RE. Faunal change, environmental variability and late Pliocene hominin evolution. J Hum Evol. 2002;42: 475–497. doi:10.1006/jhev.2001.0535

94. Hopper LM, Ross SR, Goodall J. Chimpanzees in Context: A Comparative Perspective on Chimpanzee Behavior, Cognition, Conservation, and Welfare. University of Chicago Press; 2020. doi:10.7208/chicago/9780226728032.001.0001

95. Gilby IC, Wrangham RW. Risk-prone hunting by chimpanzees (Pan troglodytes schweinfurthii) increases during periods of high diet quality. Behav Ecol Sociobiol. 2007;61: 1771–1779. doi:10.1007/s00265-007-0410-6

96. Izar P, Peternelli-dos-Santos L, Rothman JM, Raubenheimer D, Presotto A, Gort G, et al. Stone tools improve diet quality in wild monkeys. Curr Biol. 2022;32: 4088-4092.e3. doi:10.1016/j.cub.2022.07.056

97. Goffe AS, Fischer J. Meat sharing between male and female Guinea baboons (*Papio papio*). Primate Biol. 2016;3: 1–8. doi:10.5194/pb-3-1-2016

98. Hamilton WJ III, Busse CD. Primate Carnivory and Its Significance to Human Diets. BioScience. 1978;28: 761–766. doi:10.2307/1307249

99. Harding R. Meat-eating and hunting in baboons. Socioecology and psychology of primates. The Hague: Mouton; 1975. pp. 245–257.

100. Hausfater G. Predatory Behavior of Yellow Baboons. Behaviour. 1976;56: 44–68. Available: https://www.jstor.org/stable/4533713

101. Rhine RJ, Norton GW, Wynn GM, Wynn RD, Rhine HB. Insect and meat eating among infant and adult baboons (Papio cynocephalus) of Mikumi National Park, Tanzania. Am J Phys Anthropol. 1986;70: 105–118. doi:10.1002/ajpa.1330700115

102. Strum SC. 8. Processes and Products of Change: Baboon Predatory Behavior at Gilgil, Kenya. In: Harding RSO, Teleki G, editors. Omnivorous Primates Gathering and Hunting in Human Evolution. Columbia University Press; 2019. pp.

255–302. Available: https://www.degruyterbrill.com/document/doi/10.7312/hard92188-009/html

103. Anderson A, Chilczuk S, Nelson K, Ruther R, Wall-Scheffler C. The Myth of Man the Hunter: Women's contribution to the hunt across ethnographic contexts. PLOS ONE. 2023;18: e0287101. doi:10.1371/journal.pone.0287101

104. Venkataraman VV, Hoffman J, Farquharson K, Davis HE, Hagen EH, Hames RB, et al. Female foragers sometimes hunt, yet gendered divisions of labor are real: a comment on Anderson et al. (2023) The Myth of Man the Hunter. Evol Hum Behav. 2024;45: 106586. doi:10.1016/j.evolhumbehav.2024.04.014

105. Marlowe FW. Hunter-gatherers and human evolution. Evol Anthropol Issues News Rev. 2005;14: 54–67. doi:10.1002/evan.20046

106. Marlowe FW. Hunting and Gathering: The Human Sexual Division of Foraging Labor. Cross-Cult Res. 2007;41: 170–195. doi:10.1177/1069397106297529

107. Murdock GP, Provost C. Factors in the Division of Labor by Sex: A Cross-Cultural Analysis. Ethnology. 1973;12: 203–225. doi:10.2307/3773347

108. Goodman MJ, Griffin PB, Estioko-Griffin AA, Grove JS. The compatibility of hunting and mothering among the agta hunter-gatherers of the Philippines. Sex Roles. 1985;12: 1199–1209. doi:10.1007/BF00287829

109. Hewlett BS. Intimate fathers : the nature and context of Aka pygmy paternal infant care. Ann Arbor : University of Michigan Press; 1991. Available: http://archive.org/details/intimatefathersn0000hewl

110. Noss AJ, Hewlett BS. The Contexts of Female Hunting in Central Africa. Am Anthropol. 2001;103: 1024–1040. doi:10.1525/aa.2001.103.4.1024

111. Tonkinson R. The Mardudjara Aborigines: Living the Dream in Australia's Desert. Holt, Rinehart and Winston; 1978.

112. Gilby IC, Machanda ZP, O'Malley RC, Murray CM, Lonsdorf EV, Walker K, et al. Predation by female chimpanzees: Toward an understanding of sex differences in meat acquisition in the last common ancestor of *Pan* and *Homo*. J Hum Evol. 2017;110: 82–94. doi:10.1016/j.jhevol.2017.06.015

113. McGrew WC, Tutin CEG, Baldwin PJ. Chimpanzees, Tools, and Termites: Cross-Cultural Comparisons of Senegal, Tanzania, and Rio Muni. Man. 1979;14: 185–214. doi:10.2307/2801563

114. Tennie C, O'Malley RC, Gilby IC. Why do chimpanzees hunt? Considering the benefits and costs of acquiring and consuming vertebrate versus invertebrate prey. J Hum Evol. 2014;71: 38–45. doi:10.1016/j.jhevol.2014.02.015

115. Bird R. Cooperation and conflict: The behavioral ecology of the sexual division of labor. Evol Anthropol Issues News Rev. 1999;8: 65–75. doi:10.1002/(SICI)1520-6505(1999)8:2%3C65::AID-EVAN5%3E3.0.CO;2-3

116. Aquino ID, González-Santoyo I, Link A, Muñoz-Delgado J. An exploratory study of cooperation: food-sharing behaviour in wild varied white-fronted capuchin monkeys (Cebus versicolor) in Central Colombia. 2022 [cited 14 Sept 2025]. doi:10.1163/1568539X-bja10180

117. Samuni L, Preis A, Deschner T, Crockford C, Wittig RM. Reward of labor coordination and hunting success in wild chimpanzees. Commun Biol. 2018;1: 138. doi:10.1038/s42003-018-0142-3

118. Kyriacou K, Blackhurst DM, Parkington JE, Marais AD. Marine and terrestrial foods as a source of brain-selective nutrients for early modern humans in the southwestern Cape, South Africa. J Hum Evol. 2016;97: 86–96. doi:10.1016/j.jhevol.2016.04.009

119. Woodard HQ, White DR. The composition of body tissues. Br J Radiol. 1986;59: 1209–1218. doi:10.1259/0007-1285-59-708-1209

120. Gilby IC, Wawrzyniak D. Meat Eating by Wild Chimpanzees (Pan troglodytes schweinfurthii): Effects of Prey Age on Carcass Consumption Sequence. Int J Primatol. 2018;39: 127–140. doi:10.1007/s10764-018-0019-9

121. Goodall J. The chimpanzees of Gombe: patterns of behavior. Cambridge, Massachusetts London, England: The Belknap Press of Harvard University Press; 1986.

122. Pruetz JD, Bertolani P, Ontl KB, Lindshield S, Shelley M, Wessling EG. New evidence on the tool-assisted hunting exhibited by chimpanzees (Pan troglodytes verus) in a savannah habitat at Fongoli, Sénégal. R Soc Open Sci. 2015;2: 140507. doi:10.1098/rsos.140507

Supporting information

S1 File. Cleaning and pre-processing.

S2 File. Study Data.

S1 Fig. Full dataset, showing all predation events per individual and month. The size of the circles indicates the number of events (from 0 to 6) in each month and the circle color indicates the age class to which the individual belongs each month.

S2 Fig. Predations events across the study period 56 months, starting in May 2006. **N**otice the 11 months gap from May 2008 to March 2009. Group information is also provided.

S3 Fig. Full dataset after cleaning. Includes 10 fewer individuals, showing all predation events per individual and month. The sex of each individual is indicated by color (red = male, blue = female).

S1 Table. Variance Inflation Factors (VIF) for the Consumption Model. VIF values < 5 indicate low multicollinearity; values near 1 indicate nearly no correlation between predictors.

S2 Table. Sub-model of mammalian consumption. Number of observations: 1092, random effects: ID (36); Month (45). Final model metrics: AIC = 502.6; BIC = 572.5; logLik = -237.3; deviance = 474.6; df residuals = 1078.

S3 Table. Sub-model of saurian consumption. Number of observations: 1092, random effects: ID (36); Month (45). Final model metrics: AIC = 920.3; BIC = 990.2; logLik = -446.1; deviance = 892.3; df residuals = 1078.

S1 Video. Vertebrate consumption. (a) A young capuchin eating a vertebrate; (b) Capuchin feeding on a lizard. This video was filmed by Alessandro Albani for the documentary "The bearded capuchin monkeys of Fazenda Boa Vista" (https://www.youtube.com/watch?v=7ojU43JgpYo), not recorded during the study, but it illustrates what we report here.

S2 Video. A hunting event of Type II (collective). Four young capuchins hidden in a bush attack an Iguana (*Iguana iguana*) passing nearby. One grasps and detaches the far end of the iguana tail. Meat transfer should have occurred while the capuchins were into the bush. However, it is important to note that sooner or later we observed all four of them eating parts of the iguana. This video was filmed by Alessandro Albani for the documentary "The bearded capuchin monkeys of Fazenda Boa Vista" (https://www.youtube.com/watch?v=7ojU43JgpYo), not recorded during the study, but it illustrates what we report here.

S3 Video. Food transfer. (a) A Subadult male has captured a great kiskadee (*Pitangus sulphuratus*) and moves in the canopy holding its carcass. (b) Other group members show interest towards it and approach the possessor. (c) Food transfer may occur with the possessor tolerating the recipient to take a piece of the prey. (d) An infant, who has obtained mostly bones with very little meat, is chewing the same kiskadee initially captured by the subadult male. This video was filmed by Alessandro Albani for the documentary "The bearded capuchin monkeys of Fazenda Boa Vista" (https://www.youtube.com/watch?v=7ojU43JgpYo), not recorded during the study, but it illustrates what we report here.

S1 Checklist. Inclusivity in global research information. Additional information regarding the ethical, cultural, and scientific considerations specific to inclusivity in global research in this study.

Figure 1

Click here to access/download;Figure;Fig_1.tif

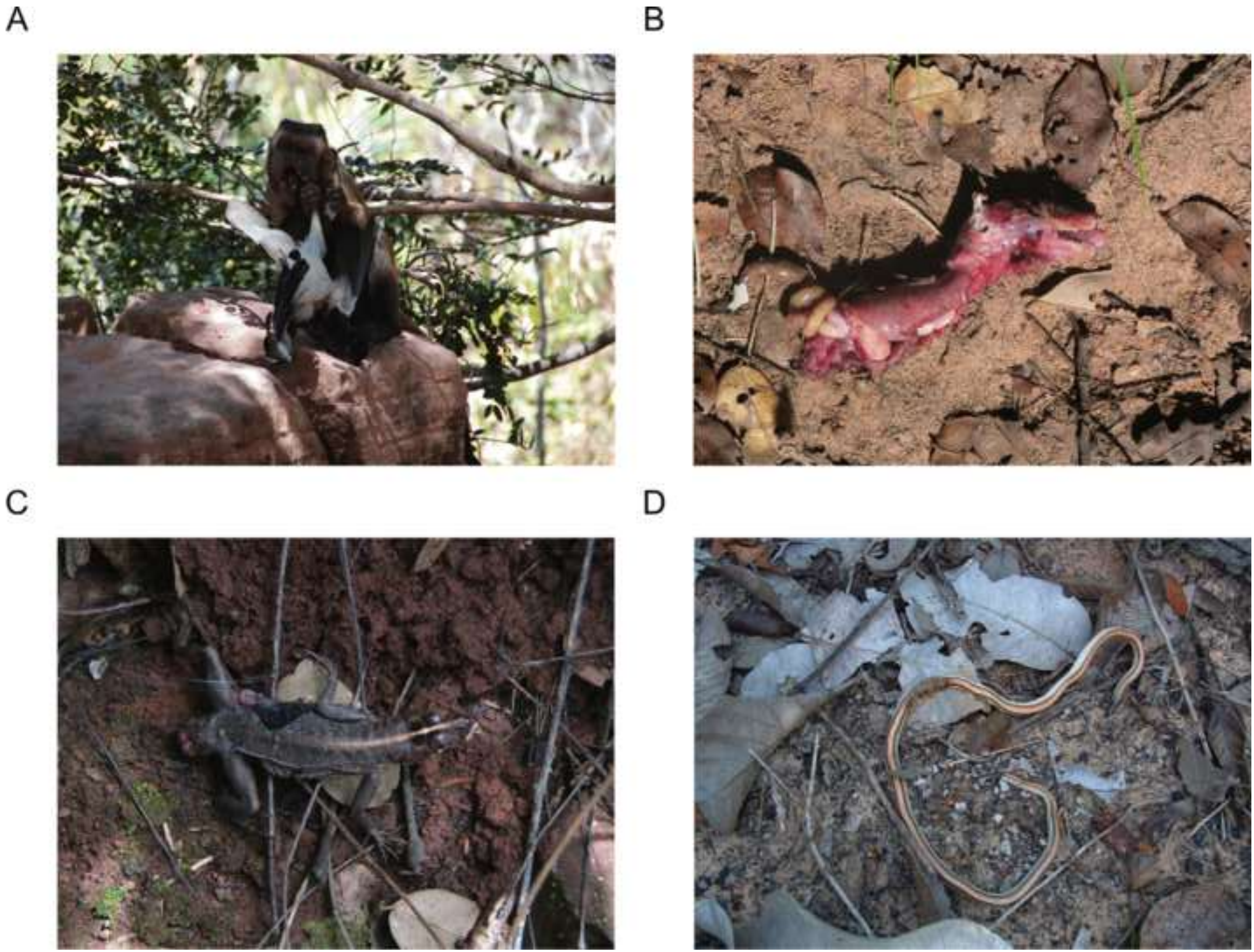

Figure 2

Click here to access/download;Figure;Fig_2.tif

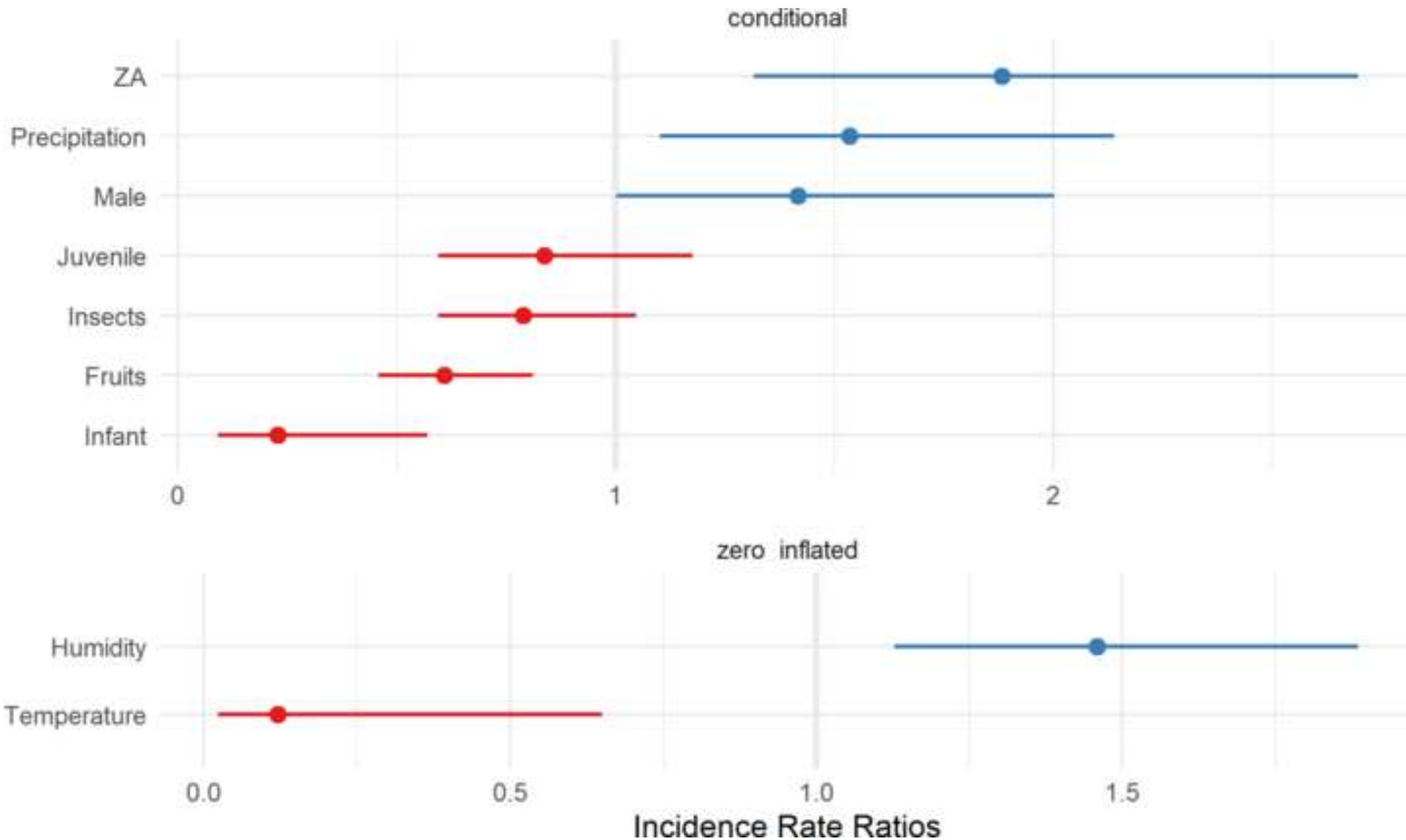

Figure 3

Click here to access/download;Figure;Fig_3.tif

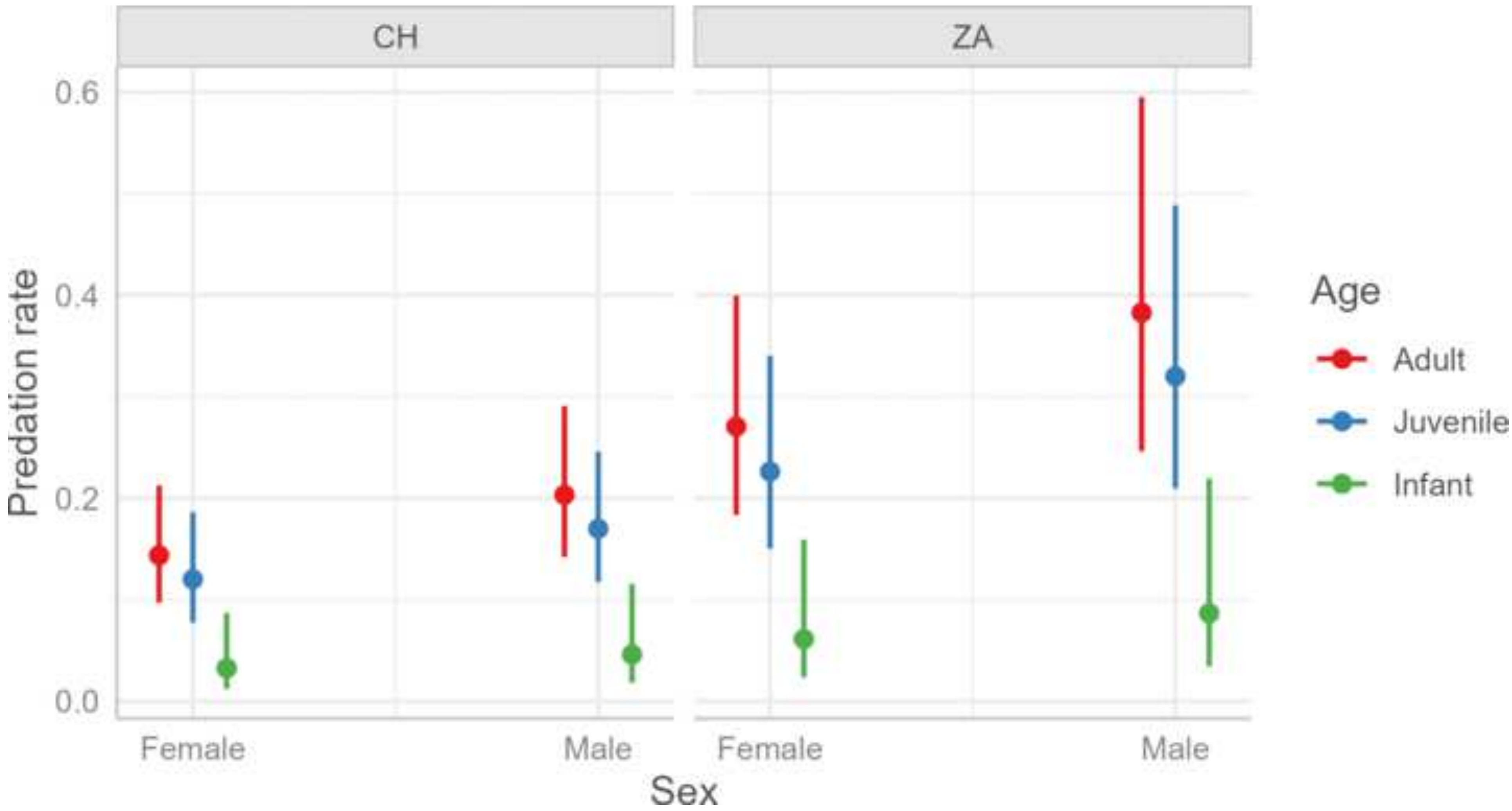

Figure 4

Click here to access/download;Figure;Fig_4.tif

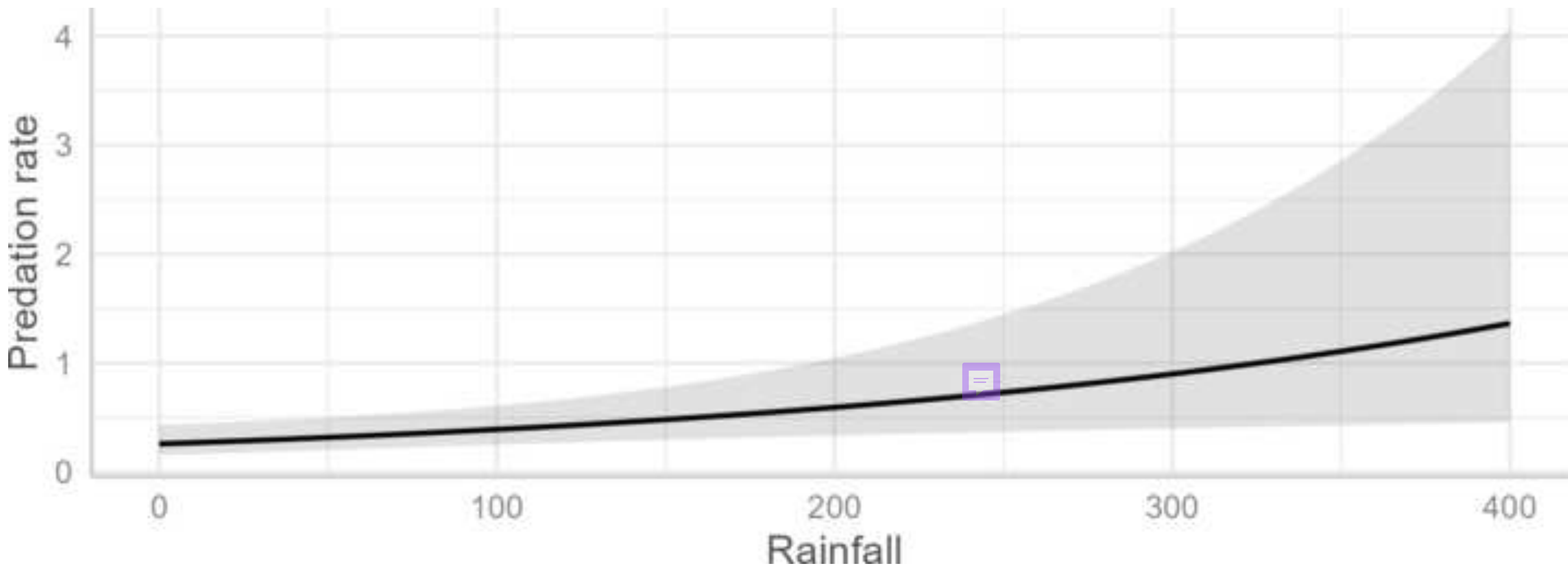

Click here to access/download;Figure;Fig_5.tif

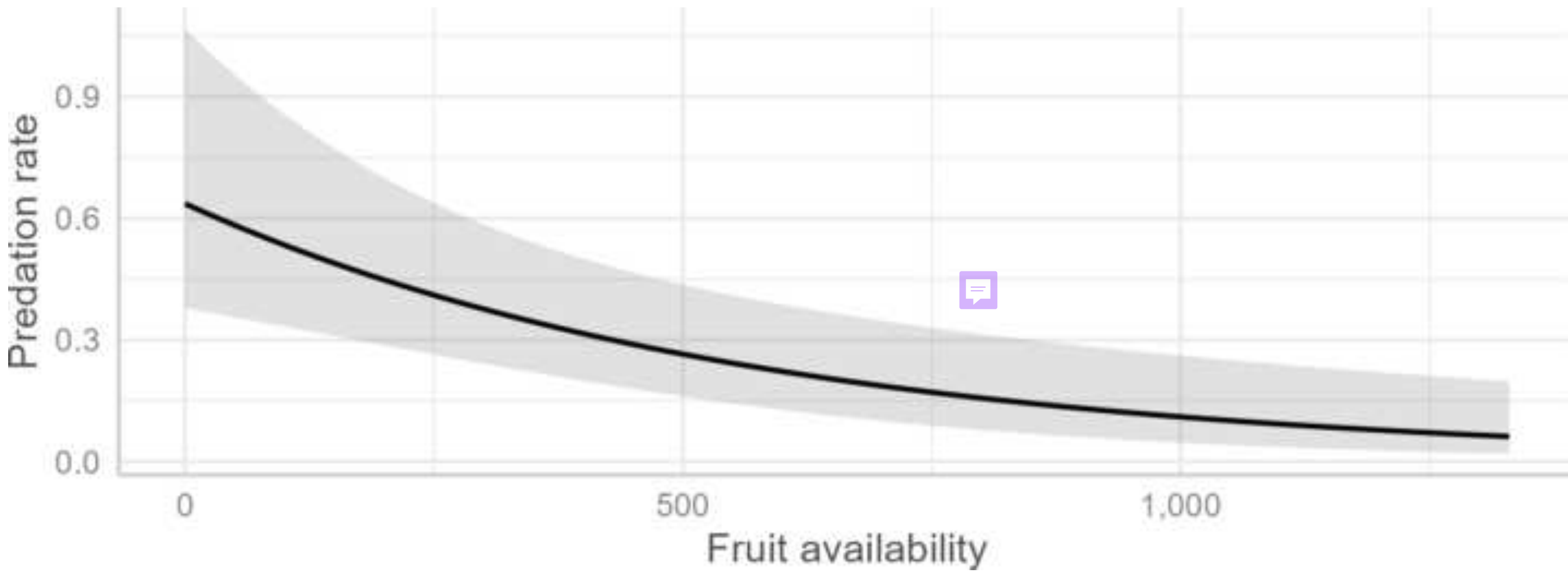

Figure 6

Click here to access/download;Figure;Fig_6.tif

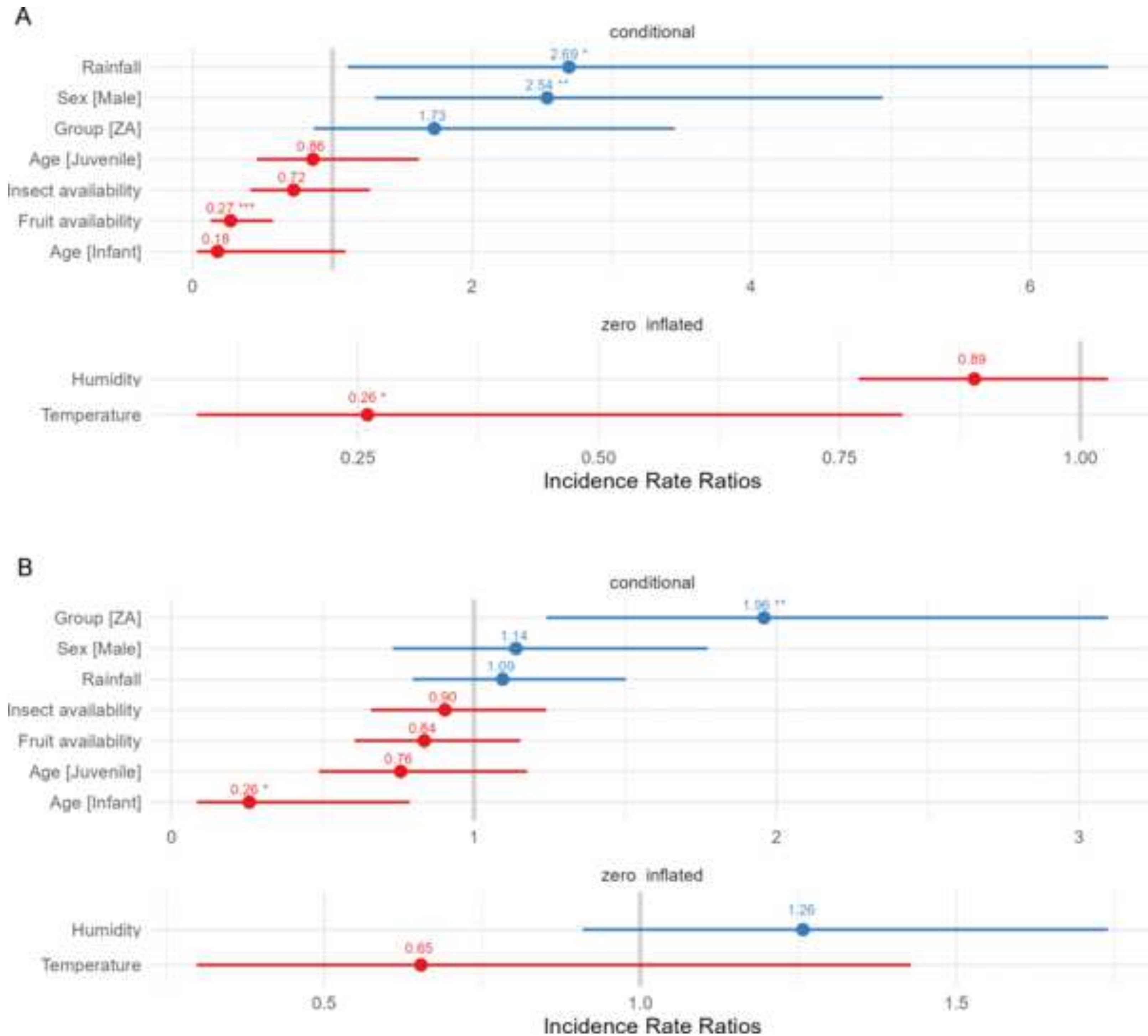

S1 Fig

Click here to access/download
Supporting Information
S1_Fig.tif

S2 Fig

Click here to access/download
Supporting Information
S2_Fig.tif

S3 Fig

Click here to access/download
Supporting Information
S3_Fig.tif

S1 Table

Click here to access/download
Supporting Information
S1_Table.docx

S2 Table

Click here to access/download
Supporting Information
S2_Table.docx

S3 Table

Click here to access/download
Supporting Information
S3_Table.docx

S1 File

Click here to access/download
Supporting Information
S1_File.docx

S1 Checklist: questionnaire on inclusivity in global research

Click here to access/download
Supporting Information
S1_Checklist.docx

S1 Video

Click here to access/download

Supporting Information - Compressed/ZIP File Archive

S1_Video.mp4.zip

S2 Video

Click here to access/download
Supporting Information - Compressed/ZIP File Archive
S2_Video.mp4.zip

S3 Video

Click here to access/download

Supporting Information - Compressed/ZIP File Archive

S3_Video.mp4.zip

Small vertebrate consumption by capuchin monkeys (*Sapajus libidinosus*): Insights into the human predatory pattern

Susana Carvalho[1,2¶*], Noemi Spagnoletti[3,4,5¶], João d'Oliveira Coelho[1,2,6], Megan Beardmore-Herd[2,6], Olívia Mendonça-Furtado[3], Michele Verderane[3], Joana Prieto[7], Dominique I. Cavallaro[8], Patrícia Izar[3], Elisabetta Visalberghi[5]

[1] Department of Science, Gorongosa National Park, Sofala, Mozambique

[2] CIBIO/InBIO, Centro de Investigação em Biodiversidade e Recursos Genéticos, Universidade do Porto, Campus de Vairão, Vairão, Portugal

[3] Institute of Psychology, Department of Experimental Psychology, University of São Paulo, Av. Prof. Mello Moraes 1721, CEP 05508-030, Cidade Universitária, São Paulo, Brazil

[4] Global Mammal Assessment Lab, Department of Biology and Biotechnology Charles Darwin, Sapienza University of Rome, Rome, Italy

[5] Istituto di Scienze e Tecnologie della Cognizione, Consiglio Nazionale delle Ricerche, ~~00197~~ Rome, Italy

[6] Primate Models for Behavioural Evolution Lab, Institute of Human Sciences, University of Oxford, UK

[7] Department of Life Sciences, University of Coimbra, ~~Apartado 3046, 3001-401~~ Coimbra, Portugal

[8] School of Anthropology, Pontificia Universidad Católica de Chile, Santiago de Chile, Chile

*Corresponding author.

E-mail: susana.carvalho@gorongosa.net

¶These authors contributed equally to this work

Formatted: Font: 12 pt

ORCID

Noemi Spagnoletti: 0000-0002-8285-5963

Olivia Mendonça Furtado: 0000-0002-0643-5414

Joana Prietro: 0009-0000-8089-3416

Michele Verderane: 0000-0001-8467-8385

Elisabetta Visalberghi: 0000-0001-7407-5468

Susana Carvalho: 0000-0003-4542-3720

Dominique Cavallaro: 0009-0009-8667-7330

Megan Beardmore-Herd: 0009-0005-7681-6510

João M. P. Coelho: 0000-0002-4339-0550

Patrícia Izar: 0000-0001-6741-0731

Abstract

The consumption of small vertebrates occurs to varying degrees among many non-human primates and may have constituted an important source of nutrition for early hominins, particularly during periods of food scarcity, providing a stepping stone to the exploitation of larger animals. Capuchin monkeys regularly consume small vertebrates across their range. Expanding our knowledge of the contribution of animal matter to these primates' diets can shed light on the proximate and ultimate drivers of small vertebrate consumption across primate taxa and help evaluate hypotheses on the role of small prey exploitation in human evolution. We report on the most extensive record of bearded capuchin (*Sapajus libidinosus*) vertebrate predation so far available: 45 months of observations of vertebrate predation in two sympatric groups in Fazenda Boa Vista, Brazil, one of which was

provisioned daily. We estimated the effect of provisioning, sex, age class, fruit and insect availability, rainfall, humidity, and temperature on predation rates using a zero-inflated negative binomial algorithm with random effects. We found mammal and saurian consumption is habitual in this population of *S. libidinosus*, and prey brains and viscera tend to be accessed before the rest of the body. Predation rates were higher in males compared to females and in adults compared to infants across groups. Our results also identified small vertebrates as key energy sources for bearded capuchins under decreased fruit availability and show that provisioning reduces predation rates. Future work should assess small vertebrate predation as a possible stepping stone in the emergence of human exploitation of large animals by re-analyzing fossil faunal assemblages for evidence of small vertebrate predation and identifying the traces left by the exploitation of small prey in capuchins and other extant primates through neo-taphonomy.

Key words: capuchin behaviour, predatory patterns, foraging ecology, vertebrate consumption, availability of resources

Introduction

The habitual acquisition and consumption of animal matter is a shared behaviour among humans and non-human primates (hereafter primates) [1]. Despite this, humans are the only extant primate known to regularly exploit animals as large as, or larger than, themselves for energetic gains [2]. This strategy has been termed the "human predatory pattern" and is hypothesised to have originated from percussion-based scavenging of large animal carcasses in order to access inside-bone nutrients [3]. The increased energy content associated with this dietary shift may have enabled the

evolution of larger brains, and the adaptive suite of defining human characteristics that came with it [4–12]. Furthermore, in combination with cooperative hunting and technological advances, accessing animal resources would have acted as a buffer to seasonality in plant food availability [3,13].

Prior to the emergence of the human predatory pattern, small vertebrates (< 5 kg) may have constituted an important source of nutrition for hominins [14]. Being easier and less costly to catch than larger prey, as well as more widely distributed, the opportunistic exploitation of small animals could have been a crucial stepping stone, later evolving into the regular, tool-assisted exploitation of larger prey through passive or active scavenging and hunting. Support for this can be found in the zooarchaeological record, with faunal assemblages dating to around 2 million years ago providing direct evidence of sustained terrestrial and aquatic small animal resource exploitation by hominins with limited technological repertoires [15,16]. Furthermore, small game is a resource regularly accessed by several ~~contemporary~~

~~Further support for the importance of small animal consumption comes from studies of contemporary~~ modern human hunter-gatherer populations, among them~~such as~~ the !Kung of Botswana ~~where small game is a vital resource~~ [17] and the Hadza of Tanzania ~~where small vertebrates are regularly exploited by hunters, despite being targeted less often than large game~~ [18]. There is also zooarchaeological evidence of ~~from hunter-gatherers of North America reporting on~~ the consumption of small vertebrates by Holocene North-American hunter-gatherers ~~via analysis of coprolites~~ [19]~~,~~ as well as ~~and of small mammal consumption by the~~ populations from the~~of the~~ Patagonian Andes, in Argentina~~, both during the Holocene~~ [20].

In the most recent review of meat eating by ~~nonhuman~~ primates available, ingestion of vertebrate tissue was noted to occur in at least 89 species, and was ~~being~~ most common amongst highly encephalized species like capuchin monkeys (*Cebus* and *Sapajus* ~~spp.~~), baboons (*Papio* ~~spp.~~), bonobos (*Pan paniscus*), and chimpanzees (*Pan troglodytes*) [1]. Across these primates, t~~T~~he prey classes most frequently targeted ~~consumed~~ were ~~found to be~~ small vertebrates, including~~such as~~ birds, reptiles, amphibians, and mammals, although ~~rates of~~ consumption rates ~~and prey species targeted~~ varied ~~both~~ between and within species~~, as well as~~~~and within sites over time~~ [21]. Chimpanzees are reported to ~~To date, much of the primate meat eating literature has focused on chimpanzees, the primate which is reported to~~ prey upon the widest range of vertebrate species [1,2], followed by bonobos [1].~~. Across several sites, chimpanzees are known to regularly prey upon numerous species of mammals, as well as birds and reptiles, although less frequently (22), following a systematic pattern in a variety of environments (23). Colobine monkeys appear to be the most commonly captured prey (22, 24, 25), and predatory activities assume social relevance within chimpanzee groups, playing a role in relation to mating opportunities and cooperation among males (26). Bonobo vertebrate predation accounts have dramatically increased since the first extensive review of vertebrate predation by primates (2), with bonobos now falling second only to chimpanzees in terms of the range of vertebrate prey reported to be consumed (1). As with chimpanzees, the consumption of other primates has been documented only in certain bonobo populations (27), though this behaviour appears to be much more rare, partially accounting for the overall lower rates of hunting among bonobos compared to chimpanzees (1).~~ Baboons also have ~~are also noteworthy among the primates for their~~ broad prey repertoires, which, for some species, ~~also~~ include relatively large mammals such as ~~domestic~~ goats, sheep, and dogs [22–24].

Capuchin monkeys are currently classified into four species of gracile capuchins (genus *Cebus*) and eight of robust capuchins (genus *Sapajus*) [25,26]. All members~~species~~ are highly opportunistic, generalist omnivores known for their dietary flexibility, ~~including~~ their ability to prey upon fast-moving (and often stinging and biting) invertebrates and small vertebrates, and their extraction and processing of difficult-~~-~~to-access food items using advanced cognitive and complex manipulative skills [27–32]. ~~In some capuchin populations, vertebrate hunting and capture rates are within ranges similar to several chimpanzee populations (1) and vertebrate consumption may account for 2-3% of overall feeding time and up to 15% for some individuals during peak predation months (37, 52).~~ They also have high levels of behavioural flexibility, allowing them to adapt to distinct~~different~~ local conditions ~~by exploiting several different habitats and manipulating food sources~~ [33–35]. As a result of this, capuchin monkeys occupy a wide geographic distribution throughout Central and South America, from Honduras to northern Argentina [25,26], and cover a wide range of habitats, spanning from dry seasonal forests to semi-arid regions, mangroves, and tropical evergreen forests [29].

In some capuchin populations, vertebrate hunting and capture rates are within ranges similar to several chimpanzee populations [1] and vertebrate consumption may account for 2-3% of overall feeding time and up to 15% for some individuals during peak predation months [31,36]. The list of vertebrates that capuchin monkeys ~~are reported to~~ prey upon varies depending on site and species (Table 1), and continues to expand as available evidence grows ~~and is ever growing as the number of studies increases~~. To date, this list includes ~~Prey cited most regularly are~~ mammals, such as squirrels (*Sciurus* sp.), coatis (*Nasua narica*), rodents (including the large rock cavie, *Kerodon rupestris*), juvenile anteaters (*Tamandua mexicana*) and bats; reptiles and amphibians, including snakes, lizards, frogs, and freshwater turtle eggs; and birds of several~~many~~ species

[28,29,31,32,36–50]. Capuchins have also been observed to kill potentiallyavoid dangerous poisonous and constrictor snake speciess, but they prey on non-dangerous onessnakes (43, 55). Capuchin monkeys also capture and feed upon other primates. In Ilha de Germoplasma in eastern Brazil a tufted capuchin monkey (*Cebus apella*; *S. apella*) was seen to kill and feed upon an infant dusky titi monkey (*Plecturocebus moloch*) [51]. A group of the same capuchin species was observed feeding on the carcass of an adult owl monkey (*Aotus brumbacki*) in what appeared to be a scavenging event in the Colombian Llanos [52]. Additionally, an adult male capuchin monkey (*Sapajus* sp.) in the Ibura National Forest of Brazil was reported to capture and presumably ingest a common marmoset (*Callithrix jacchus*), although predation was not observed directly [53].

Formatted: Font: Italic

Formatted: Font: Italic

Formatted: Font: Italic

Formatted: Font: Italic

Formatted: Font: Italic

Formatted: Font: Italic

Table 1. Vertebrates in the diet of capuchin monkeys.

Prey class	Prey type	Capuchin species	Site	Citations
Saurian *	Lizard	Bearded capuchin (*Sapajus libidinosus*)	Serra da Capivara National Park (Brazil)	[38]
		Golden-bellied capuchin (*Sapajus xanthosternos*)	Una Biological Reserve (Brazil)	[37]

		Blond capuchin (*Sapajus flavius*)	Caatinga dry forest area (Brazil)	[32]
			Reserva Particular do Patrimônio Natural Engenho Gargaú (Brazil)	[50]
		White-faced capuchin (*Cebus capucinus*)	Santa Rosa National Park (Costa Rica)	[28,48]
			Lomas Barbudal (Costa Rica)	[48]
	Snake	Bearded capuchin (*Sapajus libidinosus*)	Serra da Capivara National Park (Brazil)	[38,39]
			Fazenda Boa	[39]

Formatted: Font: (Default) Times New Roman

Formatted: Font: (Default) Times New Roman

Formatted: Font: (Default) Times New Roman, Italic

Formatted: Font: (Default) Times New Roman

Formatted: Font: (Default) Times New Roman

Formatted: Font: (Default) Times New Roman

			Vista (Brazil)	
	Turtle egg	White-fronted capuchin (*Cebus albifrons*)	Jaú National Park (Brasil)	[43]
	Iguana	White-faced capuchin (*Cebus capucinus*)	Santa Rosa National Park (Costa Rica)	[48]
	Adult bird	Bearded capuchin (*Sapajus libidinosus*)	Serra da Capivara National Park (Brazil)	[38]
		White-faced capuchin (*Cebus capucinus*)	Santa Rosa National Park (Costa Rica)	[28,31,48]
		Sapajus spp. (Captive hybrid)	Tietê Ecological Park (Brazil)	[40]
	Bird egg/nestling	White-faced capuchin	Santa Rosa National Park	[28,31,48]

		(*Cebus* capucinus)	(Costa Rica)	
			Lomas Barbudal (Costa Rica)	[48]
			Palo Verde (Costa Rica)	[48]
		Sapajus spp. (Captive hybrid)	Tietê Ecological Park (Brazil)	[40]
Mammal	Rodent	Bearded capuchin (*Sapajus libidinosus*)	Serra da Capivara National Park (Brazil)	[38]
			Serra das Almas Private Reserve of Natural Heritage (Brazil)	[41]
		Azaras's capuchin	Cabeceira do Prata Private	[44]

		(*Sapajus cay*)	Reserve (Brazil)	
		Sapajus spp. (Captive hybrid)	Tietê Ecological Park (Brazil)	[47]
		White-faced capuchin (*Cebus capucinus*)	Santa Rosa National Park (Costa Rica)	[31,48]
			Lomas Barbudal (Costa Rica)	[48]
	Bat	Bearded capuchin (*Sapajus libidinosus*)	Serra da Capivara National Park (Brazil)	[38]
		White-faced capuchin (*Cebus capucinus*)	Santa Rosa National Park (Costa Rica)	[28,31,48]
			Lomas Barbudal	[48]

			(Costa Rica)	
	Squirrel	White-faced capuchin (*Cebus capucinus*)	Santa Rosa National Park (Costa Rica)	[28,31]
			Lomas Barbudal (Costa Rica)	[48]
			Palo Verde (Costa Rica)	[48]
		Black capuchin (*Sapajus nigritus*)	Santa Genebra (Brazil)	[42]
	Coati	White-faced capuchin (*Cebus capucinus*)	Santa Rosa National Park (Costa Rica)	[28,31,45,46]
			Lomas Barbudal (Costa Rica)	[48]
			Palo Verde (Costa Rica)	[48]

	Marsupial	*Sapajus* spp. (Captive hybrid)	Tietê Ecological Park (Brazil)	[47]
		Large-headed capuchin (*Sapajus macrocephalus*)	Manu Learning Centre Biological Station (Peru)	[54]
	Anteater (juvenile)	White-faced capuchin (*Cebus capucinus*)	Lomas Barbudal (Costa Rica)	[48]
	Primate	Brown or tufted capuchin (*Sapajus apella*)	Ilha de Germoplasma (Brazil)	[51]
			Arrayanes Farm (Colombia)	[52]
		Sapajus spp. (unidentified)	Ibura National Forest (Brazil)	[53]
Amphibian	Frog	White-faced	Santa Rosa	[48]

		capuchin (*Cebus capucinus*)	National Park (Costa Rica)	

*Clade composed of Lepidosauromorpha (snakes, lizards and rhynchocephalians) and Archosauromorpha (birds and crocodilians) [55].

~~Capuchin monkeys also capture and feed upon other primates, with anecdotal reports stemming from several sites. IOn Ilha de Germoplasma in eastern Brazil, for example, a tufted capuchin monkey (*Cebus apella*; *S. apella*) was seen to kill and feed upon an infant dusky titi monkey (*Callicebus moloch*) (57). A group of the same capuchin species waswere observed feeding on the carcass of an adult owl monkey (*Aotus brumbacki*) in what appeared to be a scavenging event in the Colombian Llanos (58). Additionally, an adult male capuchin monkey (*Sapajus* sp.) in the Ibura National Forest of Brazil was reported to capture and presumably ingest a common marmoset (*Callithrix jacchus*), although predation was not observed directly (59).~~

The regular access to vertebrate prey of smaller and even similar size to their own (e.g., primates [52,53], rock cavy [41], anteater juveniles [48]) makes the capuchin dietary pattern an interesting model of the human predatory pattern [3] and the habitual small animal consumption that may have preceded its emergence [14]. In addition to commonly consuming vertebrates in the wild [28,31,38], capuchins exhibit a high degree of terrestriality (27% of their daily time is spent on the ground [56]), have complex food acquisition practices involving tool use and high levels of manual dexterity [57], and have the largest relative brain sizes of the platyrrhine primates [29]. Continuing to expand our knowledge of the contribution of animal matter to these primates' diets and the contexts

in which vertebrate predation occurs in underrepresented wild populations allows us to shed light on the ultimate and proximate drivers of this behaviour, and reflect on the role of animal matter in human evolution.

A wealth of data on capuchin monkey diet, including the contribution of vertebrate hunting, comes from the long term study of habituated, white-faced capuchin monkeys (*C. capucinus*) inhabiting a highly seasonal, tropical dry forest environment in Santa Rosa National Park, Costa Rica (33, 34, 37, 52, 53). Over several decades, a number of studies focussed on the hunting behaviour of this population, with reports suggesting that vertebrate species may account for 2-3% of overall feeding time and up to 15% for some individuals during peak predation months (37, 52). When reviewed by (1), the hunting and capture rates of the Santa Rosa capuchin monkeys for all prey types and for mammals were found to fall within the range of chimpanzee populations from sites such as Sonso, Kahuzi-Biega, Fongoli, Mahale, and Ngogo where hunting is well documented. At the nearby Costa Rican site of Lomas Barbudal, hunting by white-faced capuchin monkeys is also relatively common (53). Although overall rates are more modest than at Santa Rosa, when it comes to squirrel prey, Lomas Barbudal capuchin monkeys were found to have fewer encounters but were around twice as likely to hunt when they did and also had higher success rates (53).

Research from Santa Rosa and Lomas Barbudal sites has also offered insights into hunting patterns, affording a closer look at who is hunting and when, and thus a window into the mechanisms that may be driving hunting behaviour. Sex differences in predation rates have been noted for white-faced capuchin monkeys at Santa Rosa, with males attempting predation on more occasions than females, catching more vertebrate prey of all types than females (34, 53, 63), and having higher rates of success (37). Similar results have been found for one population of bearded capuchins (42), for chimpanzees (26, 64,

65), and across the primate order more broadly (1, 2). However, at the site of Lomas Barbudal, one study found that sex did not have a pronounced effect on hunting by white-faced capuchin monkeys, with adult males and adult females catching equal numbers of squirrels. According to (53), hunts involving multiple prey targets were more common at Lomas Barbudal, allowing females an increased opportunity to participate. Adult males were the most common sex-age class observed preying on vertebrates also in Serra da Capivara National Park (42).

The generally observed male bias in predation rates (34, 37, 42, 53, 63) can be attributed to sexual dimorphism in body mass demanding different caloric intake demands (66). At Fazenda Boa Vista, the only field site where bearded capuchins (*S. libidinosus*) have been systematically weighed, the average body mass of adult male capuchins is 3.5 kg while that of adult female capuchin is 2.1 kg (67). Thus, adult male capuchins may engage in more vertebrate consumption events than adult females to meet their greater energetic needs (63). Furthermore, whilst reproduction and lactation increase the energetic needs of females (68), they appear to buffer this through a strategy of targeting reliable, low risk food items, such as invertebrates, which would explain why vertebrate hunting, which likely requires greater energy expenditure and results in unpredictable returns, may be less common among females (34, 65). In line with this, studies have found that females consume more invertebrates than males (63), and that invertebrate consumption is vital for allowing female capuchin monkeys to meet their overall energetic needs, particularly during lactation (33).

Sex biases in vertebrate predation rates have been reported in some capuchin populations [28,31,38,48,58]. For example, at the long-term site of Santa Rosa National Park, Costa Rica, white-faced capuchin males have been observed attempting predation

on more occasions than females, catching more vertebrate prey [28,48,58], and having higher predation success rates [31]. Similar results have been found in chimpanzees [59–61], and across the primate order more broadly [1,2]. This sexual bias has been hypothesized to be linked to sexual dimorphism in body mass demanding different caloric intake demands from males compared to females [62]. At Fazenda Boa Vista, the only field site where bearded capuchins (*S. libidinosus*) have been systematically weighed, the average body mass of adult subordinate male capuchins is 3.5 kg (range: 3.4–3.6 kg) while that of adult female capuchins is 2.1 kg (range: 1.8–2.6 kg) [63]. Thus, adult male capuchins may engage in more vertebrate consumption events than adult females to meet their greater energetic needs [58]. Furthermore, whilst reproduction and lactation increase the energetic needs of females [64], they may buffer this demand by targeting reliable, low-risk food items, such as invertebrates [27], as opposed to vertebrate prey, the hunting which likely requires greater energy expenditure and results in unpredictable returns. This is supported by reports of female capuchins consuming more invertebrates than males on average [58].

Formatted: Font: Italic

There is less evidence~~consensus~~ on the effect of seasonality on vertebrate predation rates among~~in~~ capuchin monkeys~~, with the relationship appearing to be more nuanced, possibly reflecting variability in seasonal patterns from year to year and the resulting effects this has on the ecosystem as a whole and being driven more strongly by prey birthing patterns~~. W~~Among w~~hite-faced capuchin monkeys at Santa Rosa show increased vertebrate predation rates towards the dry season compared to the wet season~~,~~, a period which coincides with some prey species' birthing season [28], as well as lower fruit availability and overall food abundance [31]. However, predation rates over some prey species, like birds, do not appear to follow seasonal trends [31]. ~~found a dry season~~

~~bias for the hunting of vertebrates, noting that this coincided with the period when several common prey species produced the majority of their young. Similarly, (37) found that overall predation by the Santa Rosa white-faced capuchin monkeys was biased towards the dry season, with squirrel predation in particular strongly following this pattern. This was attributed to the main mating and birth periods for squirrels occurring in the dry season, in combination with other factors such as lower fruit availability and overall lower food abundance. However, bird predation was less variable across the seasons, showing a slight trend in the opposite direction (37).~~

In addition, capuchin monkeys appear to be highly socially tolerant in feeding and foraging activities [46,47,65]. Food transfer, where the original hunter does not monopolize the vertebrate prey and parts/all of it are passed to others either passively or actively [40], has been recorded in bearded capuchins from Serra da Capivara National Park [38] and Serra das Almas Private Reserve of Natural Heritage [41] in Brazil, as well as brown [40] and white-faced capuchins in other sites [31], and some chimpanzee populations [66,67]. Understanding the extent to which this behaviour is ubiquitous across capuchin populations requires information from more sites in these taxa's distribution.

M~~As the above review of the literature demonstrates, m~~uch of our understanding of ~~what we know of~~ capuchin monkey vertebrate consumption stems from studies of gracile capuchins (genus *Cebus*) [28,31,43,48]. Less ~~research~~ focus has been given to vertebrate predation behaviour in robust capuchin monkeys (genus *Sapajus*), and, until recently, a large part of the available evidence on the subject was anecdotal and collected across short periods of times [37,41,44,47,51–53]. However, recent field studies are beginning to overcome this bias. In a two-year-long study in the dry Caatinga habitat of

Serra da Capivara National Park in Brazil, predation events by wild bearded capuchin monkeys (*Sapajus libidinosus*) were found to be relatively common with an overall rate of 4.6 events/100h [38], similar to those reported for the gracile white-faced capuchins of Santa Rosa, Costa Rica [28,31]. Furthermore, this Caatinga population is known to use probe tools to aid predation of small vertebrates, including lizards and snakes, followed by birds and rodents, but also larger prey, such as rock cavies (*Kerodon rupestris*), though only unsuccessful attempts have been observed for the latter [38,68]. ~~Although rare, bats were also observed being preyed on by these monkeys.~~

With the aim of further expanding our understanding of the contribution of animal resources to robust capuchins' diet, we collected data on the vertebrate prey accessed by two sympatric groups of wild bearded capuchin monkeys (*S. libidinosus*; classified as Near Threatened by the IUCN Red List [73]) from Fazenda Boa Vista, north-eastern Brazil, (one of which was slightly provisioned daily during the dry season) across 45 months (May 2006–December 2010). Specifically, we described the type of prey captured, determined the rate of vertebrate predation in each group, and evaluated the effect of ~~group,~~ food provisioning, sex, age class and non-vertebrate food availability~~environmental factors~~ (~~rainfall, temperature, humidity,~~ fruit and insects~~t~~ ~~availability~~) over predation rates. Additionally, to contribute to the understanding of whether this food transfer behaviours are ubiquitous in bearded wild capuchin populations, we also studied hunting and food transfer events in the Fazenda Boa Vista capuchins, noting the number and identity of individuals involved in each hunting event and in consuming the captured prey.

Formatted: Font: Not Italic

~~We also evaluated whether observed rates of vertebrate consumption in this population relate to body mass, energetic need, experience, protein source availability, necessity, or opportunism testing the following~~

In particular, we tested the following hypotheses:

1. Sexual biases in predation rates:

Formatted: Indent: First line: 0"

~~1. Body Mass:~~

1.1 Since different body mass among individuals of a given species demand different caloric intake [62,69], and the body mass of adult male capuchins is on average 60% larger than adult females [63], we predicted that adult male capuchins would engage in more vertebrate consumption events than adult females.

1.2

~~2. Energetic Need. If vertebrate consumption is mainly an energy source for capuchins:~~

~~2.1 S~~Alternatively, since greater energetic needs are placed on adult females compared to adult males by reproduction and lactation [64], adult females will engage in more vertebrate consumption events than adult males.

2.~~2~~ Effect of provisioning on predation rates:

The provisioned group has a higher net caloric intake than the unprovisioned group, as they have increased food availability and decreased food search costs. Therefore, the unprovisioned group will have greater need for vertebrate prey as a source of calories, and their vertebrate predation rates will be higher.

3. Age biases in predation rates:

~~2.~~3.1 Since the metabolism of juveniles expends more energy than the metabolism of adults, juvenile vertebrate predation rates will be higher than in adults.

~~2.4. Vertebrate prey replaces other high-calory food sources that are less costly to obtain, like fruits, during periods of scarcity. Therefore, vertebrate predation rates will increase when fruit availability decreases.~~

3.2 ~~Experience:~~ Alternatively, due to the difficulties of hunting vertebrate~~fast-moving, evasive~~ prey, ~~the vertebrates consumed by~~ adults, who have more hunting experience, will show higher vertebrate predation rates than ~~be larger in size (i.e., mammals) than those consumed by~~ juveniles and infants, who have less hunting experience ~~(e.g., lizards)~~.

4. Temporal variability in alternative food availability:

4.1 V~~Protein-source availability: v~~ertebrate prey replaces other high-protein food items that are less costly to obtain, such as insects, during periods of scarcity (e.g., during the dry season [70]). Therefore, vertebrate predation rates will increase during insect-poor periods.

4.2 Vertebrate prey replaces other high-calorie food sources that are less costly to obtain, like fruits, during periods of scarcity. Therefore, vertebrate predation rates will increase when fruit availability decreases.

5. Environmental effects over predation rates:

5.1 Environmental factors (rainfall, humidity and temperature) will not affect capuchin vertebrate predation rates. ~~Opportunity: since capuchin monkeys are~~

Formatted: Indent: Left: 0"

Formatted: Indent: Left: 0.5"

Formatted: Not Highlight

Formatted: Not Highlight

opportunistic foragers, vertebrate consumption will occur occasionally and predation rates will not be influenced by environmental factors (rainfall, temperature and humidity).

5.2 Alternatively, environmental factors (rainfall, humidity and temperature) will have an effect on capuchin vertebrate predation rates.

6. Food transfer:

Given capuchin monkeys' social tolerance in feeding and foraging activities [46,47,65] and previous reports of food transfer events in bearded capuchins [38,41], food transfer events will be observed in the study population.

6. Necessity: vertebrate consumption will be influenced by environmental factors (rainfall, temperature and humidity), and thus related to necessity rather than opportunism.

At Taï National Park, wild chimpanzees often hunt together in a cooperative way and the meat obtained is consumed by several individuals, some of which had not participated in the hunt (24, 72). Capuchin monkeys appear to be highly socially tolerant in feeding and foraging activities (e.g., 50, 51, 74). Food transfer, where the original hunter does not monopolize the vertebrate prey and parts/all of it are passed to others either passively or actively (44), has been recorded in bearded capuchins from Serra da Capivara National Park (42) and Serra das Almas Private Reserve of Natural Heritage (45) in Brazil, as well as brown (44) and white-faced capuchins in other sites (37) and some chimpanzee populations (24, 72).

To contribute to the understanding of whether this behavior is ubiquitous inbetter understand whether collective hunting occurs in bearded wild capuchin populationss, we

also studied hunting and food transfer events in the and if food transfer is also observed in the bearded capuchins at Fazenda Boa Vista, notingwe noted the number and identity of individuals involved in each hunting event and in consuming the captured prey. We expected capuchins to be able to act in parallel for a common goal and succeed without necessarily coordinating their actions, as described for cooperative tasks in the laboratory (73). GMoreover, given capuchin monkeys' social tolerance in feeding and foraging activities (e.g., 50, 51, 74) and previous reports of food transfer eventsthis behaviour in bearded capuchins (42, 45), food transfer will was expected to occur in the study population. (42, 44, 50, 51, 74-76).

Materials and mMethods

Formatted: Font color: Auto

Site

This study took place in Fazenda Boa Vista (hereafter, FBV; 9º 39' 36'' S, 45º 25' 10'' W), a privately owned area located in the northeastern Brazilian state of Piauí, 21 km northwest of the town of Gilbués. FBV is a transition zone between the Brazilian biomes *Cerrado* (savannah) and *Caatinga* (semiarid).. It consists of a sandy plain surrounded by 20-100 meter cliffs and interspersed by sandstone ridges and plateaus [71]. The majority of the plain is characterised by a high abundance of palms and medium-height trees, whereas the lowest part of the plain is a marsh conformed of gallery forest vegetation [71]. Meanwhile, the cliffs are dominated by shrubs and small trees, and the sandstone plateaus by low herbaceous vegetation [71]. The climate is characterized by marked rainfall seasonality which influences the availability of water in the ephemeral watercourses of the region and allows the distinction of two climatic seasons: a wet

season~~,~~ from October to April~~,~~ and a dry season~~,~~ from May to September [70]. While the annual average rainfall in this area is 1156 mm, only an average of 230 mm of rain falls during the dry season [71].

Subjects

Our subjects belong to two wild and sympatric groups of bearded capuchin monkeys (*Sapajus libidinosus*), one of which was attracted to visit a study area by provisioning it daily with nuts, corn and water ~~provisioned daily~~ during ~~the dry season in~~ the study period [70]. ~~Both the unprovisioned group (composed by 8–16 individuals) and the provisioned group (composed by 17–19 individuals) have been continuously observed since 2005 as part of the Etho*Cebus* long-term research project on behaviour and ecology of wild bearded capuchin monkeys [72].~~ O~~The provisioned group was attracted to visit a study area by provisioning it daily with received nuts, corn and water; o~~n average, each individual of the provisioned group received 825 kJ every day [70]. Both the unprovisioned group (composed by 8–16 individuals) and the provisioned group (composed by 17–19 individuals) have been continuously observed since 2005 as part of the Etho*Cebus* long-term research project on behaviour and ecology of wild bearded capuchin monkeys [72], and were thus well habituated to human presence. ~~*S. libidinosus* is classified as Near Threatened by the IUCN Red List [73].~~

Formatted: Font: Italic

Observations

Observations were carried out between May 2006 and December 2010 (Table 2). The overall observations of the unprovisioned group accounted for 3012 hours (distributed across 43 months across wet and dry seasons) and those of the provisioned group accounted for 2812 hours (distributed across 45 months across wet and dry seasons)

(Table 2). Data on vertebrate consumption events were collected following all occurrence sampling [74] and group scan sampling methods [75]. Since the sampling interval for collection of group scan activity budgets was different among observers, we divided the data collection into two study periods, S1 and S2, corresponding to the length of interval used.

In S1 (May 2006 to April 2008), we recorded data on activity budgets using a 10 min group scan sampling method at 10 min intervals, for a total of 84,470 scans (provisioned group: n=42,917; unprovisioned group: n=41,553). In S2 (April 2009 to December 2010), we recorded activity budgets using 1 min group scan sampling at 5 min intervals for a total of 75,028 scans (provisioned group: n=62,588; unprovisioned group: n=12,440). To test whether differences in sampling interval between study periods (S1 and S2) affected our results, we ran a generalized linear mixed model (GLMM) with a Poisson error structure. The response variable was the number of scans per month in which vertebrate consumption was observed, with study period (S1 vs S2) included as a fixed effect and total monthly scans as a covariate.We ran a GLMM to test the effect of the difference in sampling interval length across S1 and S2. Since we did not find a significant effect of study period in this modelacross S1 and S2 and the total monthly scans (χ^2=1.065, df=1, p=0.302), we considered all data collected by scan sampling during both study periods together in further analysis. Specifically, we focus on those scans in which each group/individual was observed consuming vertebrates.

Table 2. Observation and demographic details for the two groups of capuchin monkeys studied at Fazenda Boa Vista: dates of observation periods, number of observation hours, study group (CH: Chicão, provisioned group; ZA: Zangado, unprovisioned group), group size, sex composition (♂: Males; ♀: Females), and age class composition (A: Adults, J: Juveniles, I: Infants).

Observation period[a]	Observation hours	Study group	Group size[b]	♂	♀	A	J	I
June 2006 - May 2007[1]	706	CH	12-18	11	7	11	5	2
	349	ZA	8-14	7	7	8	4	2
May 2006 - April 2008[2]	775	CH	17-19	13	8	12	4	5
	1932	ZA	8-14	7	8	9	4	2
Jan. 2009 - Dec. 2010[3]	1331	CH	17-19	11	10	9	8	5
	733	ZA	13-16	12	7	8	6	6

[a]Data sources: [1](81); [2](82); [3](83)

[b] Group size differed across observation periods due to births/deaths/migration.

A *Consumption Event* was defined as an individual observed taking and at least partially consuming any vertebrate item. WFollowing the methodology of (37), we considered nestlings and eggs as vertebrate consumption [31]. We also scored which individual(s) captured the prey and which individual(s) ate it, and described which body parts were eaten. Hunting events were labelled Type I when one individual hunted the prey and Type II when more than one individual hunted the same prey simultaneously. Events in which food-transfer occurred were classified byas in (44), referring to the individual(s) initially possessing the food item as *possessor(s)*, and the individual(s) to whom the transfer is directed as *recipient(s)* [40].

Formatted: Font: Not Italic

Data analysis: statistical modelling

We modelled the rate of predation events per unit of observation time as a function of the following fixed effect variables (model specified in eq. 1):

(1) Rainfall (mm). Recorded at the field station (Hygro-Thermometer Clock model 445702′, EXTECH instruments) daily.

(2) Temperature (ºC). Daily maximum and minimum temperatures were recorded at the field station (Hygro-Thermometer Clock model 445702′, EXTECH instruments).

(3) Humidity (%): Daily minimum and maximum recorded at the field station (Hygro-Thermometer Clock model 445702′, EXTECH instruments).

(4) Fruit availability. Estimated by combining:

(a) Stratified random sampling method, whereby 100 fruit traps were placed at 30 m intervals, 1 m to the side of a 3 km trail crossing the different physiognomies of the study area. At two-week intervals, fallen material in the traps was quantified and weighed *in situ* and then returned to the same location, so fruits were not removed from their natural environment.~~, with collection and weighing of contents every 2 weeks~~. Food availability index (FAI; kg/ha) was estimated based on the dry weight of the material collected from traps and on the cumulative surface area.

(b) Monthly direct observation and recording of presence/absence of fruit in 254 palm trees of the species catulé~~è,~~ (*Attalea barreirensis*) (N = 136)~~,~~ and piassava~~,~~ (*Orbignya* sp.) (N = 118), in the same 3 km trail. This data was used to estimate two monthly FAI, one expressed as the number~~as number~~ of palms with fruits/ha and one for total nuts, as the sum of catulé~~è~~ and piassava palms with fruits/ha.

Formatted: Font: Italic

Formatted: Font: Not Italic

(5) Insect availability. Estimated through stratified random sampling method, whereby 100 pitfall~~fruit~~ traps were placed at 30 m intervals, 1 m to the side of the same 3 km trail ~~as~~ used to estimate fruit availability.~~,~~ At two-week intervals, fallen material in the traps was quantified and weighed in situ and then returned to the same location, so material was not removed from its natural environment.~~ with collection and weighing of contents every 2 weeks.~~ Food availability index (FAI; kg/ha) was estimated based on the dry weight of the material collected from traps and on the cumulative surface area [70].

(6) Sex.

(7) Age.

(8) Group (unprovisioned vs. provisioned).

Correlations among predictor variables were assessed using Spearman's rank correlation coefficient (ρ) and variance inflation factors (VIFs) (S1 Table). Spearman's ρ indicated only low to moderate pairwise associations, and all VIF values were below 1.25, suggesting negligible multicollinearity. The strongest association was a moderate correlation between insect availability and rainfall (ρ=0.45). However, low VIF values indicated that the model was still able to distinguish between the seasonal increase in insect availability and the direct effect of rainfall on capuchin predation activity. In addition, model comparison based on Akaike Information Criterion (AIC) [76] values showed that models including both environmental variables (rainfall, temperature, and humidity) and food availability variables (fruit and insect availability) consistently outperformed models including environmental variables alone.

Our data set had many observations with 0 predation events documented. Since count data with many zero values cannot be transformed to normal [77], we implemented a predictive model capable of directly estimating the predation events observed. This predictive model also deals with excesses of zeros, and with non-linearity, non-homogeneity of variance and an outcome variable bounded at zero [78]. Excess zeros arise from low frequency of occurrences in behavioural studies and are thus influenced by an array of life-history processes and the ecological context. We can also have false zeros, that is when occurrences are happening, but not during the surveying period, or when occurrences happen during surveying but are not detected [79,80]. When working with longitudinal data (characterized by repeated measurements from individuals within and across units of time), it is necessary to explicitly model the random effects structure. This allows for a correct inference of the fixed effects, accounting for non-independence among observations and avoiding pseudoreplication [81,82]. Thus, we relied on zero-inflated generalized linear mixed models (ziglmm) to perform the analysis, more specifically a zero-inflated negative binomial (zinb) algorithm with crossed random effects. To standardize for observation effort, we included the log of the number of scan samples as an offset term (*logtime*), so that the model estimates predation rates per unit observation effort rather than raw counts. Therefore, our model was defined as follows:

$$\begin{gathered} Predation\ Events \sim offset(logtime) + Sex + Age + Group + Rainfall + \\ Fruit + Insect + (1|ID) + (1|Month) \\ Zero\ Inflation \sim Humidity + Temperature \end{gathered} \tag{1}$$

where the random effects of individual ID and month (1-45) are expressed in parentheses.

The negative binomial distribution is technically Poisson-distributed with underlying gamma-distributed heterogeneity [83]. So, it is better suited than a typical Poisson

regression to account for overdispersion [84]. All parameters were fitted in R with the glmmTMB package, using maximum likelihood estimation, except for random effects which were estimated via a Laplace approximation [85–87]. After running many different model architectures, we used information-theory for multi-model inference to select the final reduced model. Notice that some architectures could not be modelled because of convergence problems while estimating parameters (e.g., non-positive-definite Hessian matrix; extreme or very small eigenvalues detected; and/or singular convergence). We selected as the final model (eq. 1) the one with the lowest Akaike Information Criterion (AIC) value, as this metric optimises the trade-off between fit and complexity [76,82,88].

The specified model (eq. 1) was applied to all collected data on vertebrate predation (Model 1), as well as two subsets of it: predation events involving mammal prey only (Model 2) and predation events involving non-mammal prey only (Model 3). We refer to non-mammalian prey as Sauria, a clade composed of Lepidosauromorpha (snakes, lizards and rhynchocephalians) and Archosauromorpha (birds and crocodilians) [55]. Models 2 and 3 were applied to compare patterns of mammal vs. saurian predation in the studied populations.

Additionally, we also used Non-parametric tests (Chi-square tests) to assess whether hunting events were more frequently performed by one (Type I) or more individuals (Type II) and whether consumption was more frequently performed by one or more individuals. Statistical significance level was set at α = 0.05 for all of the analyses performed.

Ethical nNote

The research adhered to the Brazilian government permit (SisBio #28689) in accordance with the Brazilian legislation (law #11.794, October 8, 2008) and to the Brazilian National Council for Scientific and Technological Development (CNPq) authorization for Scientific expedition, under the Project CMC 000111, which included the permission from the landowners Marino Gomes de Oliveira and Maria Fonseca Gomes de Oliveira to conduct research in their field site. We adhered to the American Society of Primatologists' principles for the ethical treatment of primates. The research adhered to the Brazilian government permit (SisBio # 28689) in accordance with the Brazilian legislation (law #11.794, October 8, 2008) and adhered to the American Society of Primatologists' principles for the ethical treatment of primates.

Inclusivity in global research

Additional information regarding the ethical, cultural, and scientific considerations specific to inclusivity in global research is included in the Supporting Information (S1 Checklist).

Results

Each group of capuchins was followed from dawn to dusk. During a total of 5,093 observation hours we obtained a total of 446 events of vertebrate consumption (both when in progress or about to begin). Of the 446 events, 281 were recorded by scan sampling and 165 were recorded by all occurrence sampling. The provisioned group accounted for 212 events, and the unprovisioned group for 234 events. We recorded 8 events of vertebrate consumption by infants (3 lizards, 2 rodents, 2 snakeserpents, 1 undetermined

Formatted: Font: 12 pt, Not Bold

Formatted: Font: 16 pt, Bold

Formatted: Space After: 0 pt, Keep with next, Keep lines together

prey). For the modelling analyses only the scan sampling events were used. We obtained an overall rate of 6.34 predation events/100h.

Consumed p~~P~~rey i~~I~~tems

Similarly to what has been observed elsewhere [40,44,47], the most common prey of capuchins in FBV are vertebrates of small dimensions like lizards and rodents. The major prey types were diurnal and abundant (pers. com.) reptiles (n=136: lizards, geckos, snakes, and iguanas), mammals (n=97: primarily rodents, but also opossums, and two bats) and birds (n=34: especially nestlings and eggs) (Table 3; see ~~Video~~ S1 Video).

Table 3. Number of prey items consumed in the 281 events of vertebrate consumption recorded by scan sampling in the provisioned and unprovisioned groups. Birds include both nestling and eggs. Und. = Undetermined.

**Mammals**			**Saurian**				**Und.**
Rodents	Marsupials	Bats	Lizards	Birds	Snakes	Iguana	
80	15	2	123	34	12	1	14
28.5%	5.3%	0.7%	43.8%	12.1%	4.3%	0.4%	5.0%

Avian prey were usually eaten (Fig. 1A). When this was not the case, the monkeys preferred to eat the brain and viscera. For mammalian prey the monkeys preferred to eat the tail and paws, and in some events the eyes as well. The skin covering the forehead and the back of the prey was ripped off in a few cases (Fig. 1B). For geckos and lizards, capuchins frequently ate feet, tail, eyes, brain, skin and, in some cases, viscera (Fig. 1C).

Only male capuchin monkeys were observed consuming snakes. When eating snakes (Fig. 1D), they often discarded the head and ate the rest of the body, especially the internal organs. In general, muscular tissue and associated ligaments and tendons were torn apart in small bites, repeatedly masticated and swallowed; tender parts of the prey, such as the viscera and brain, were ingested more rapidly. We never observed meat consumption accompanied by leaf ingestion.

Due to limited numbers of observations in certain categories (e.g., 2 bats, 12 snakes, etc.), we concatenated small prey subsets, enabling statistical analyses and modelling. For example, alongside a global model, we compared mammals versus saurians~~non-mammals~~ using two distinct sub-models.

Fig. 1. Evidence of vertebrate consumption at FBV. A (top left): Adult male feeding on avian prey (photo by Luciano Candisani); B (top right): Mammal prey (marsupial) preyed upon by an adult male. The skin was ripped off and the viscera exposed (photo by Noemi Spagnoletti); C (bottom left): Lizard preyed upon by an adult female. Viscera were exposed (photo by Eduardo D. R. Silva); D (bottom right): Snake preyed upon by an unidentified individual. The upper part of the snake containing the head was ripped off and the viscera exposed (photo by Noemi Spagnoletti).

Model 1: Global model for v~~V~~ertebrate c~~C~~onsumption

Individuals in the provisioned group consumed significantly less vertebrate prey than those in the unprovisioned group (ZA; $p < 0.001$; Table 4; Fig. 2). The estimated rate of predation events per unit of observation time in the non-provisioned group was 1.88

times higher than in the provisioned group.~~The estimated rate of predation events per unit observation time in the non-provisioned group was 1.88 times that of the provisioned group.~~ Infants consumed vertebrate prey at only one-fifth the rate of adults ($p < 0.002$), whereas juveniles did not differ significantly from adults (Fig. 3~~2~~). Males showed higher rates of vertebrate consumption than females, with an estimated rate ratio of 1.4 (p = 0.049).

Table 4. Results of the zero-inflated negative binomial generalized linear mixed model testing predictors of vertebrate predation rate per unit of observation time.~~Vertebrate predation model.~~

**Term**	**Estimate**	**SE**	**Lower CL**	**Upper CL**	**Wald's**	***p*-value**
Conditional model (Negative Binomial)						
(Intercept)	-6.6802	0.199334	-7.07089	-6.28951	-33.5126	-
Sex (Male)	0.348003	0.176809	0.001463	0.694543	1.968241	0.04904*
Age (Juvenile)	-0.17863	0.174004	-0.51967	0.162411	-1.02659	0.304616
Age (Infant)	-1.48032	0.468656	-2.39886	-0.56177	-3.15864	0.001585*
Group (ZA)	0.632806	0.183204	0.273734	0.991879	3.454114	0.000552*
Rainfall	0.428193	0.169339	0.096294	0.760092	2.52861	0.011452*
Fruits	-0.49586	0.146094	-0.7822	-0.20952	-3.39411	0.000689*
Insects	-0.23761	0.14441	-0.52065	0.045427	-1.64539	0.099889
Zero Inflation model						
(Intercept)	25.13092	16.93205	-8.05529	58.31714	1.484222	-

Temperature	-2.11166	0.858397	-3.79409	-0.42924	-2.46001	0.013893*
Humidity	0.377545	0.130974	0.120841	0.634249	2.882603	0.003944*

Parameter estimates are shown for the conditional and zero-inflation components. Observation effort was accounted for by including log(number of scan samples) as an offset term. *n* = 1092. Random effects: ID (36); Month (45). Final model metrics: AIC = 1253.7; BIC = 1323.6; logLik = -612.8; deviance = 1225.7; df residuals = 1078. ZA: unprovisioned group. Asterisks indicate $p < 0.05$. Reference categories: Sex: female; Age: adult; Group: provisioned.

Formatted: Font: 11 pt

Formatted: Font: Not Italic

Fig. 2. Dot-whisker plot showing the incidence rate estimates for predation events based on all variables in the model.

Formatted: Font: 12 pt, Bold, Not Italic

Formatted: Font: 12 pt, Not Italic

Fig. 3~~2~~. The likelihood of occurrence of a predation event by group (CH: provisioned; ZA: unprovisioned), age class, and sex.

Rainfall positively affected the rate of predation events per unit observation time (*p* = 0.011; Fig. 4~~3~~). In contrast, fruit availability showed a strong negative association with predation rates (*p* < 0.001; Fig. 5~~4~~). Insect availability was not significantly associated with predation rate (*p* = 0.1).

Temperature (*P* = 0.014) and humidity (*P* = 0.004) were used in a separate part of the model—the zero-inflation model.~~—since this architecture substantially improved results in ΔAIC). These variables played a critical role in determining the likelihood of observing zero predation events for a particular individual in a given month Table 4, "Zero Inflation~~

model". Temperature had a significant negative effect (p = 0.014): higher temperatures reduced the probability that an observation fell into the "always zero" state, making predation events more likely to be recorded. In contrast, humidity had a significant positive effect (p = 0.004): higher humidity increased the probability of an excess zero, i.e., conditions under which no predation events were observed. Within the negative binomial framework, the exponents of estimates serve as the incidence rate ratio, providing valuable insights into the impact of variables in the model (Fig. 5).

Fig. 43. Effect of rainfall (mm) on the likelihood of a capuchin monkey preying on a vertebrate. Adjusted for variables kept fixed as logtime = 4.74; Age Class = Adult; Fruit availability = 289.89; Insect availability = 0; Temperature = 0, Humidity = 0.

Fig. 54. Effect of fruit availability (kg/ha) on capuchin vertebrate predation rate. Adjusted for variables kept fixed as logtime = 4.74; Age Class = Adult; Rainfall = 94.99; Insect availability = 0; Temperature = 0, Humidity = 0.

Fig. 25. Dot-whisker plot showing the incidence rate estimates for predation events based on all variables in the model.

Model 2: Sub-model of mammal consumption

The primary focus of this analysis was to model predation events involving mammals, namely rodents, marsupials, and bats (~~Table ~~S2~~1~~ Table; Fig. 6a). We again employed a negative binomial mixed-effects model with a zero-inflation component to address overdispersion and excess zeros in the data, respectively. The model exhibited good fit statistics, with an AIC of 502.6, and several predictors showed significant associations with variation in mammalian predation rates.

Males showed significantly higher mammal predation rates than females (p = 0.006) (~~Table ~~S2~~1~~ Table). Age was not a statistically significant predictor of mammal predation rate. The difference between the provisioned and unprovisioned groups was also not significant. Increased rainfall (p = 0.029) and higher fruit availability (p < 0.001) were both significantly associated with higher and lower predation rates, respectively. Temperature significantly reduced the probability of excess zeros (p = 0.021), indicating that predation events were more likely to be observed at higher temperatures, while humidity showed a non-significant trend.

Model 3: Sub-model of s~~S~~aurian predation

The same zero-inflated negative binomial regression model was employed to investigate the relationship between saurian predation events and group, individual and environment-related predictors (~~Table ~~S3~~2~~ Table; Fig. 6b). Saurian predation showed a significant positive association with group (p = 0.004), with the unprovisioned group showing a higher saurian predation rate than the provisioned one. We did not find significant relationships between any environmental variable and predation rate. We also did not find a significant effect of sex or age over predation rate. Overall, our results suggest that saurian~~non-mammalian~~ predation events are most strongly influenced by provisioning.

Fig. 6. Contrasting sub-models with predation rate split by prey type: a) mammals; b) saurians. ZA: unprovisioned group.

Hunting and food transfer events

Hunting events were rarely observed. Out of 280 consumption events recorded by scan sampling for both groups, we observed hunting preceding consumption in 51 instances (see ~~Video~~ S2~~3~~ Video). In all other cases, hunting was noticed as the prey was grasped, or inferred to have occurred from the presence of prey seen in a capuchin's hand (see ~~Video~~ S3~~2~~ Video). Consumption of vertebrate prey was significantly higher in type I events (individual, 82.14%, N = 230) than in type II events (collective, 17.86% N = 50) Chi-square, χ^2= 17,708, df=2, *p*=0.000). Of the 165 vertebrate predation episodes recorded by all occurrence sampling for both groups, 81.21% (n=134) were episodes in which only one individual was seen consuming the prey item, whereas 18.87% (n=31) were episodes in which two or more individuals were seen consuming the same prey item. The transfer of meat, or of other parts of the carcass (such as skin, bones, etc.) was observed in 32 of the 50 type II events (64%).

Discussion

We reported on the most extensive record of bearded capuchin (*S. libidinosus*) vertebrate predation currently available: 45 months of vertebrate consumption observations across two sympatric groups in Fazenda Boa Vista (Brazil). We showed that vertebrate consumption is habitual in these groups (*sensu* McGrew, 1992 [89]) and occurs at an average rate of 6.34 predation events per 100 hours. This behaviour was seen across

all age classes (adults, juveniles and infants) and both sexes. Like bearded capuchins in other areas of Brazil (e.g., Serra da Capivara National Park [38]; Serra das Almas Private Reserve of Natural Heritage [41], and in support of other observations of the Fazenda Boa Vista capuchins [39]), both groups studied here were observed feed on rodents, bats, lizards, adult birds and snakes. For the first time in bearded capuchins, we also recorded opossums, iguanas and bird eggs/nestlings as part of this species' diet. Additionally, we observed both hunting events with more than one participating individual, as well as food transference events.

Our results revealed a strong association between food availability and the exploitation of small animal resources (see also Table 5 below). First, provisioned individuals exhibited lower vertebrate predation rates than unprovisioned ones. This effect was upheld when looking only at saurian prey, but not when looking only at mammalian prey. Provisioning has been shown to alter primates' activity budgets, diets and ranging patterns by providing resources of a higher caloric content, availability and predictability than those found in their natural habitat [90,91]. Asquith (1989) [92] raised concerns about the lack of comparative data between provisioned and unprovisioned individuals in primate studies. Our research directly addresses this issue by using a quasi-experimental approach comparing the predation patterns of provisioned and unprovisioned groups of sympatric capuchins. Future studies on wild primate dietary patterns must strongly consider the potential influence of planned or unplanned (e.g., crop raiding, feeding on garbage) provisioning on predatory behaviours, as our results suggest primates may be less inclined to access animal foods when provisioned.

Table 5. Factors considered in this study, their prediction(s) in terms of vertebrate consumption, and whether results support each of them.

**Category**	**Predictions on vertebrate predation rate (VPR)**	**Prediction supported?**
1. Sexual biases in predation rates.~~Body mass~~	1.1 VPR in adult males > adult females.	Yes.
	1.2 VPR in adult males < adult females.	No.
2. Effect of provisioning on predation rates.~~Energetic need~~	VPR in unprovisioned group > provisioned group. ~~2.1 VPR in adult females > adult males~~	Yes. ~~No~~
	~~2.2 VPR in unprovisioned group > provisioned group~~	~~Yes~~
3. Age biases in predation rates.	~~2.~~3.1 VPR in juveniles > adults.	No.
	3.2~~2.4 Fruit availability negatively correlated~~ VPR in juveniles and infants < adults.	No for juveniles.<br>Yes for infants.~~Yes~~

Formatted: Font: Bold

Formatted Table

Formatted Table

4. Temporal variability in alternative food availability. ~~3. Experience~~	4.1 Insect availability is negatively correlated with VPR. ~~Adult VPR > juvenile VPR~~	No.
	4.2 Fruit availability is negatively correlated with VPR. ~~Adult VPR > Infant VPR~~	Yes.
~~4. Protein source availability~~	~~Insect availability negatively correlated VPR~~	~~No~~
~~Opportunism (Op)~~ 5. Environmental effects over predation rates.	~~Occasional and not influenced by rainfall and temperature (Op-1)~~ 5.1 Environmental factors (rainfall, humidity and temperature) will not have an effect on VPR.	~~Yes, for saurian consumption. No, for mammal consumption.~~ No.
	5.2 Environmental factors (rainfall, humidity and temperature) will affect VPR.	Yes.

Formatted Table

~~Necessity (N)~~<br>6. Food transfer.	~~Influenced by rainfall, temperature, and fruit availability (N-1)~~<br>Food transfer events will be observed.	~~Yes, for saurian consumption.~~<br>~~No, for mammal consumption.~~<br>Yes.

Second, we found vertebrate meat intake varies inversely with fruit abundance, a trend upheld in the mammal-only model but not in the s~~S~~aurian-only model. This suggests small mammals may be important sources of energy in wild bearded capuchins at Fazenda Boa Vista, and may replace other sources of calories (i.e., fruits) during periods of scarcity. This aligns with the "energy shortfall hypothesis", which posits that animal-source foods are a particularly important source of energy for primates when plant-source foods become less available [1]. A similar mechanism has been proposed as a driver of increased meat consumption in the genus *Homo* [13]: as late Pliocene and early Pleistocene eastern African hominin environments became increasingly seasonally arid [13,58,93], the availability of plant-source foods that are key to many primates' diet in stable, humid, forested habitats would have only been available intermittently, whereas animal resources (flesh and in-bone nutrients like marrow and brain) would have remained accessible throughout the year, thus selecting for a higher frequency of predatory behaviours in our lineage [13]. Bearded capuchin reliance on small vertebrates during fruit-poor periods supports this strategy as a possible predecessor and/or complement to large-prey hunting in humans in seasonal environments, possibly along with opportunistic scavenging of within-bone nutrients [3]. Evidence from the fossil and

archeological record [15,16,19,20] and contemporary forager societies [17,18] indicates that such small-vertebrate exploitation has had a lasting presence in the human lineage.

However, the population of Fazenda Boa Vista appears to be an exception among extant primates: the most extensive review of animal predation across the primate order to date found little evidence in support of the energy shortfall hypothesis [1], even in species inhabiting seasonally extreme environments similar to those reconstructed for early Pleistocene hominins, like baboons [1] and savanna chimpanzees [1,94]. For instance, the hunting behaviours of chimpanzees in the savanna-woodlands of Fongoli are not explained by fruit scarcity [94]. Furthermore, chimpanzees in some forested environments appear to hunt more often during food-rich rather than food-poor seasons, possibly because they can afford the energetic losses of an unsuccessful hunt when they have another easily obtainable source of energy: fruit [1,59,95]. White-faced capuchins in Santa Rosa (Costa Rica) do consume more vertebrate prey in the dry season, when fruit is scarce, but this is likely due to the concurrent increase in preferred prey availability, as coati infants are born at this time [1,31]. Future research should aim to clarify whether the association between fruit abundance and vertebrate consumption in the Fazenda Boa Vista capuchins truly supports the energy shortfall hypothesis, or if the observed pattern is a result of opportunistic predation of seasonally abundant small mammals.

It is important to note that, in this study, we combined the availability of palm nuts with that of other fruits. The kernels of palm nuts constitute a key food item for the capuchins at Fazenda Boa Vista: they are rich in lipids and protein, and ~~— because they are obtained with the aid of tools —~~ provide a reliable source of energy and a stable protein intake [96]. It is therefore possible that the observed inverse relationship between vertebrate predation and fruit availability is strongly influenced by fluctuations in nut

consumption and availability. This requires further testing. Additionally, it is possible that the measure of fruit availability used in the present study does not reflect variation in capuchin fruit consumption (e.g., capuchins may consistently access some fruits regardless of their availability, or they may not increase their consumption of non-preferred fruits despite their increased availability). Therefore, future studies should aim to directly quantify temporal variations in fruit consumption in capuchins and test their effect on vertebrate consumption rates.

Environmental factors also had a significant effect over capuchin vertebrate consumption patterns. Increased rainfall was linked to increased vertebrate consumption rates in this population. Furthermore, predation events were more likely to be recorded at higher temperatures and less likely to be recorded at higher humidity levels. Rather than reflecting a direct effect of these environmental variables over vertebrate predation, our results likely reflect the presence of a mediating variable: the availability of preferred vertebrate prey species. It is possible that periods of higher temperatures and rainfall, as well as lower humidity, coincide with the birthing season of some prey species. These seasons have been linked with increased vertebrate predation rates in other capuchin populations, as they are characterized by more readily abundant and easier-to-catch prey: infants [31]. Future work should aim to disentangle these effects by directly measuring the effects of vertebrate prey abundance over capuchin vertebrate predation rates.

We also found strong effects of age and sex on capuchin vertebrate consumption rates. Across both study groups, infants–but not juveniles–had lower vertebrate consumption rates than adults, and males had higher predation rates than females. These sex differences are similar to those documented at other long-term capuchin monkey field sites [28,31,38,40,58], as well as in baboons [97–102] and chimpanzees [1]. Improving our understanding of these patterns in robust capuchins allows us insights beyond those

provided by chimpanzees, baboons and gracile capuchins, on whom much of the research on the subject has focused. That males appear to capture more vertebrates than females across all the aforementioned primate taxa presents an interesting pattern, especially considering ongoing debates about sex biases in hunting among modern unindustrialized societies [103,104]. Across many of these communities, men hunt more often than women on average [104–107]. Although there is variation between societies and women regularly hunt in many [103,108–111] these sex differences appear to be present and accurately reported [104].

Aside from increased caloric intake demanded by male bearded capuchin's body size compared to that of females [58,62], the observed sex differences in predation rates may be explained by a risk-averse foraging strategy in females compared to males [61]. For instance, all snake predation events were performed by males in our study population, which may reflect female avoidance of potentially dangerous prey. In chimpanzees, females feed significantly more on insects, such as termites and ants, than males [112,113]. This has been proposed as a strategy that combines longer investment of time to access high quantities of protein and calories from sources (A) more reliable and less energetically costly than vertebrate hunting, which has an important percentage of failure, (B) under less competition, as invertebrates are more widely available than vertebrates and males seek them less, and (C) that pose a lower risk of injury to females and infants, as well as of losing prey to males, than hunting [112–114]. Gilby *et al*., (2017) [112] posited this strategy might have been present in the last common ancestor between the *Pan* lineage, to which chimpanzees belong, and the human lineage, hypothesizing sex differences in hunting behaviour would have preceded the appearance of the sexual division of labor among modern humans. In unindustrialized societies today, female risk

aversion has also been posited as a driver of the sexual division of labor [115]. By extending the comparison beyond chimpanzees and humans, capuchins may provide insights on the extent to which whether sex biases in vertebrate predation may be a general response to the risks and energetic trade-offs of hunting. Like chimpanzees, female white-faced capuchins tend to consume more invertebrates than males [58] and insects are a vital source of energy for them, particularly during lactation [27]. We did not evaluate differences in invertebrate predation rate between bearded capuchin males and females; future research should aim to bridge this gap. Convergent sex-biases in invertebrate and vertebrate access across distantly related primate taxa might suggest that female avoidance of high-risk prey may be a recurrent strategy among vertebrate-eating primates.

The majority of the hunting events we observed, as well as the consumption of captured prey, were carried out by one individual rather than by two or more. Instances of prey transfer did occur in our population, as in the bearded capuchins from Serra da Capivara National Park [38] and Serra das Almas Private Reserve of Natural Heritage [41]. However, many of the small vertebrates captured by a group of hunters were monopolized and consumed by only one individual. Factors such as dominance rank may influence who gains priority access to prey, as subordinate individuals may be supplanted [31], while kinship ties could play a role in determining whether prey is shared [116]. Nevertheless, most predation events were performed alone and in the absence of other group members, suggesting that collaborative hunting is not necessary for the capture of small prey in bearded capuchins. Instead, solitary hunting and eating may minimize intraspecific competition for limited food. Many small vertebrates may not provide enough nutrients, proteins and energy for more than one individual, potentially discouraging cooperative hunting and sharing. For example, larger prey like adult squirrels appeared to be shared by white-faced capuchins in Santa Rosa, whereas nestlings

were usually entirely eaten by one captor [31]. Similarly, smaller prey sizes have been reported to discourage cooperative hunting in chimpanzees [117]. Future research might look into whether prey size may be a predictor of the likelihood of group hunting and sharing in bearded capuchins.

Capuchin monkeys in the present study consumed vertebrate's brains and viscera first, and then (but not always) the rest of the body. Capuchins' preferential access of these tissues may respond to their high lipid (and protein, in the case of viscera like liver) content compared to other prey bodily tissues [118,119]. For example, chimpanzees at Gombe National Park consistently consume the brains of subadult prey and the viscera of adult prey, whose skulls are more robust and harder to crack, before moving to the rest of their body, possibly to maximize the fats and nutrients obtained before the carcass is stolen by conspecifics [120,121]. A similar consumption order has been observed in Taï [66] and Fongoli [122] chimpanzees. The scavenging of in-bone nutrients, such as those provided by the brain, have been posited as one of the stepping-stones towards the emergence of the human predatory pattern [3]. Like bearded capuchins and chimpanzees, hominins may also have preferentially accessed the brains of small prey, and later began scavenging these in-bone nutrients from large carcasses, as proposed by Thompson *et al.*, (2019)~~in~~ [3], before they were able to hunt prey larger than themselves. However, our results differ from those of [36] on the white-faced capuchins of Santa Rosa, Costa Rica, who appear to consume the viscera first and typically discard the head. Further research on the sequence in which different primates consume prey body parts will provide a more nuanced understanding of the factors shaping their contrasting prey-consumption strategies [120].

Formatted: Font: Italic

While primates accessing prey of the same size or larger than themselves is only seen in humans [3], the consumption of small vertebrates is seen across a large number of taxa [1]. On one hand, this may reflect an ancient opportunistic strategy for the obtention of energy, nutrients and/or proteins. On the other hand, small vertebrate predation may have emerged through convergent evolution in several branches of the primate order, as multiple species recurred to the same solution to address similar ecological challenges. Hunting small prey does not necessarily require tools or cooperation with group members to be successful, which may contribute to the persistent presence of this behaviour in the primate order [1], including in hominins [14,15,17,19,20]. Despite the emergence of the human predatory pattern, the consumption of small vertebrates has persisted in the human lineage, and small game forms part of the diet of many unindustrialized societies today [16,18]. The presence of this form of vertebrate consumption across primate taxa supports the possibility that small prey provides an important, reliable and less costly source of nutrients than larger prey, the capture of which has a very high rate of failure and requires larger energetic investments.

Future directions

We recommend that early faunal assemblages composed of small vertebrates should be re-examined closely for evidence of damage inflicted by predation. Future capuchin monkey studies investigating vertebrate consumption should record the order in which different parts of the animal are consumed and document the taphonomy of these processes, so that we may identify the traces that small vertebrate predation leaves behind after the animal is consumed and abandoned, and thus build a better picture of the signatures we should be searching for in the fossil record. This will allow us to identify

how patterns of consumption have changed through time in assemblages with hominins and other fossil primate presence.

Acknowledgements

We thank the Fonseca de Oliveira family for permission to work on their land, as well as the Etho*Cebus* Project team, supporting the logistics of the research. We are also grateful to our field assistants, Jozemar de Oliveira, Arisomar de Oliveira, Renato de Olivera, Marino Júnior and Marcos Fonseca de Oliveira.

References

1. Watts DP. Meat eating by nonhuman primates: A review and synthesis. J Hum Evol. 2020;149: 102882. doi:10.1016/j.jhevol.2020.102882

2. Butynski TM. Vertebrate predation by primates: a review of hunting patterns and prey. J Hum Evol. 1982;11: 421–430. doi:10.1016/S0047-2484(82)80095-X

3. Thompson JC, Carvalho S, Marean CW, Alemseged Z. Origins of the Human Predatory Pattern: The Transition to Large-Animal Exploitation by Early Hominins. Curr Anthropol. 2019;60: 1–23. doi:10.1086/701477

4. Aiello LC, Wheeler P. The Expensive-Tissue Hypothesis: The Brain and the Digestive System in Human and Primate Evolution. Curr Anthropol. 1995;36: 199–221. Available: https://www.jstor.org/stable/2744104

5. Aiello LC, Bates N, Joffe T. In defense of the Expensive Tissue Hypothesis. In: Falk D, Gibson KR, editors. Evolutionary Anatomy of the Primate Cerebral Cortex. Cambridge: Cambridge University Press; 2001. pp. 57–78. doi:10.1017/CBO9780511897085.006

6. Antón SC, Potts R, Aiello LC. Evolution of early Homo: An integrated biological perspective. Science. 2014;345: 1236828. doi:10.1126/science.1236828

7. Foley R. The Evolutionary Consequences of Increased Carnivory in Hominids. In: Stanford CB, Bunn HT, editors. Meat-Eating & Human Evolution. Oxford University Press; 2001. p. 0. doi:10.1093/oso/9780195131390.003.0016

8. Hagen EH, Blackwell AD, Lightner AD, Sullivan RJ. Homo medicus: The transition to meat eating increased pathogen pressure and the use of pharmacological plants in Homo. Am J Biol Anthropol. 2023;180: 589–617. doi:10.1002/ajpa.24718

9. Kaplan H, Hill K, Lancaster J, Hurtado AM. A theory of human life history evolution: Diet, intelligence, and longevity. Evol Anthropol Issues News Rev. 2000;9: 156–185. doi:10.1002/1520-6505(2000)9:4%3C156::AID-EVAN5%3E3.0.CO;2-7

10. Leonard WR, Snodgrass JJ, Robertson ML. Effects of Brain Evolution on Human Nutrition and Metabolism. Annu Rev Nutr. 2007;27: 311–327. doi:10.1146/annurev.nutr.27.061406.093659

11. Pontzer H. Ecological Energetics in Early Homo. Curr Anthropol. 2012;53: S346–S358. doi:10.1086/667402

12. Roebroeks W. Guts and Brains: An Integrative Approach to the Hominin Record. Leiden University Press; 2007. doi:10.24415/9789087280147

13. Foley R. Another unique species: patterns in human evolutionary ecology. Harlow: Longman Scientific & Technical; 1987.

14. Braun DR. Pliocene Hominin Behavior: New Insights. Curr Anthropol. 2019;60: 10–12. doi:10.1086/701477

15. Archer W, Braun DR, Harris JWK, McCoy JT, Richmond BG. Early Pleistocene aquatic resource use in the Turkana Basin. J Hum Evol. 2014;77: 74–87. doi:10.1016/j.jhevol.2014.02.012

16. Ferraro JV, Plummer TW, Pobiner BL, Oliver JS, Bishop LC, Braun DR, et al. Earliest Archaeological Evidence of Persistent Hominin Carnivory. PLoS ONE. 2013;8: e62174. doi:10.1371/journal.pone.0062174

17. Lee RB. The !Kung San: Men, Women and Work in a Foraging Society. CUP Archive; 1979.

18. Hawkes K, O’Connell JF, Blurton Jones NG. More Lessons from the Hadza about Men’s Work. Hum Nat. 2014;25: 596–619. doi:10.1007/s12110-014-9212-5

19. Reinhard KJ, Ambler JR, Szuter CR. Hunter-gatherer use of small animal food resources: coprolite evidence. Int J Osteoarchaeol. 2007;17: 416–428. doi:10.1002/oa.883

20. Andrade A, Fernández PM. Rodent consumption by hunter-gatherers in north Patagonian Andean forests (Argentina): Insights from the small vertebrate taphonomic analysis of two late Holocene archaeological sites. J Archaeol Sci Rep. 2017;11: 390–399. doi:10.1016/j.jasrep.2016.12.004

21. Hosaka K, Nakamura M, Takahata Y. Longitudinal changes in the targets of chimpanzee (Pan troglodytes) hunts at Mahale Mountains National Park: how and

why did they begin to intensively hunt red colobus (Piliocolobus rufomitratus) in the 1980s? Primates. 2020;61: 391–401. doi:10.1007/s10329-020-00803-8

22. Holmern T, Nyahongo J, Røskaft E. Livestock loss caused by predators outside the Serengeti National Park, Tanzania. Biol Conserv. 2007;135: 518–526. doi:10.1016/j.biocon.2006.10.049

23. Mosissa T, Ameha A, Mosissa T. Assessment of the abundance of Anubis baboons (Papio anubis) and conflict with humans in Trigni Forest of Gida Ayana District, Western Ethiopia. J Ecol Nat Environ. 2017;9: 1–14. doi:10.5897/JENE2017.0622

24. Sogbohossou EA, Iongh HH de, Sinsin B, Snoo GR de, Funston PJ. Human–carnivore conflict around Pendjari Biosphere Reserve, northern Benin. Oryx. 2011;45: 569–578. doi:10.1017/S0030605310001109

25. Alfaro JWL, Silva JDESE, Rylands AB. How different are robust and gracile capuchin monkeys? An argument for the use of sapajus and cebus. Am J Primatol. 2012;74: 273–286. doi:10.1002/ajp.22007

26. Martins-Junior AMG, Carneiro J, Sampaio I, Ferrari SF, Schneider H. Phylogenetic relationships among Capuchin (Cebidae, Platyrrhini) lineages: An old event of sympatry explains the current distribution of *Cebus* and *Sapajus*. Genet Mol Biol. 2018;41: 699–712. doi:https://doi.org/10.1590/1678-4685-GMB-2017-0012

27. Bergstrom ML, Hogan JD, Melin AD, Fedigan LM. The nutritional importance of invertebrates to female Cebus capucinus imitator in a highly seasonal tropical dry forest. Am J Phys Anthropol. 2019;170: 207–216. doi:10.1002/ajpa.23913

28. Fedigan LM. Vertebrate Predation in Cebus capucinus: Meat Eating in a Neotropical Monkey. Folia Primatol (Basel). 1990;54: 196–205. doi:10.1159/000156444

29. Fragaszy DM, Visalberghi E, Fedigan LM. The Complete Capuchin: The Biology of the Genus Cebus. Cambridge University Press; 2004.

30. Parker ST, Gibson KR. Object manipulation, tool use and sensorimotor intelligence as feeding adaptations in cebus monkeys and great apes. J Hum Evol. 1977;6: 623–641. doi:10.1016/S0047-2484(77)80135-8

31. Rose LM. Vertebrate Predation and Food-Sharing in Cebus and Pan. Int J Primatol. 1997;18: 727–765. doi:10.1023/A:1026343812980

32. Souza-Alves JP, Arruda de Almeida H, Rufino MG. Animal protein intake by blonde capuchin monkeys in a Brazilian semi-arid environment. J Nat Hist. 2026;60: 97–103. doi:10.1080/00222933.2025.2598134

33. Kinzey WG. New World Primates: Ecology, Evolution, and Behavior. Aldine de Gruyter; 1997.

34. Izar P, Verderane MP, Peternelli-dos-Santos L, Mendonça-Furtado O, Presotto A, Tokuda M, et al. Flexible and conservative features of social systems in tufted capuchin monkeys: comparing the socioecology of Sapajus libidinosus and Sapajus nigritus. Am J Primatol. 2012;74: 315–331. doi:10.1002/ajp.20968

35. Rímoli J, Strier K, Ferrari S. Seasonal and longitudinal variation in the behavior of free-ranging black tufted capuchins Cebus nigritus (Goldfuss, 1809) in a fragment of Atlantic Forest in Southeastern Brazil. 2008;9: 130–146.

36. Rose LM. Meat and the Early Human Diet: Insights from Neotropical Primate Studies. In: Stanford CB, Bunn HT, editors. Meat-Eating & Human Evolution. Oxford University PressNew York, NY; 2001. pp. 141–159. doi:10.1093/oso/9780195131390.003.0008

37. Canale GR, Kierulff MCM, Chivers DJ. A Critically Endangered Capuchin Monkey (Sapajus xanthosternos) Living in a Highly Fragmented Hotspot. In: Marsh LK, Chapman CA, editors. Primates in Fragments: Complexity and Resilience. New York, NY: Springer; 2013. pp. 299–311. doi:10.1007/978-1-4614-8839-2_20

38. Falótico T. Vertebrate Predation and Tool-Aided Capture of Prey by Savannah Wild Capuchin Monkeys (Sapajus libidinosus). Int J Primatol. 2022;44: 9–20. doi:10.1007/s10764-022-00320-z

39. Falótico T, Verderane MP, Mendonça-Furtado O, Spagnoletti N, Ottoni EB, Visalberghi E, et al. Food or threat? Wild capuchin monkeys (Sapajus libidinosus) as both predators and prey of snakes. Primates J Primatol. 2018;59: 99–106. doi:10.1007/s10329-017-0631-x

40. Ferreira R, Resende BD, Mannu M, Ottoni EB, Izar P. Bird predation and prey-transfer in brown capuchin monkeys (*Cebus apella*). Neotropical Primates. 2002;10: 84–89. doi:10.62015/np.2002.v10.510

41. Filho RF, Veiga S, Bezerra B. Bearded capuchin (Sapajus libidinosus) predation on a rock cavy (Kerodon rupestris) followed by prey sharing. Primates. 2021;62: 463–466. doi:10.1007/s10329-021-00894-x

42. Galetti M. Predation on the squirrel, Sciurus aestuans by capuchin monkeys, Cebus apella. Predation Squirrel Sciurus Aestuans Capuchin Monkeys Cebus Apella. 1990;54: 152–154.

43. Gentil E, Bezerra B, Medeiros LA de, Barnett AA. Predation by white-fronted capuchin monkeys, Cebus albifrons on eggs of three species of freshwater turtles in Brazilian Amazonia: solitary nests are also depredated. J Nat Hist. 2021;55: 1983–1997. doi:10.1080/00222933.2021.1977863

44. Milano MZ, Monteiro-Filho ELA. Predation on small mammals by capuchin monkeys, Cebus cay. Neotropical Primates. 2009;16: 78–80. doi:10.1896/044.016.0210

45. Newcomer MW, De Farcy DD. White-Faced Capuchin (Cebus capucinus) Predation on a Nestling Coati (Nasua narica). J Mammal. 1985;66: 185–186. doi:10.2307/1380983

46. Perry S, Rose L. Begging and transfer of coati meat by white-faced capuchin monkeys,Cebus capucinus. Primates. 1994;35: 409–415. doi:10.1007/BF02381950

47. Resende BD, Greco VLG, Ottoni EB, Izar P. Some observations on the predation of small mammals by tufted capuchin monkeys (*Cebus apella*). Neotropical Primates. 2003;11: 103–104. doi:10.62015/np.2003.v11.543

48. Rose LM, Perry S, Panger MA, Jack K, Manson JH, Gros-Louis J, et al. Interspecific Interactions Between Cebus capucinus and Other Species: Data from Three Costa Rican Sites. Int J Primatol. 2003;24: 759–796. doi:10.1023/A:1024624721363

49. Terborgh J. Five New World Primates: A Study in Comparative Ecology. Princeton University Press; 1983. doi:10.2307/j.ctt7ztr6n

50. Rodrigues KC, Delfim FR, Castro CSS de, França FGR, Filho EL, Mesquita DO, et al. Strobilurus torquatus Wiegmann, 1834 (Squamata: Tropiduridae): new records from the Brazilian state of Paraíba and a geographic distribution map. Check List. 2013;9: 614–617. doi:10.15560/9.3.614

51. Sampaio DT, Ferrari SF. Predation of an Infant Titi Monkey (Callicebus moloch) by a Tufted Capuchin (Cebus apella). Folia Primatol (Basel). 2005;76: 113–115. doi:10.1159/000083617

52. Carretero-Pinzón X, Defler TR, Ferrari SF. Observation of Black-Capped Capuchins (Cebus apella) Feeding on an Owl Monkey (Aotus brumbacki) in the Colombian Llanos. Neotropical Primates. 2008;15: 62–63. doi:10.1896/044.015.0210

53. Albuquerque NM, Silvestre SM, Cardoso TS, Ruiz-Esparza JM, Rocha PA, Beltrão-Mendes R, et al. Capture of a common marmoset (Callithrix jacchus) by a capuchin monkey (Sapajus sp.) in the Ibura National Forest, Sergipe (Brazil). Neotropical Primates. 2014;21: 219–221. doi:10.1896/044.021.0216

54. Colan-Rodriguez RW, Herrera-Huayhua CM, Mendoza-Soto JA, Quispe-Saenz C, Reyes-Lizarraga GM, Echevarria-Macassi LA, et al. Predation on two brown-eared woolly opossums (Caluromys lanatus) by a group of large-headed capuchin monkeys (Sapajus macrocephalus) and notes on their hierarchical feeding behaviour. Neotropical Primates. 2023;29. doi:10.62015/np.2023.v29.819

55. Ezcurra MD, Scheyer TM, Butler RJ. The Origin and Early Evolution of Sauria: Reassessing the Permian Saurian Fossil Record and the Timing of the Crocodile-Lizard Divergence. PLOS ONE. 2014;9: e89165. doi:10.1371/journal.pone.0089165

56. Wright KA, Biondi L, Visalberghi E, Ma Z, Izar P, Fragaszy D. Positional behavior and substrate use in wild adult bearded capuchin monkeys (Sapajus libidinosus). Am J Primatol. 2019;81: e23067. doi:10.1002/ajp.23067

57. Truppa V, Carducci P, Sabbatini G. Object grasping and manipulation in capuchin monkeys (genera Cebus and Sapajus). Biol J Linn Soc. 2019;127: 563–582. doi:10.1093/biolinnean/bly131

58. Rose LM. Sex differences in diet and foraging behavior in white-faced capuchins (Cebus capucinus). Int J Primatol. 1994;15: 95–114. doi:10.1007/BF02735236

59. Watts DP, Mitani JC. Hunting Behavior of Chimpanzees at Ngogo, Kibale National Park, Uganda. Int J Primatol. 2002;23: 1–28. doi:10.1023/A:1013270606320

60. Gilby IC, Emery Thompson M, Ruane JD, Wrangham R. No evidence of short-term exchange of meat for sex among chimpanzees. J Hum Evol. 2010;59: 44–53. doi:10.1016/j.jhevol.2010.02.006

61. Gilby IC, Machanda ZP, O'Malley RC, Murray CM, Lonsdorf EV, Walker K, et al. Predation by female chimpanzees: Toward an understanding of sex differences in meat acquisition in the last common ancestor of *Pan* and *Homo*. J Hum Evol. 2017;110: 82–94. doi:10.1016/j.jhevol.2017.06.015

62. Key C, Ross C. Sex differences in energy expenditure in non–human primates. Proc R Soc Lond B Biol Sci. 1999;266: 2479–2485. doi:10.1098/rspb.1999.0949

63. Fragaszy DM, Izar P, Liu Q, Eshchar Y, Young LA, Visalberghi E. Body mass in wild bearded capuchins, (Sapajus libidinosus): Ontogeny and sexual dimorphism. Am J Primatol. 2016;78: 473–484. doi:10.1002/ajp.22509

64. McCabe GM, Fedigan LM. Effects of Reproductive Status on Energy Intake, Ingestion Rates, and Dietary Composition of Female Cebus capucinus at Santa Rosa, Costa Rica. Int J Primatol. 2007;28: 837–851. doi:10.1007/s10764-007-9159-z

65. Westergaard GC, Suomi SJ. Transfer of tools and food between groups of tufted capuchins (Cebus apella). Am J Primatol. 1997;43: 33–41. doi:10.1002/(SICI)1098-2345(1997)43:1%3C33::AID-AJP2%3E3.0.CO;2-Z

66. Boesch C, Boesch H. Hunting behavior of wild chimpanzees in the Taï National Park. Am J Phys Anthropol. 1989;78: 547–573. doi:10.1002/ajpa.1330780410

67. Boesch C, Kohou G, Néné H, Vigilant L. Male competition and paternity in wild chimpanzees of the Taï forest. Am J Phys Anthropol. 2006;130: 103–115. doi:10.1002/ajpa.20341

68. Falótico T, Ottoni EB. Sexual bias in probe tool manufacture and use by wild bearded capuchin monkeys. Behav Processes. 2014;108: 117–122. doi:10.1016/j.beproc.2014.09.036

69. Edwards W, Lonsdorf EV, Pontzer H. Total energy expenditure in captive capuchins (Sapajus apella). Am J Primatol. 2017;79: e22638. doi:10.1002/ajp.22638

70. Spagnoletti N, Visalberghi E, Verderane MP, Ottoni E, Izar P, Fragaszy D. Stone tool use in wild bearded capuchin monkeys, *Cebus libidinosus*. Is it a strategy to overcome food scarcity? Anim Behav. 2012;83: 1285–1294. doi:10.1016/j.anbehav.2012.03.002

71. Spagnoletti N, Visalberghi E, Ottoni E, Izar P, Fragaszy D. Stone tool use by adult wild bearded capuchin monkeys (*Cebus libidinosus*). Frequency, efficiency and tool selectivity. J Hum Evol. 2011;61: 97–107. doi:10.1016/j.jhevol.2011.02.010

72. Visalberghi E, Fragaszy D. The Etho- *Cebus* Project: Stone-tool use by wild capuchin monkeys. 1st ed. In: Sanz CM, Call J, Boesch C, editors. Tool Use in Animals. 1st ed. Cambridge University Press; 2013. pp. 203–222. doi:10.1017/CBO9780511894800.013

73. IUCN. Sapajus libidinosus: Martins, A.B., Fialho, M.S., Jerusalinsky, L., Valença-Montenegro, M.M., Bezerra, B.M., Laroque, P.O., de Melo, F.R. & Lynch Alfaro, J.W.: The IUCN Red List of Threatened Species 2021: e.T136346A192593226. 2015. doi:10.2305/IUCN.UK.2021-1.RLTS.T136346A192593226.en

74. Martin PR, Bateson P. Measuring behaviour: an introductory guide. 3rd ed. Cambridge: Cambridge University Press; 2007.

75. Altmann J. Observational Study of Behavior: Sampling Methods. 1974 [cited 16 Sept 2025]. doi:10.1163/156853974X00534

76. Akaike H. Information Theory and an Extension of the Maximum Likelihood Principle. In: Parzen E, Tanabe K, Kitagawa G, editors. Selected Papers of Hirotugu Akaike. New York, NY: Springer New York; 1998. pp. 199–213. doi:10.1007/978-1-4612-1694-0_15

77. Bolker BM, Brooks ME, Clark CJ, Geange SW, Poulsen JR, Stevens MHH, et al. Generalized linear mixed models: a practical guide for ecology and evolution. Trends Ecol Evol. 2009;24: 127–135. doi:10.1016/j.tree.2008.10.008

78. Buckley YM. Generalized linear models. 1st ed. In: Fox GA, Negrete-Yankelevich S, Sosa VJ, editors. Ecological Statistics. 1st ed. Oxford University PressOxford; 2015. pp. 131–148. doi:10.1093/acprof:oso/9780199672547.003.0007

79. Blasco-Moreno A, Pérez-Casany M, Puig P, Morante M, Castells E. What does a zero mean? Understanding false, random and structural zeros in ecology. Methods Ecol Evol. 2019;10: 949–959. doi:10.1111/2041-210X.13185

80. Martin TG, Wintle BA, Rhodes JR, Kuhnert PM, Field SA, Low-Choy SJ, et al. Zero tolerance ecology: improving ecological inference by modelling the source of zero observations. Ecol Lett. 2005;8: 1235–1246. doi:10.1111/j.1461-0248.2005.00826.x

81. Zuur AF, Ieno EN. A protocol for conducting and presenting results of regression-type analyses. Methods Ecol Evol. 2016;7: 636–645. doi:10.1111/2041-210X.12577

82. Harrison XA, Donaldson L, Correa-Cano ME, Evans J, Fisher DN, Goodwin CED, et al. A brief introduction to mixed effects modelling and multi-model inference in ecology. PeerJ. 2018;6: e4794. doi:10.7717/peerj.4794

83. Fox GA, Negrete-Yankelevich S, Sosa VJ, editors. Ecological Statistics: Contemporary theory and application. 1st ed. Oxford University PressOxford; 2015. doi:10.1093/acprof:oso/9780199672547.001.0001

84. Lindén A, Mäntyniemi S. Using the negative binomial distribution to model overdispersion in ecological count data. Ecology. 2011;92: 1414–1421. doi:10.1890/10-1831.1

85. Brooks ME, Kristensen K, Benthem KJ van, Magnusson A, Berg CW, Nielsen A, et al. Modeling zero-inflated count data with glmmTMB. bioRxiv; 2017. p. 132753. doi:10.1101/132753

86. Brooks M, Kristensen K, Benthem K van, Magnusson A, Berg C, Nielsen A, et al. glmmTMB Balances Speed and Flexibility Among Packages for Zero-inflated Generalized Linear Mixed Modeling. R J. 2017. Available: https://digitalcommons.unl.edu/r-journal/675

87. R Core Team. R: A language and environment for statistical computing [computer program]. Vienna: R Foundation for Statistical Computing; 2022.

88. Richards SA. Dealing with overdispersed count data in applied ecology. J Appl Ecol. 2008;45: 218–227. doi:10.1111/j.1365-2664.2007.01377.x

89. McGrew WC. Chimpanzee Material Culture: Implications for Human Evolution. Cambridge University Press; 1992.

90. Saj T, Sicotte P, Paterson JD. Influence of Human Food Consumption on the Time Budget of Vervets. Int J Primatol. 1999;20: 977–994. doi:10.1023/A:1020886820759

91. Sengupta A, McConkey KR, Radhakrishna S. Primates, Provisioning and Plants: Impacts of Human Cultural Behaviours on Primate Ecological Functions. PLOS ONE. 2015;10: e0140961. doi:10.1371/journal.pone.0140961

92. Asquith PJ. Provisioning and the study of free-ranging primates: History, effects, and prospects. Am J Phys Anthropol. 1989;32: 129–158. doi:10.1002/ajpa.1330320507

93. Bobe R, Behrensmeyer AK, Chapman RE. Faunal change, environmental variability and late Pliocene hominin evolution. J Hum Evol. 2002;42: 475–497. doi:10.1006/jhev.2001.0535

94. Hopper LM, Ross SR, Goodall J. Chimpanzees in Context: A Comparative Perspective on Chimpanzee Behavior, Cognition, Conservation, and Welfare. University of Chicago Press; 2020. doi:10.7208/chicago/9780226728032.001.0001

95. Gilby IC, Wrangham RW. Risk-prone hunting by chimpanzees (Pan troglodytes schweinfurthii) increases during periods of high diet quality. Behav Ecol Sociobiol. 2007;61: 1771–1779. doi:10.1007/s00265-007-0410-6

96. Izar P, Peternelli-dos-Santos L, Rothman JM, Raubenheimer D, Presotto A, Gort G, et al. Stone tools improve diet quality in wild monkeys. Curr Biol. 2022;32: 4088-4092.e3. doi:10.1016/j.cub.2022.07.056

97. Goffe AS, Fischer J. Meat sharing between male and female Guinea baboons (*Papio papio*). Primate Biol. 2016;3: 1–8. doi:10.5194/pb-3-1-2016

98. Hamilton WJ III, Busse CD. Primate Carnivory and Its Significance to Human Diets. BioScience. 1978;28: 761–766. doi:10.2307/1307249

99. Harding R. Meat-eating and hunting in baboons. Socioecology and psychology of primates. The Hague: Mouton; 1975. pp. 245–257.

100. Hausfater G. Predatory Behavior of Yellow Baboons. Behaviour. 1976;56: 44–68. Available: https://www.jstor.org/stable/4533713

101. Rhine RJ, Norton GW, Wynn GM, Wynn RD, Rhine HB. Insect and meat eating among infant and adult baboons (Papio cynocephalus) of Mikumi National Park, Tanzania. Am J Phys Anthropol. 1986;70: 105–118. doi:10.1002/ajpa.1330700115

102. Strum SC. 8. Processes and Products of Change: Baboon Predatory Behavior at Gilgil, Kenya. In: Harding RSO, Teleki G, editors. Omnivorous Primates Gathering and Hunting in Human Evolution. Columbia University Press; 2019. pp.

255–302. Available: https://www.degruyterbrill.com/document/doi/10.7312/hard92188-009/html

103. Anderson A, Chilczuk S, Nelson K, Ruther R, Wall-Scheffler C. The Myth of Man the Hunter: Women's contribution to the hunt across ethnographic contexts. PLOS ONE. 2023;18: e0287101. doi:10.1371/journal.pone.0287101

104. Venkataraman VV, Hoffman J, Farquharson K, Davis HE, Hagen EH, Hames RB, et al. Female foragers sometimes hunt, yet gendered divisions of labor are real: a comment on Anderson et al. (2023) The Myth of Man the Hunter. Evol Hum Behav. 2024;45: 106586. doi:10.1016/j.evolhumbehav.2024.04.014

105. Marlowe FW. Hunter-gatherers and human evolution. Evol Anthropol Issues News Rev. 2005;14: 54–67. doi:10.1002/evan.20046

106. Marlowe FW. Hunting and Gathering: The Human Sexual Division of Foraging Labor. Cross-Cult Res. 2007;41: 170–195. doi:10.1177/1069397106297529

107. Murdock GP, Provost C. Factors in the Division of Labor by Sex: A Cross-Cultural Analysis. Ethnology. 1973;12: 203–225. doi:10.2307/3773347

108. Goodman MJ, Griffin PB, Estioko-Griffin AA, Grove JS. The compatibility of hunting and mothering among the agta hunter-gatherers of the Philippines. Sex Roles. 1985;12: 1199–1209. doi:10.1007/BF00287829

109. Hewlett BS. Intimate fathers : the nature and context of Aka pygmy paternal infant care. Ann Arbor : University of Michigan Press; 1991. Available: http://archive.org/details/intimatefathersn0000hewl

110. Noss AJ, Hewlett BS. The Contexts of Female Hunting in Central Africa. Am Anthropol. 2001;103: 1024–1040. doi:10.1525/aa.2001.103.4.1024

111. Tonkinson R. The Mardudjara Aborigines: Living the Dream in Australia's Desert. Holt, Rinehart and Winston; 1978.

112. Gilby IC, Machanda ZP, O'Malley RC, Murray CM, Lonsdorf EV, Walker K, et al. Predation by female chimpanzees: Toward an understanding of sex differences in meat acquisition in the last common ancestor of *Pan* and *Homo*. J Hum Evol. 2017;110: 82–94. doi:10.1016/j.jhevol.2017.06.015

113. McGrew WC, Tutin CEG, Baldwin PJ. Chimpanzees, Tools, and Termites: Cross-Cultural Comparisons of Senegal, Tanzania, and Rio Muni. Man. 1979;14: 185–214. doi:10.2307/2801563

114. Tennie C, O'Malley RC, Gilby IC. Why do chimpanzees hunt? Considering the benefits and costs of acquiring and consuming vertebrate versus invertebrate prey. J Hum Evol. 2014;71: 38–45. doi:10.1016/j.jhevol.2014.02.015

115. Bird R. Cooperation and conflict: The behavioral ecology of the sexual division of labor. Evol Anthropol Issues News Rev. 1999;8: 65–75. doi:10.1002/(SICI)1520-6505(1999)8:2%3C65::AID-EVAN5%3E3.0.CO;2-3

116. Aquino ID, González-Santoyo I, Link A, Muñoz-Delgado J. An exploratory study of cooperation: food-sharing behaviour in wild varied white-fronted capuchin monkeys (Cebus versicolor) in Central Colombia. 2022 [cited 14 Sept 2025]. doi:10.1163/1568539X-bja10180

117. Samuni L, Preis A, Deschner T, Crockford C, Wittig RM. Reward of labor coordination and hunting success in wild chimpanzees. Commun Biol. 2018;1: 138. doi:10.1038/s42003-018-0142-3

118. Kyriacou K, Blackhurst DM, Parkington JE, Marais AD. Marine and terrestrial foods as a source of brain-selective nutrients for early modern humans in the southwestern Cape, South Africa. J Hum Evol. 2016;97: 86–96. doi:10.1016/j.jhevol.2016.04.009

119. Woodard HQ, White DR. The composition of body tissues. Br J Radiol. 1986;59: 1209–1218. doi:10.1259/0007-1285-59-708-1209

120. Gilby IC, Wawrzyniak D. Meat Eating by Wild Chimpanzees (Pan troglodytes schweinfurthii): Effects of Prey Age on Carcass Consumption Sequence. Int J Primatol. 2018;39: 127–140. doi:10.1007/s10764-018-0019-9

121. Goodall J. The chimpanzees of Gombe: patterns of behavior. Cambridge, Massachusetts London, England: The Belknap Press of Harvard University Press; 1986.

122. Pruetz JD, Bertolani P, Ontl KB, Lindshield S, Shelley M, Wessling EG. New evidence on the tool-assisted hunting exhibited by chimpanzees (Pan troglodytes verus) in a savannah habitat at Fongoli, Sénégal. R Soc Open Sci. 2015;2: 140507. doi:10.1098/rsos.140507

Supporting information

S1 File. Cleaning and pre-processing.

S2 File. Study Data.

S1 Fig. Full dataset, showing all predation events per individual and month. The size of the circles indicates the number of events (from 0 to 6) in each month and the circle color indicates the age class to which the individual belongs each month.

S2 Fig. Predations events across the study period 56 months, starting in May 2006. Notice the 11 months gap from May 2008 to March 2009. Group information is also provided.

S3 Fig. Full dataset after cleaning. Includes 10 fewer individuals, showing all predation events per individual and month. The sex of each individual is indicated by color (red = male, blue = female)~~-~~.

S1 Table. Variance Inflation Factors (VIF) for the Consumption Model. VIF values < 5 indicate low multicollinearity; values near 1 indicate nearly no correlation between predictors.

S2~~1~~ Table. Sub-model of mammalian consumption. Number of observations: 1092, random effects: ID (36); Month (45). Final model metrics: AIC = 502.6; BIC = 572.5; logLik = -237.3; deviance = 474.6; df residuals = 1078.

S3~~2~~ Table. Sub-model of saurian consumption. Number of observations: 1092, random effects: ID (36); Month (45). Final model metrics: AIC = 920.3; BIC = 990.2; logLik = -446.1; deviance = 892.3; df residuals = 1078.

Formatted: Font: Bold

S1 Video. Vertebrate consumption. (a) A young capuchin eating a vertebrate; (b) Capuchin feeding on a lizard. This video was filmed by Alessandro Albani for the documentary "The bearded capuchin monkeys of Fazenda Boa Vista" (https://www.youtube.com/watch?v=7ojU43JgpYo), not recorded during the study, but it illustrates what we report here.

~~**S3**~~**3**~~**2 Video. Food transfer.** (a) A Subadult male has captured a great kiskadee (*Pitangus sulphuratus*) and moves in the canopy holding its carcass. (b) Other group members show interest towards it and approach the possessor. (c) Food transfer may occur with the possessor tolerating the recipient to take a piece of the prey. (d) An infant, who has obtained mostly bones with very little meat, is chewing the same kiskadee initially captured by the subadult male. This video was filmed by Alessandro Albani for the documentary "The bearded capuchin monkeys of Fazenda Boa Vista" (https://www.youtube.com/watch?v=7ojU43JgpYo), not recorded during the study, but it illustrates what we report here.~~

S2~~3~~ Video. A hunting event of Type II (collective). Four young capuchins hidden in a bush attack an Iguana (*Iguana iguana*) passing nearby. One grasps and detaches the far end of the iguana tail. Meat transfer should have occurred while the capuchins were into the bush. However, it is important to note that sooner or later we observed all four of them eating parts of the iguana. This video was filmed by Alessandro Albani for the documentary "The bearded capuchin monkeys of Fazenda Boa Vista" (https://www.youtube.com/watch?v=7ojU43JgpYo), not recorded during the study, but it illustrates what we report here.

S3 Video. Food transfer. (a) A Subadult male has captured a great kiskadee (*Pitangus sulphuratus*) and moves in the canopy holding its carcass. (b) Other group members show

Formatted: Font: Bold

Formatted: Font: Italic

interest towards it and approach the possessor. (c) Food transfer may occur with the possessor tolerating the recipient to take a piece of the prey. (d) An infant, who has obtained mostly bones with very little meat, is chewing the same kiskadee initially captured by the subadult male. This video was filmed by Alessandro Albani for the documentary "The bearded capuchin monkeys of Fazenda Boa Vista" (https://www.youtube.com/watch?v=7ojU43JgpYo), not recorded during the study, but it illustrates what we report here.

S1 Checklist. Inclusivity in global research information. Additional information regarding the ethical, cultural, and scientific considerations specific to inclusivity in global research in this study.

Formatted: Font: Bold

Formatted: Font: (Default) Times New Roman

Reviewer #1

1. **Hypothesis #6, Necessity:**

I believe that this hypothesis is more linked to the absence or reduction of the fruit and invertebrate availability than to environmental factors. We need to think (ecologically) that such variables can indirectly affect the fruit and invertebrate availabilities. So, the better fashion - I think - to test or think is associating with the food resources. The variable influencing diet and behaviour is food items and not rainfall, temperature, and humidity. These can or not influence the appearance of fruit and invertebrates in a given site. Furthermore, to test this hypothesis, perhaps it is better to relate vertebrate consumption with fruit consumption. Thus, the authors could demonstrate the necessity to exploit other food resources.

Response: Thank you for your feedback. We have edited the framing of this hypothesis so that it does not address necessity, but rather simply tests for environmental effects over predation rates in a way suggested by Reviewer #2. We hope the new framing of our hypothesis can better address this concern.

2. **Environmental variables:**

The manuscript works with three variables – rainfall, temperature, and humidity – to potentially explain the variation in vertebrate consumption by capuchins. However, two of these variables (rainfall and humidity) will have an effect on fruit and insect availability. So, I do not believe that both can contribute to the understanding of the findings. Thus, to test

Response:

Correlations among predictor variables were assessed using Spearman's rank correlation coefficient and variance inflation factors. Spearman's ρ indicated only low to moderate pairwise associations, and all VIF values were below 1.25, suggesting negligible multicollinearity. The strongest association was a moderate correlation between insect availability and rainfall (ρ=0.45). However, low VIF values indicated that the model was still able to distinguish between the seasonal increase in insect availability and the direct effect of rainfall on capuchin predation activity. In addition, model comparison based on AIC values showed that models including both environmental variables (rainfall, temperature, and humidity) and food availability variables (fruit and insect availability) consistently outperformed models including environmental variables alone. Because our a priori model selection procedure was based on comparing biologically motivated candidate models and retaining the model with the strongest AIC support, both the environmental variables and the fruit and insect availability variables were therefore kept in the final model. We have added an explicit clarification of this procedure in the methods (subsection Data analysis: statistical modelling) and a supplementary table with VIF values (S1 Table).

3. **Energy shortfall hypothesis:**

I believe that a correlation between vertebrate consumption vs. fruit consumption could help us to understand this hypothesis. The fruit availability is a great measure. But, it does not

demonstrate whether the fruit consumption is (positively or negatively) linked to vertebrate intake. However, it needs to know if fruit consumption is associated with vertebrate consumption.

Response: Thank you for your feedback. While we appreciate that fruit consumption would have been a very interesting covariate to include in our model, we are not able to collect the necessary data retrospectively. However, we included and expanded on this limitation in our Discussion.

4. **Humidity:**

I would like to see a discussion related to the influence of humidity on the probability of an excess zero in the predation events. If such a variable was measured and incorporated in the study (i.e., data analysis) is necessary to discuss it.

Response: Thank you for your feedback. We have now included a discussion of the influence of humidity on this variable in our Discussion section.

5. **Figures:**

I do not know the reason, but the figures are not included in the manuscript to review. So, it was not possible to evaluate some scenarios inserted in the text.

Response: We have now included all relevant figures.

Reviewer #2

1. **Introduction:**

The introduction to this manuscript would benefit from major revisions to tighten it up. While relevant literature is cited, the current structure reads more like an information dump instead of a focused funnel toward the hypotheses. Redundant and overly dense sentences obscure the core theoretically background and the importance of the hypotheses the authors propose to text. Additionally, several references appear to be incorrectly formatted starting in the Into and persisting throughout the manuscript. For instance, line 169 reads "When reviewed by (1),…" where an author name should appear. The paper as whole would benefit from a careful review for these in-text citations.

Response: Thank you for your feedback. The introduction was majorly revised and edited according to these comments, better structured as the "focused funnel toward the hypotheses" the reviewer suggested. We have also reviewed all in-text citations and edited incorrect formatting.

2. **Hypotheses:**

The authors present a thoughtful hypothesis framework to address the drivers of vertebrate predation in these groups of capuchins. Testing predation rates between provision and non provisioned groups is well conceived. The protein in availability hypothesis is reasonable since predation might be driven by the need for macro and micronutrient needs.

Hypothesis 1 and hypothesis 2.1 appeared to be contradictory as written. Hypothesis one predicts that males will have higher vertebrate predation rates because of their increased body mass. Whereas hypothesis 2.1 predicts that females will have higher vertebrate predation rates than adult males because of reproduction and lactation needs. Would a better test of hypothesis 2.1 be that pregnant or lactating females would have increased vertebrate predation rates than non-pregnant non lactating females? Or, and it's not clear if this possible from that dataset, since you know individuals in the dataset could you compare individuals of similar body mass?

Hypotheses 5 and 6 should be presented as a null and alternative. As written these two hypotheses are testing if predation rates are influenced by environmental factors. The authors are then interpreting whether it's opportunistic or necessity. In my opinion this would better be presented as a null with an alternative that environment impacts predation rates and save the discussion of opportunity and necessity in the context of different prey types for the discussion.

The last hypothesis concerns food transfer and hunting and wild capuchins. This hypothesis is presented differently than the previous hypotheses, with the hypothesis buried within the text of the paragraph. I wonder if the authors could weave in the discussion of hunting and food transfer among primates in general and capuchin specifically somewhere in the main part of the introduction prior to introducing their hypotheses.

Response:

Thank you for your feedback. Regarding hypotheses 1 and 2.1 (now presented as 1.1 and 1.2 in the revised manuscript), testing whether pregnant or lactating females have increased vertebrate predation rates than non-pregnant or non-lactating females is not possible from the dataset. However, we have now presented the two hypotheses as alternatives concerning sex biases on predation rates: either males will have higher predation rates, or females will (or neither of the two). We hope this revised format can clarify the need for alternative hypotheses regarding sex biases in predation rates.

Regarding hypotheses 5 and 6 (now presented as 5.1 and 5.2), we have re-framed these as null and alternative as suggested by the reviewer.

Regarding the hypothesis on food transfer and hunting: we have included this hypothesis with the others and added the discussion on hunting and food transfer in the main part of the introduction prior to the hypotheses, as suggested by the reviewer.

3. **Methods:**

The statistical modeling approach is used here appears appropriate and well justified. Maybe I missed this, but why is rainfall in the conditional model but not in the zero inflation model? Was that an a priori decision or was it from AIC comparisons? I assume, perhaps incorrectly, that humidity and rainfall or rainfall and fruit/insect availability covary somewhat. Did the authors test for correlations among predictors? Could this be addressed with a sentence or two in the methods?

Response:

Rainfall is in the conditional model but not in the zero inflation model because this model structure improved BIC and AIC substantially (and it also helped a lot with the overall modelling architecture, as models tended to not be able to converge in the opposite solution).

Correlations among predictor variables were assessed using Spearman's rank correlation coefficient and variance inflation factors. Spearman's ρ indicated only low to moderate pairwise associations, and all VIF values were below 1.25, suggesting negligible multicollinearity. The strongest association was a moderate correlation between insect availability and rainfall (ρ=0.45). However, low VIF values indicated that the model was still able to distinguish between the seasonal increase in insect availability and the direct effect of rainfall on capuchin predation activity. In addition, model comparison based on AIC values showed that models including both environmental variables (rainfall, temperature, and humidity) and food availability variables (fruit and insect availability) consistently outperformed models including environmental variables alone. Because our a priori model selection procedure was based on comparing biologically motivated candidate models and retaining the model with the strongest AIC support, both the environmental variables and the fruit and insect availability variables were therefore kept in the final model. We have addressed this explicitly in the methods (subsection Data analysis: statistical modelling) and added a supplementary table with VIF values (S1 Table).

4. **Results:**

With the caveat that I can't evaluate the results completely without the figures, the results are clearly reported.

Response: We have now included all relevant figures.

5. **Discussion:**

I have no major concerns about the discussion. A minor suggestion is to integrate Table 5 into the results.

Response: Thank you for your feedback. However, we believe Table 5 is better suited for a visual summary of results in the Discussion section than for the Results. The Results already thoroughly detail the information in Table 5, so including it in this section would be repetitive. In contrast, the presence of Table 5 in the Discussion makes the dissection of the main results of this paper easier for the reader, as there are many hypotheses addressed in this article.

Academic editor

1. Please ensure that your manuscript meets PLOS ONE's style requirements, including those for file naming. The PLOS ONE style templates can be found at https://journals.plos.org/plosone/s/file?id=wjVg/PLOSOne_formatting_sample_main_body.pdf and https://journals.plos.org/plosone/s/file?id=ba62/PLOSOne_formatting_sample_title_authors_affiliations.pdf.

Response: All style templates have been checked and applied. Image files have been re-formatted as per instructions.

2. Please include a complete copy of PLOS' questionnaire on inclusivity in global research in your revised manuscript. Our policy for research in this area aims to improve transparency in the reporting of research performed outside of researchers' own country or community. The policy applies to researchers who have travelled to a different country to conduct research, research with Indigenous populations or their lands, and research on cultural artefacts. The questionnaire can also be requested at the journal's discretion for any other submissions, even if these conditions are not met. Please find more information on the policy and a link to download a blank copy of the questionnaire here: https://journals.plos.org/plosone/s/best-practices-in-research-reporting . Please upload a completed version of your questionnaire as Supporting Information when you resubmit your manuscript.

Response: The questionnaire has been completed and is uploaded as Checklist_S1.pdf. We reference this checklist in our updated Methods section, subsection "Inclusivity in global research".

3. In your Methods section, please provide additional information regarding the permits you obtained for the work. Please ensure you have included the full name of the authority that approved the field site access and, if no permits were required, a brief statement explaining why.

Response: Our "Ethical note" section has been updated to provide additional information on the permits obtained for the work and the authority that approved field access.

4. Thank you for stating in your Funding Statement:

"This work was supported by University La Sapienza of Rome (NS), EU FP6 NEST Programme ANALOGY (NS: No 029088), FAPESP (MPV: 06/51578-9, OMF: 08/52293-3 and PI: 06/51577-2 and 08/55684-3), CNPq; CAPES (NS: 017/2012), ISTC-CNR, Ethoikos srl. The funders had no role in study design, data collection and analysis, decision to publish, or preparation of the manuscript.".

Please provide an amended statement that declares *all* the funding or sources of support (whether external or internal to your organization) received during this study, as detailed online in our guide for authors at http://journals.plos.org/plosone/s/submit-now. Please also include the statement "There was no additional external funding received for this study." in your updated Funding Statement.

Please include your amended Funding Statement within your cover letter. We will change the online submission form on your behalf.

Response: We have included the amended Funding Statement in our cover letter (Cover Letter Plos One with Amended Funding Statement.docx).

5. Please upload a copy of Figure 1-6, to which you refer in your text. If the figure is no longer to be included as part of the submission please remove all reference to it within the text.

Response: We have now uploaded copies of Figures 1-6 as .tiff files and in correct formatting.

6. If the reviewer comments include a recommendation to cite specific previously published works, please review and evaluate these publications to determine whether they are relevant and should be cited. There is no requirement to cite these works unless the editor has indicated otherwise.

Response: All citation recommendations by reviewers were evaluated as relevant and were included as references in the updated manuscript.
