# Supplementary material for: Small vertebrate consumption by capuchin monkeys (Sapajus libidinosus): Insights into the human predatory pattern
Source: PLoS One. 2026 Aug 26;21(8):e0355204. doi: 10.1371/journal.pone.0355204 (PMC13514029; doi:10.1371/journal.pone.0355204)
Supplement: Supplementary file 3 [file pone.0355204.s016.pdf]

General comments:

Firstly, congratulations to the authors on this work, which clearly involved extensive fieldwork. Regarding the manuscript text, I agree with the previous Reviewer 2 that the manuscript would benefit from a thorough general review to improve clarity and, specifically, to address overly long sentences. For example, lines 170 to 175 consist of a single six-line sentence. Please break down such long sentences into shorter phrases. As a rule of thumb, try to keep a "one sentence, one idea" approach throughout the text.

Specific comments:

Query 1: Please ensure that the header row is repeated on every page for Table 1.

Query 2: Line 152: Please specify the country where “Fazenda Boa Vista” is located. The same applies to “Santa Rosa” on line 164.

Query 3: Line 194: The font size and color of the numbers are inconsistent with the rest of the text. Similarly, lines 571, 685, and 686 contain words with formatting inconsistencies (different font sizes/colors). Please standardize the typography.

Query 4: In Hypotheses 4.1 and 4.2, it is stated that vertebrate predation rates increase during periods of fruit and insect scarcity because vertebrates replace these other high-protein food items. Does this imply that fruits and insects are preferred over vertebrates? Considering that vertebrates generally offer higher protein content than insects and fruits, wouldn't the predators naturally prefer vertebrates and only rely on other items when vertebrates are harder to find? Please clarify this ecological rationale.

Query 5: Please insert the equation immediately after it is first mentioned in the text.

Query 6: Table 5: I agree with the previous Reviewer 2 that Table 5 belongs in the Results section rather than the Discussion. While I understand the authors' intent to summarize findings within the Discussion, this can be achieved via a concise textual summary. Presenting raw data in a table format is unsuited for the Discussion section, as it shifts the focus back to data description rather than critical discussion.

Query 7 : In the Discussion section, I missed a structured and explicit discussion regarding the initial hypotheses. Please clearly state which hypotheses were corroborated or refuted and directly connect these outcomes with the existing literature.
