# Supplementary material for: Small vertebrate consumption by capuchin monkeys (Sapajus libidinosus): Insights into the human predatory pattern
Source: PLoS One. 2026 Aug 26;21(8):e0355204. doi: 10.1371/journal.pone.0355204 (PMC13514029; doi:10.1371/journal.pone.0355204)
Supplement: Supplementary file 4 [file pone.0355204.s001.docx]

**Cleaning and pre-processing**

Our full dataset has 1135 data instances recording *Predation Events* observed on 46 individuals (*ID*) per *Month* (Figure S1). Observations happened during a period covering 56 months, but with some gaps, totalling 45 months of recorded data (Figure S2). The objective is to understand how *Predation Events* are affected by *Age Class* (adults, juveniles or infants), *Sex* (males or females), *Group* (ZA ‘non-provisioned’ or CH ‘provisioned’), *Temperature* (mean = 27.23°C), *Humidity* (mean = 62.84%), *Rainfall* (mean = 94.06mm), *Insect availability* (mean = 19730 kg/ha) and *Fruit availability* (mean = 287.5 kg/ha). The last five (numerical) variables were both centred and scaled to mean = 0 and standard deviation = 1, to avoid convergence issues and improve model performance and interpretability (Schielzeth, 2010). There were many individuals that have been observed preying on vertebrates only once or very few times. We removed all the individuals with less than 10 observations (APP, CC, COST, DQE, DUQ, EV, JR, MO, PTA, RN) leaving us with a total of 1092 data instances (i.e., observations) recorded on 36 individuals observed during 45 months (Figure S3).

There were 66 rows with missing values in the columns containing numerical variables. To avoid listwise deletion of entire rows at the modelling phase (Nakagawa and Freckleton, 2008), we used an unsupervised learning pipeline for data imputation with the following steps: 1) start with a rough fix by median imputation; 2) apply an unsupervised random forest (Breiman, 2001; Shi and Horvath, 2006) on all variables; 3) compute a classic multidimensional analysis (Gower, 1966) of the dissimilarity matrix *1 – proximity*, (the proximity parameter was calculated by the unsupervised random forest in the previous step) to transform the data into a set of bidimensional cartesian coordinates; 4) visualize the two dimensions using a scatter plot and inspect possible $k$ clusters; 5) calculate a $k$-means for clustering the data points (Steinhaus, 1956; Lloyd, 1982); 6) run the rfImpute algorithm (Liaw and Wiener, 2002), informed by the new clusters, for robust missing value imputation (finally, substituting the rough median imputation from step 1). This approach avoids losing data or using a simple NA imputation by some measure of average, and instead uses the information and numerical structure of all the variables and observations to data mine more realistic values.
