# Supplementary material for: Small vertebrate consumption by capuchin monkeys (Sapajus libidinosus): Insights into the human predatory pattern
Source: PLoS One. 2026 Aug 26;21(8):e0355204. doi: 10.1371/journal.pone.0355204 (PMC13514029; doi:10.1371/journal.pone.0355204)
Supplement: Supplementary file 5 [file pone.0355204.s006.docx]

| **Predictor Variable** | **VIF Value** |
| --- | --- |
| *Rainfall* | 1.164 |
| *Fruit Availability* | 1.099 |
| *Insect Availability* | 1.210 |
| *Mean Temperature* | 1.076 |
