# Supplementary material for: Small vertebrate consumption by capuchin monkeys (Sapajus libidinosus): Insights into the human predatory pattern
Source: PLoS One. 2026 Aug 26;21(8):e0355204. doi: 10.1371/journal.pone.0355204 (PMC13514029; doi:10.1371/journal.pone.0355204)
Supplement: Supplementary file 6 [file pone.0355204.s007.docx]

| **Term** | **Estimate** | **SE** | **Lower CL** | **Upper CL** | **Wald’s** | ***P*-value** |
| --- | --- | --- | --- | --- | --- | --- |
| *Conditional model (Negative Binomial)* | | | | | | |
| *(Intercept)* | -8.6936 | 0.473026 | -9.62079 | -7.76657 | -18.3788 | - |
| ***Sex*** | 0.93126 | 0.339816 | 0.265233 | 1.597289 | 2.740482 | 0.00613* |
| *Age (Juvenile)* | -0.15103 | 0.322829 | -0.78376 | 0.481704 | -0.46783 | 0.639906 |
| *Age (Infant)* | -1.72675 | 0.926709 | -3.54308 | 0.089558 | -1.86332 | 0.062417 |
| *Group (ZA)* | 0.547836 | 0.352327 | -0.14271 | 1.238386 | 1.554907 | 0.119968 |
| ***Rainfall*** | 0.990945 | 0.453726 | 0.101658 | 1.880233 | 2.184014 | 0.02896* |
| ***Fruits*** | -1.31042 | 0.386425 | -2.06779 | -0.55304 | -3.39113 | 0.00070* |
| *Insects* | -0.32574 | 0.287480 | -0.88919 | 0.237711 | -1.13309 | 0.257178 |
| *Zero Inflation model* | | | | | | |
| *(Intercept)* | -3.71552 | 2.277375 | -8.17909 | 0.748054 | -1.63149 | - |
| ***Temperature*** | -1.34733 | 0.583250 | -2.49049 | -0.20419 | -2.31005 | 0.020886* |
| *Humidity* | -0.11666 | 0.073969 | -0.26164 | 0.028318 | -1.57713 | 0.1147648 |
