# Supplementary material for: Small vertebrate consumption by capuchin monkeys (Sapajus libidinosus): Insights into the human predatory pattern
Source: PLoS One. 2026 Aug 26;21(8):e0355204. doi: 10.1371/journal.pone.0355204 (PMC13514029; doi:10.1371/journal.pone.0355204)
Supplement: Supplementary file 7 [file pone.0355204.s008.docx]

| **Term** | **Estimate** | **SE** | **Lower CL** | **Upper CL** | **Wald’s** | ***P*-value** |
| --- | --- | --- | --- | --- | --- | --- |
| *Conditional model (Negative Binomial)* | | | | | | |
| *(Intercept)* | -7.17710 | 0.305286 | -9.62079 | -7.76657 | -23.5123 | - |
| *Sex* | 0.145012 | 0.240657 | 0.265233 | 1.597289 | 1.968241 | 0.546796 |
| *Age (Juvenile)* | -0.30517 | 0.248978 | -0.78376 | 0.481704 | -1.02659 | 0.220313 |
| ***Age (Infant)*** | -1.35417 | 0.575559 | -3.54308 | 0.089558 | -3.15864 | 0.018633* |
| ***Group (ZA)*** | 0.673783 | 0.243319 | -0.14271 | 1.238386 | 3.454114 | 0.005621* |
| *Rainfall* | 0.000853 | 0.001549 | 0.101658 | 1.880233 | 0.550558 | 0.581936 |
| *Fruits* | -0.17807 | 0.164504 | -2.067797 | -0.553040 | -1.08245 | 0.279054 |
| *Insects* | -0.10335 | 0.161374 | -0.889191 | 0.237711 | -0.64042 | 0.521900 |
| *Zero Inflation model* | | | | | | |
| *(Intercept)* | -7.30201 | 5.612428 | -8.17909 | 0.748054 | -1.30104 | - |
| *Temperature* | -0.42346 | 0.398727 | -2.49049 | -0.20419 | -1.06204 | 0.288218 |
| *Humidity* | 0.228497 | 0.166333 | -0.26164 | 0.028318 | 1.373735 | 0.169524 |
