# Supplementary material for: Small vertebrate consumption by capuchin monkeys (Sapajus libidinosus): Insights into the human predatory pattern
Source: PLoS One. 2026 Aug 26;21(8):e0355204. doi: 10.1371/journal.pone.0355204 (PMC13514029; doi:10.1371/journal.pone.0355204)
Supplement: Supplementary file 9 [file pone.0355204.s014.docx]

**Reviewer #1**

1. **Hypothesis #6, Necessity:**

I believe that this hypothesis is more linked to the absence or reduction of the fruit and invertebrate availability than to environmental factors. We need to think (ecologically) that such variables can indirectly affect the fruit and invertebrate availabilities. So, the better fashion - I think - to test or think is associating with the food resources. The variable influencing diet and behaviour is food items and not rainfall, temperature, and humidity. These can or not influence the appearance of fruit and invertebrates in a given site. Furthermore, to test this hypothesis, perhaps it is better to relate vertebrate consumption with fruit consumption. Thus, the authors could demonstrate the necessity to exploit other food resources.

**Response:** Thank you for your feedback. We have edited the framing of this hypothesis so that it does not address necessity, but rather simply tests for environmental effects over predation rates in a way suggested by Reviewer #2. We hope the new framing of our hypothesis can better address this concern.

2. **Environmental variables:**

The manuscript works with three variables – rainfall, temperature, and humidity – to potentially explain the variation in vertebrate consumption by capuchins. However, two of these variables (rainfall and humidity) will have an effect on fruit and insect availability. So, I do not believe that both can contribute to the understanding of the findings. Thus, to test

**Response:**

Correlations among predictor variables were assessed using Spearman’s rank correlation coefficient and variance inflation factors. Spearman’s ρ indicated only low to moderate pairwise associations, and all VIF values were below 1.25, suggesting negligible multicollinearity. The strongest association was a moderate correlation between insect availability and rainfall (ρ=0.45). However, low VIF values indicated that the model was still able to distinguish between the seasonal increase in insect availability and the direct effect of rainfall on capuchin predation activity. In addition, model comparison based on AIC values showed that models including both environmental variables (rainfall, temperature, and humidity) and food availability variables (fruit and insect availability) consistently outperformed models including environmental variables alone. Because our a priori model selection procedure was based on comparing biologically motivated candidate models and retaining the model with the strongest AIC support, both the environmental variables and the fruit and insect availability variables were therefore kept in the final model. We have added an explicit clarification of this procedure in the methods (subsection Data analysis: statistical modelling) and a supplementary table with VIF values (S1 Table).

3. **Energy shortfall hypothesis:**

I believe that a correlation between vertebrate consumption vs. fruit consumption could help us to understand this hypothesis. The fruit availability is a great measure. But, it does not demonstrate whether the fruit consumption is (positively or negatively) linked to vertebrate intake. However, it needs to know if fruit consumption is associated with vertebrate consumption.

**Response:** Thank you for your feedback. While we appreciate that fruit consumption would have been a very interesting covariate to include in our model, we are not able to collect the necessary data retrospectively. However, we included and expanded on this limitation in our Discussion.

4. **Humidity:**

I would like to see a discussion related to the influence of humidity on the probability of an excess zero in the predation events. If such a variable was measured and incorporated in the study (i.e., data analysis) is necessary to discuss it.

**Response:** Thank you for your feedback. We have now included a discussion of the influence of humidity on this variable in our Discussion section.

5. **Figures:**

I do not know the reason, but the figures are not included in the manuscript to review. So, it was not possible to evaluate some scenarios inserted in the text.

**Response:** We have now included all relevant figures.

**Reviewer #2**

1. **Introduction:**

The introduction to this manuscript would benefit from major revisions to tighten it up. While relevant literature is cited, the current structure reads more like an information dump instead of a focused funnel toward the hypotheses. Redundant and overly dense sentences obscure the core theoretically background and the importance of the hypotheses the authors propose to text. Additionally, several references appear to be incorrectly formatted starting in the Into and persisting throughout the manuscript. For instance, line 169 reads "When reviewed by (1),…" where an author name should appear. The paper as whole would benefit from a careful review for these in-text citations.

**Response:** Thank you for your feedback. The introduction was majorly revised and edited according to these comments, better structured as the “focused funnel toward the hypotheses” the reviewer suggested. We have also reviewed all in-text citations and edited incorrect formatting.

2. **Hypotheses:**

The authors present a thoughtful hypothesis framework to address the drivers of vertebrate predation in these groups of capuchins. Testing predation rates between provision and non provisioned groups is well conceived. The protein in availability hypothesis is reasonable since predation might be driven by the need for macro and micronutrient needs.

Hypothesis 1 and hypothesis 2.1 appeared to be contradictory as written. Hypothesis one predicts that males will have higher vertebrate predation rates because of their increased body mass. Whereas hypothesis 2.1 predicts that females will have higher vertebrate predation rates than adult males because of reproduction and lactation needs. Would a better test of hypothesis 2.1 be that pregnant or lactating females would have increased vertebrate predation rates than non-pregnant non lactating females? Or, and it’s not clear if this possible from that dataset, since you know individuals in the dataset could you compare individuals of similar body mass?

Hypotheses 5 and 6 should be presented as a null and alternative. As written these two hypotheses are testing if predation rates are influenced by environmental factors. The authors are then interpreting whether it's opportunistic or necessity. In my opinion this would better be presented as a null with an alternative that environment impacts predation rates and save the discussion of opportunity and necessity in the context of different prey types for the discussion.

The last hypothesis concerns food transfer and hunting and wild capuchins. This hypothesis is presented differently than the previous hypotheses, with the hypothesis buried within the text of the paragraph. I wonder if the authors could weave in the discussion of hunting and food transfer among primates in general and capuchin specifically somewhere in the main part of the introduction prior to introducing their hypotheses.

**Response:**

Thank you for your feedback. Regarding hypotheses 1 and 2.1 (now presented as 1.1 and 1.2 in the revised manuscript), testing whether pregnant or lactating females have increased vertebrate predation rates than non-pregnant or non-lactating females is not possible from the dataset. However, we have now presented the two hypotheses as alternatives concerning sex biases on predation rates: either males will have higher predation rates, or females will (or neither of the two). We hope this revised format can clarify the need for alternative hypotheses regarding sex biases in predation rates.

Regarding hypotheses 5 and 6 (now presented as 5.1 and 5.2), we have re-framed these as null and alternative as suggested by the reviewer.

Regarding the hypothesis on food transfer and hunting: we have included this hypothesis with the others and added the discussion on hunting and food transfer in the main part of the introduction prior to the hypotheses, as suggested by the reviewer.

3. **Methods:**

The statistical modeling approach is used here appears appropriate and well justified. Maybe I missed this, but why is rainfall in the conditional model but not in the zero inflation model? Was that an a priori decision or was it from AIC comparisons? I assume, perhaps incorrectly, that humidity and rainfall or rainfall and fruit/insect availability covary somewhat. Did the authors test for correlations among predictors? Could this be addressed with a sentence or two in the methods?

**Response:**

Rainfall is in the conditional model but not in the zero inflation model because this model structure improved BIC and AIC substantially (and it also helped a lot with the overall modelling architecture, as models tended to not be able to converge in the opposite solution).

Correlations among predictor variables were assessed using Spearman’s rank correlation coefficient and variance inflation factors. Spearman’s ρ indicated only low to moderate pairwise associations, and all VIF values were below 1.25, suggesting negligible multicollinearity. The strongest association was a moderate correlation between insect availability and rainfall (ρ=0.45). However, low VIF values indicated that the model was still able to distinguish between the seasonal increase in insect availability and the direct effect of rainfall on capuchin predation activity. In addition, model comparison based on AIC values showed that models including both environmental variables (rainfall, temperature, and humidity) and food availability variables (fruit and insect availability) consistently outperformed models including environmental variables alone. Because our a priori model selection procedure was based on comparing biologically motivated candidate models and retaining the model with the strongest AIC support, both the environmental variables and the fruit and insect availability variables were therefore kept in the final model. We have addressed this explicitly in the methods (subsection Data analysis: statistical modelling) and added a supplementary table with VIF values (S1 Table).

4. **Results:**

With the caveat that I can’t evaluate the results completely without the figures, the results are clearly reported.

**Response:** We have now included all relevant figures.

5. **Discussion:**

I have no major concerns about the discussion. A minor suggestion is to integrate Table 5 into the results.

**Response:** Thank you for your feedback. However, we believe Table 5 is better suited for a visual summary of results in the Discussion section than for the Results. The Results already thoroughly detail the information in Table 5, so including it in this section would be repetitive. In contrast, the presence of Table 5 in the Discussion makes the dissection of the main results of this paper easier for the reader, as there are many hypotheses addressed in this article.

**Academic editor**

**1. Please ensure that your manuscript meets PLOS ONE's style requirements, including those for file naming. The PLOS ONE style templates can be found at https://journals.plos.org/plosone/s/file?id=wjVg/PLOSOne_formatting_sample_main_body.pdf and https://journals.plos.org/plosone/s/file?id=ba62/PLOSOne_formatting_sample_title_authors_affiliations.pdf.**

**Response:** All style templates have been checked and applied. Image files have been re-formatted as per instructions.

**2. Please include a complete copy of PLOS’ questionnaire on inclusivity in global research in your revised manuscript. Our policy for research in this area aims to improve transparency in the reporting of research performed outside of researchers’ own country or community. The policy applies to researchers who have travelled to a different country to conduct research, research with Indigenous populations or their lands, and research on cultural artefacts. The questionnaire can also be requested at the journal’s discretion for any other submissions, even if these conditions are not met. Please find more information on the policy and a link to download a blank copy of the questionnaire here: https://journals.plos.org/plosone/s/best-practices-in-research-reporting . Please upload a completed version of your questionnaire as Supporting Information when you resubmit your manuscript.**

**Response:** The questionnaire has been completed and is uploaded as Checklist_S1.pdf. We reference this checklist in our updated Methods section, subsection “Inclusivity in global research”.

**3. In your Methods section, please provide additional information regarding the permits you obtained for the work. Please ensure you have included the full name of the authority that approved the field site access and, if no permits were required, a brief statement explaining why.**

**Response:** Our “Ethical note” section has been updated to provide additional information on the permits obtained for the work and the authority that approved field access.

**4. Thank you for stating in your Funding Statement:**

**“This work was supported by University La Sapienza of Rome (NS), EU FP6 NEST Programme ANALOGY (NS: No 029088), FAPESP (MPV: 06/51578-9, OMF: 08/52293-3 and PI: 06/51577-2 and 08/55684-3), CNPq; CAPES (NS: 017/2012), ISTC-CNR, Ethoikos srl. The funders had no role in study design, data collection and analysis, decision to publish, or preparation of the manuscript.”.**

**Please provide an amended statement that declares *all* the funding or sources of support (whether external or internal to your organization) received during this study, as detailed online in our guide for authors at http://journals.plos.org/plosone/s/submit-now. Please also include the statement “There was no additional external funding received for this study.” in your updated Funding Statement.**

**Please include your amended Funding Statement within your cover letter. We will change the online submission form on your behalf.**

**Response**: We have included the amended Funding Statement in our cover letter (Cover Letter Plos One with Amended Funding Statement.docx).

**5. Please upload a copy of Figure 1-6, to which you refer in your text. If the figure is no longer to be included as part of the submission please remove all reference to it within the text.**

**Response:** We have now uploaded copies of Figures 1-6 as .tiff files and in correct formatting.

**6. If the reviewer comments include a recommendation to cite specific previously published works, please review and evaluate these publications to determine whether they are relevant and should be cited. There is no requirement to cite these works unless the editor has indicated otherwise.**

**Response:** All citation recommendations by reviewers were evaluated as relevant and were included as references in the updated manuscript.
