# Supplementary material for: Small vertebrate consumption by capuchin monkeys (Sapajus libidinosus): Insights into the human predatory pattern
Source: PLoS One. 2026 Aug 26;21(8):e0355204. doi: 10.1371/journal.pone.0355204 (PMC13514029; doi:10.1371/journal.pone.0355204)
Supplement: Supplementary file 10 [file pone.0355204.s017.docx]

**Reviewer #1**

Dear Dr. Carvalho and coauthors,

The manuscript was substantially improved, and all comments/suggestions/cavets were incorporated in this revised version. The text is well-written, the hypotheses are clear, and the methods and data analysis are very well described. The graphics and table are excellent (unfortunately). I don't know why they are not in the previous submission. The discussion is clear and direct, and the authors brought up issues related to the potential absence of data. Excellent job.

I made a few comments/suggestions in the manuscript - see attached - but I could recommend that all those suggestions that were included at the end of each paragraph in the discussion can be moved to the last topic. The text is ok, but in almost all paragraphs, some suggestions for new studies were proposed. So, please, and if possible, move it to the last topic (after discussion). I can be wrong, but I believe that is the better place.

Congratulations.

**Response**: Thank you very much for your kind comments, your feedback has been very helpful and has greatly improved our manuscript. We have moved most suggestions for future research in the discussion to the section “Future directions”, as requested. We have only left one minor suggestion for future work in the discussion (line 680) because we believe it fits better in the context of the paragraph it is in, rather than as a standalone suggestion.

We address each of your specific comments below.

General comments:

Firstly, congratulations to the authors on this work, which clearly involved extensive fieldwork. Regarding the manuscript text, I agree with the previous Reviewer 2 that the manuscript would benefit from a thorough general review to improve clarity and, specifically, to address overly long sentences. For example, lines 170 to 175 consist of a single six-line sentence. Please break down such long sentences into shorter phrases. As a rule of thumb, try to keep a "one sentence,

one idea" approach throughout the text.

**Response:** thank you very much for your congratulations and your feedback. We have gone through the text and reviewed it for clarity, with particular care to address long sentences. We hope this improves readability.

Specific comments:

Query 1: Please ensure that the header row is repeated on every page for Table 1.

**Response:** Thank you for your comment; we have now implemented this.

Query 2: Line 152: Please specify the country where “Fazenda Boa Vista” is located. The same applies to “Santa Rosa” on line 164.

**Response:** Thank you for your comment; we have now added this.

Query 3: Line 194: The font size and color of the numbers are inconsistent with the rest of the text. Similarly, lines 571, 685, and 686 contain words with formatting inconsistencies (different font sizes/colors). Please standardize the typography.

**Response:** Thank you for pointing this out, we have now made sure the typography is consistent throughout the document.

Query 4: In Hypotheses 4.1 and 4.2, it is stated that vertebrate predation rates increase during periods of fruit and insect scarcity because vertebrates replace these other high-protein food items. Does this imply that fruits and insects are preferred over vertebrates? Considering that vertebrates generally offer higher protein content than insects and fruits, wouldn't the predators naturally prefer vertebrates and only rely on other items when vertebrates are harder to find?

Please clarify this ecological rationale.

**Response**: Thank you very much for your comment. These hypotheses point towards the following ecological rationale: hunting vertebrates likely requires greater energy expenditure and results in unpredictable returns compared to foraging for insects and fruits. Therefore, we hypothesize that fruits and insects are generally preferred for their higher net energy yield per unit of foraging time - except, potentially, in periods where their availability is low enough that the nutritional yield of hunting vertebrates becomes “worthwhile” (Fedigan, 1990). We have added an explanation of this ecological rationale in our introduction ahead of our hypotheses (lines 183-188), which we hope better clarifies this for readers.

Query 5: Please insert the equation immediately after it is first mentioned in the text.

**Response:** Thank you for pointing this out, we have now made sure the equation is inserted directly after it is first mentioned in the text.

Query 6: Table 5: I agree with the previous Reviewer 2 that Table 5 belongs in the Results section rather than the Discussion. While I understand the authors' intent to summarize findings within the Discussion, this can be achieved via a concise textual summary. Presenting raw data in a table format is unsuited for the Discussion section, as it shifts the focus back to data description rather than critical discussion.

**Response:** please see below.

Query 7 : In the Discussion section, I missed a structured and explicit discussion regarding the initial hypotheses. Please clearly state which hypotheses were corroborated or refuted and directly connect these outcomes with the existing literature.

**Response for Queries 6 and 7:** Thank you very much for your comments. In light of your feedback, we have decided to eliminate Table 5 and instead integrate the discussion regarding the initial hypotheses into the text in the “Discussion” section. We explicitly address whether the results support or not our initial hypotheses in writing, rather than in a table format, and connect these outcomes with the literature. We believe this is a good compromise, as we did not believe that a table explicitly stating whether hypotheses were corroborated or refuted would have fit well in the Results section either. We hope this addresses both your queries.

**Responses to comments left directly on the manuscript**

Thank you very much for your comments, we have gone through them and made all the changes suggested. The only exceptions are the following: you commented on Figures 4 and 5 to “Please, insert the dots (samples) in the graphic to facilitate the verification of the dispersion.”

We tried adding the points this way, but we felt that it made the main pattern much harder to discern and the figures more difficult to interpret, especially for fruit availability. The regression curve is based on the scaled predictor (0–1), whereas the raw observations span a much wider range (0–6), so the points become somewhat misleading visually and they compress what we really want to know/see (i.e., the curve). Please see below:

One possible solution would be to display the points more vertically (e.g., with some jitter), but we still think the result looks considerably worse than the original figures:

Since the response variable consists of discrete values (0–6 predation events) rather than a continuous distribution, we believe that overlaying the raw points on the regression curve may give a less clear impression of the relationship. Therefore, although we understand the value of showing the dispersion and we very much appreciate the reviewer's comments, we are not sure this is the best representation for this data.

**Reviewer #2**

**4. Have the authors made all data underlying the findings in their manuscript fully available?**

**Reviewer #3: No**

Thank you for your feedback. You can find the data underlying the findings in S2 File: Study Data.
