# Supplementary material for: Small vertebrate consumption by capuchin monkeys (Sapajus libidinosus): Insights into the human predatory pattern
Source: PLoS One. 2026 Aug 26;21(8):e0355204. doi: 10.1371/journal.pone.0355204 (PMC13514029; doi:10.1371/journal.pone.0355204)
Supplement: Peer review history

# Peer review: Decision Letter 0

**DOI:** 10.1371/journal.pone.0355204.r001

**Authors:** Brittany N. Florkiewicz

23 Feb 2026

PONE-D-25-66742

Small vertebrate consumption by capuchin monkeys (Sapajus libidinosus): Insights into the human predatory pattern

PLOS One

Dear Dr. Carvalho,

Thank you for submitting your manuscript to PLOS One. I have received feedback from two expert reviewers, both of whom have requested major revisions to several sections of the manuscript. I encourage you to address their requests and ensure that all your submission materials are complete and available for review.

Please submit your revised manuscript by Apr 09 2026 11:59PM. If you will need more time than this to complete your revisions, please reply to this message or contact the journal office at plosone@plos.org. When you're ready to submit your revision, log on to https://www.editorialmanager.com/pone/ and select the 'Submissions Needing Revision' folder to locate your manuscript file.

Please include the following items when submitting your revised manuscript:

- A letter that responds to each point raised by the academic editor and reviewer(s). You should upload this letter as a separate file labeled 'Response to Reviewers'.
- A marked-up copy of your manuscript that highlights changes made to the original version. You should upload this as a separate file labeled 'Revised Manuscript with Track Changes'.
- An unmarked version of your revised paper without tracked changes. You should upload this as a separate file labeled 'Manuscript'.

If you would like to make changes to your financial disclosure, please include your updated statement in your cover letter. Guidelines for resubmitting your figure files are available below the reviewer comments at the end of this letter.

If applicable, we recommend that you deposit your laboratory protocols in protocols.io to enhance the reproducibility of your results. Protocols.io assigns your protocol its own identifier (DOI) so that it can be cited independently in the future. For instructions see: https://journals.plos.org/plosone/s/submission-guidelines#loc-laboratory-protocols. Additionally, PLOS ONE offers an option for publishing peer-reviewed Lab Protocol articles, which describe protocols hosted on protocols.io. Read more information on sharing protocols at https://plos.org/protocols?utm_medium=editorial-email&utm_source=authorletters&utm_campaign=protocols.

We look forward to receiving your revised manuscript.

Kind regards,

Brittany N. Florkiewicz, Ph.D.

Academic Editor

PLOS One

Journal Requirements:

When submitting your revision, we need you to address these additional requirements.

1. Please ensure that your manuscript meets PLOS ONE's style requirements, including those for file naming. The PLOS ONE style templates can be found at

https://journals.plos.org/plosone/s/file?id=wjVg/PLOSOne_formatting_sample_main_body.pdf and

https://journals.plos.org/plosone/s/file?id=ba62/PLOSOne_formatting_sample_title_authors_affiliations.pdf.

2.  Please include a complete copy of PLOS’ questionnaire on inclusivity in global research in your revised manuscript. Our policy for research in this area aims to improve transparency in the reporting of research performed outside of researchers’ own country or community. The policy applies to researchers who have travelled to a different country to conduct research, research with Indigenous populations or their lands, and research on cultural artefacts. The questionnaire can also be requested at the journal’s discretion for any other submissions, even if these conditions are not met.  Please find more information on the policy and a link to download a blank copy of the questionnaire here: https://journals.plos.org/plosone/s/best-practices-in-research-reporting. Please upload a completed version of your questionnaire as Supporting Information when you resubmit your manuscript.

3. In your Methods section, please provide additional information regarding the permits you obtained for the work. Please ensure you have included the full name of the authority that approved the field site access and, if no permits were required, a brief statement explaining why.

4. Thank you for stating in your Funding Statement:

“This work was supported by University La Sapienza of Rome (NS), EU FP6 NEST Programme ANALOGY (NS: No 029088), FAPESP (MPV: 06/51578-9, OMF: 08/52293-3 and PI: 06/51577-2 and 08/55684-3), CNPq; CAPES (NS: 017/2012), ISTC-CNR, Ethoikos srl. The funders had no role in study design, data collection and analysis, decision to publish, or preparation of the manuscript.”.

Please provide an amended statement that declares *all* the funding or sources of support (whether external or internal to your organization) received during this study, as detailed online in our guide for authors at http://journals.plos.org/plosone/s/submit-now.  Please also include the statement “There was no additional external funding received for this study.” in your updated Funding Statement.

Please include your amended Funding Statement within your cover letter. We will change the online submission form on your behalf.

5. Please upload a copy of Figure 1-6, to which you refer in your text. If the figure is no longer to be included as part of the submission please remove all reference to it within the text.

6. If the reviewer comments include a recommendation to cite specific previously published works, please review and evaluate these publications to determine whether they are relevant and should be cited. There is no requirement to cite these works unless the editor has indicated otherwise.

[Note: HTML markup is below. Please do not edit.]

Reviewers' comments:

Reviewer's Responses to Questions

**Comments to the Author**

1. Is the manuscript technically sound, and do the data support the conclusions?

The manuscript must describe a technically sound piece of scientific research with data that supports the conclusions. Experiments must have been conducted rigorously, with appropriate controls, replication, and sample sizes. The conclusions must be drawn appropriately based on the data presented.

Reviewer #1: Yes

Reviewer #2: Partly

**********

2. Has the statistical analysis been performed appropriately and rigorously?

Reviewer #1: Yes

Reviewer #2: Yes

**********

3. Have the authors made all data underlying the findings in their manuscript fully available?

The PLOS Data policy requires authors to make all data underlying the findings described in their manuscript fully available without restriction, with rare exception (please refer to the Data Availability Statement in the manuscript PDF file). The data should be provided as part of the manuscript or its supporting information, or deposited to a public repository. For example, in addition to summary statistics, the data points behind means, medians and variance measures should be available. If there are restrictions on publicly sharing data—e.g. participant privacy or use of data from a third party—those must be specified.

Reviewer #1: Yes

Reviewer #2: Yes

**********

4. Is the manuscript presented in an intelligible fashion and written in standard English?

PLOS ONE does not copyedit accepted manuscripts, so the language in submitted articles must be clear, correct, and unambiguous. Any typographical or grammatical errors should be corrected at revision, so please note any specific errors here.

Reviewer #1: Yes

Reviewer #2: No

**********

5. Review Comments to the Author

Please use the space provided to explain your answers to the questions above. You may also include additional comments for the author, including concerns about dual publication, research ethics, or publication ethics. (Please upload your review as an attachment if it exceeds 20,000 characters)

Reviewer #1: Manuscript PONE-D-25-66742

Title: Small vertebrate consumption by capuchin monkeys (Sapajus libidinosus): Insights into the human predatory pattern

Authors: Carvalho, S. and collaborators

I am glad to read and revise this well-written manuscript, which presents a robust and reliable database on vertebrate consumption by bearded capuchin monkeys in an ecotonal site in Brazil. Dozens of studies were previously published on this population and study site. Understanding and correlating vertebrate consumption is important due to its contribution to the diet and features linked to behaviour. But here, it is treated or associated with the diet aspects and human predatory patterns.

I made some minor comments on the attached document, and here I would like to bring up a few major concerns in order to clarify the manuscript for the readers.

Hypothesis #6, Necessity:

I believe that this hypothesis is more linked to the absence or reduction of the fruit and invertebrate availability than to environmental factors. We need to think (ecologically) that such variables can indirectly affect the fruit and invertebrate availabilities. So, the better fashion - I think - to test or think is associating with the food resources. The variable influencing diet and behaviour is food items and not rainfall, temperature, and humidity. These can or not influence the appearance of fruit and invertebrates in a given site. Furthermore, to test this hypothesis, perhaps it is better to relate vertebrate consumption with fruit consumption. Thus, the authors could demonstrate the necessity to exploit other food resources.

Environmental variables

The manuscript works with three variables – rainfall, temperature, and humidity – to potentially explain the variation in vertebrate consumption by capuchins. However, two of these variables (rainfall and humidity) will have an effect on fruit and insect availability. So, I do not believe that both can contribute to the understanding of the findings. Thus, to test

Energy shortfall hypothesis

I believe that a correlation between vertebrate consumption vs. fruit consumption could help us to understand this hypothesis. The fruit availability is a great measure. But, it does not demonstrate whether the fruit consumption is (positively or negatively) linked to vertebrate intake. However, it needs to know if fruit consumption is associated with vertebrate consumption.

Humidity

I would like to see a discussion related to the influence of humidity on the probability of an excess zero in the predation events. If such a variable was measured and incorporated in the study (i.e., data analysis) is necessary to discuss it.

Figures

I do not know the reason, but the figures are not included in the manuscript to review. So, it was not possible to evaluate some scenarios inserted in the text.

Reviewer #2: This submission is an analysis of vertebrate predation data from two groups of wild bearded capuchins at a single field site in Brazil. Data were collected periodically over the course of 5 years. The authors aim to test hypotheses concerning the effect of environment, energy needs, and individual skill on vertebrate predation. The authors found that sex, fruit availability and provisioning have a notable effect on predation rates. They highlight the need for study of small vertebrate predation and subsequent faunal assemblages to inform our understanding of the evolution of hominin hunting behavior.

I’ll note here that I did not see the six figures mentioned in the results either within the manuscript text (doc or pdf) or as separate files for downloading. Tables and supplementary info were viewable. This omission precluded a complete review of the results and discussion portions of the manuscript. However, I was leaning major revision prior to reading the results, so it did not ultimately impact my accept/revise/reject decision. I recommend figures be included in any revised submission. My major concerns are included below.

Introduction:

The introduction to this manuscript would benefit from major revisions to tighten it up. While relevant literature is cited, the current structure reads more like an information dump instead of a focused funnel toward the hypotheses. Redundant and overly dense sentences obscure the core theoretically background and the importance of the hypotheses the authors propose to text. Additionally, several references appear to be incorrectly formatted starting in the Into and persisting throughout the manuscript. For instance, line 169 reads "When reviewed by (1),…" where an author name should appear. The paper as whole would benefit from a careful review for these in-text citations.

Hypotheses:

The authors present a thoughtful hypothesis framework to address the drivers of vertebrate predation in these groups of capuchins. Testing predation rates between provision and non provisioned groups is well conceived. The protein in availability hypothesis is reasonable since predation might be driven by the need for macro and micronutrient needs.

Hypothesis 1 and hypothesis 2.1 appeared to be contradictory as written. Hypothesis one predicts that males will have higher vertebrate predation rates because of their increased body mass. Whereas hypothesis 2.1 predicts that females will have higher vertebrate predation rates than adult males because of reproduction and lactation needs. Would a better test of hypothesis 2.1 be that pregnant or lactating females would have increased vertebrate predation rates than non-pregnant non lactating females? Or, and it’s not clear if this possible from that dataset, since you know individuals in the dataset could you compare individuals of similar body mass?

Hypotheses 5 and 6 should be presented as a null and alternative. As written these two hypotheses are testing if predation rates are influenced by environmental factors. The authors are then interpreting whether it's opportunistic or necessity. In my opinion this would better be presented as a null with an alternative that environment impacts predation rates and save the discussion of opportunity and necessity in the context of different prey types for the discussion.

The last hypothesis concerns food transfer and hunting and wild capuchins. This hypothesis is presented differently than the previous hypotheses, with the hypothesis buried within the text of the paragraph. I wonder if the authors could weave in the discussion of hunting and food transfer among primates in general and capuchin specifically somewhere in the main part of the introduction prior to introducing their hypotheses.

Methods:

The statistical modeling approach is used here appears appropriate and well justified. Maybe I missed this, but why is rainfall in the conditional model but not in the zero inflation model? Was that an a priori decision or was it from AIC comparisons? I assume, perhaps incorrectly, that humidity and rainfall or rainfall and fruit/insect availability covary somewhat. Did the authors test for correlations among predictors? Could this be addressed with a sentence or two in the methods?

Results:

With the caveat that I can’t evaluate the results completely without the figures, the results are clearly reported.

Discussion:

I have no major concerns about the discussion. A minor suggestion is to integrate Table 5 into the results.

**********

6. PLOS authors have the option to publish the peer review history of their article (what does this mean?). If published, this will include your full peer review and any attached files.

If you choose “no”, your identity will remain anonymous but your review may still be made public.

**Do you want your identity to be public for this peer review?**  For information about this choice, including consent withdrawal, please see our Privacy Policy.

Reviewer #1: No

Reviewer #2: No

**********

[NOTE: If reviewer comments were submitted as an attachment file, they will be attached to this email and accessible via the submission site. Please log into your account, locate the manuscript record, and check for the action link "View Attachments". If this link does not appear, there are no attachment files.]

To ensure your figures meet our technical requirements, please review our figure guidelines: https://journals.plos.org/plosone/s/figures

You may also use PLOS’s free figure tool, NAAS, to help you prepare publication quality figures: https://journals.plos.org/plosone/s/figures#loc-tools-for-figure-preparation.

NAAS will assess whether your figures meet our technical requirements by comparing each figure against our figure specifications.

Attachment Submitted filename: PONE-D-25-66742_Revised.pdf

# Author comment: Author response to Decision Letter 1

**DOI:** 10.1371/journal.pone.0355204.r002

15 Apr 2026

Reviewer #1

1. Hypothesis #6, Necessity:

I believe that this hypothesis is more linked to the absence or reduction of the fruit and invertebrate availability than to environmental factors. We need to think (ecologically) that such variables can indirectly affect the fruit and invertebrate availabilities. So, the better fashion - I think - to test or think is associating with the food resources. The variable influencing diet and behaviour is food items and not rainfall, temperature, and humidity. These can or not influence the appearance of fruit and invertebrates in a given site. Furthermore, to test this hypothesis, perhaps it is better to relate vertebrate consumption with fruit consumption. Thus, the authors could demonstrate the necessity to exploit other food resources.

Response: Thank you for your feedback. We have edited the framing of this hypothesis so that it does not address necessity, but rather simply tests for environmental effects over predation rates in a way suggested by Reviewer #2. We hope the new framing of our hypothesis can better address this concern.

2. Environmental variables:

The manuscript works with three variables – rainfall, temperature, and humidity – to potentially explain the variation in vertebrate consumption by capuchins. However, two of these variables (rainfall and humidity) will have an effect on fruit and insect availability. So, I do not believe that both can contribute to the understanding of the findings. Thus, to test

Response:

3. Correlations among predictor variables were assessed using Spearman’s rank correlation coefficient and variance inflation factors. Spearman’s ρ indicated only low to moderate pairwise associations, and all VIF values were below 1.25, suggesting negligible multicollinearity. The strongest association was a moderate correlation between insect availability and rainfall (ρ=0.45). However, low VIF values indicated that the model was still able to distinguish between the seasonal increase in insect availability and the direct effect of rainfall on capuchin predation activity. In addition, model comparison based on AIC values showed that models including both environmental variables (rainfall, temperature, and humidity) and food availability variables (fruit and insect availability) consistently outperformed models including environmental variables alone. Because our a priori model selection procedure was based on comparing biologically motivated candidate models and retaining the model with the strongest AIC support, both the environmental variables and the fruit and insect availability variables were therefore kept in the final model. We have added an explicit clarification of this procedure in the methods (subsection Data analysis: statistical modelling) and a supplementary table with VIF values (S1 Table).

4. Energy shortfall hypothesis:

I believe that a correlation between vertebrate consumption vs. fruit consumption could help us to understand this hypothesis. The fruit availability is a great measure. But, it does not demonstrate whether the fruit consumption is (positively or negatively) linked to vertebrate intake. However, it needs to know if fruit consumption is associated with vertebrate consumption.

Response: Thank you for your feedback. While we appreciate that fruit consumption would have been a very interesting covariate to include in our model, we are not able to collect the necessary data retrospectively. However, we included and expanded on this limitation in our Discussion.

5. Humidity:

I would like to see a discussion related to the influence of humidity on the probability of an excess zero in the predation events. If such a variable was measured and incorporated in the study (i.e., data analysis) is necessary to discuss it.

Response: Thank you for your feedback. We have now included a discussion of the influence of humidity on this variable in our Discussion section.

6. Figures:

I do not know the reason, but the figures are not included in the manuscript to review. So, it was not possible to evaluate some scenarios inserted in the text.

Response: We have now included all relevant figures.

Reviewer #2

1. Introduction:

The introduction to this manuscript would benefit from major revisions to tighten it up. While relevant literature is cited, the current structure reads more like an information dump instead of a focused funnel toward the hypotheses. Redundant and overly dense sentences obscure the core theoretically background and the importance of the hypotheses the authors propose to text. Additionally, several references appear to be incorrectly formatted starting in the Into and persisting throughout the manuscript. For instance, line 169 reads "When reviewed by (1),…" where an author name should appear. The paper as whole would benefit from a careful review for these in-text citations.

Response: Thank you for your feedback. The introduction was majorly revised and edited according to these comments, better structured as the “focused funnel toward the hypotheses” the reviewer suggested. We have also reviewed all in-text citations and edited incorrect formatting.

2. Hypotheses:

The authors present a thoughtful hypothesis framework to address the drivers of vertebrate predation in these groups of capuchins. Testing predation rates between provision and non provisioned groups is well conceived. The protein in availability hypothesis is reasonable since predation might be driven by the need for macro and micronutrient needs.

Hypothesis 1 and hypothesis 2.1 appeared to be contradictory as written. Hypothesis one predicts that males will have higher vertebrate predation rates because of their increased body mass. Whereas hypothesis 2.1 predicts that females will have higher vertebrate predation rates than adult males because of reproduction and lactation needs. Would a better test of hypothesis 2.1 be that pregnant or lactating females would have increased vertebrate predation rates than non-pregnant non lactating females? Or, and it’s not clear if this possible from that dataset, since you know individuals in the dataset could you compare individuals of similar body mass?

Hypotheses 5 and 6 should be presented as a null and alternative. As written these two hypotheses are testing if predation rates are influenced by environmental factors. The authors are then interpreting whether it's opportunistic or necessity. In my opinion this would better be presented as a null with an alternative that environment impacts predation rates and save the discussion of opportunity and necessity in the context of different prey types for the discussion.

The last hypothesis concerns food transfer and hunting and wild capuchins. This hypothesis is presented differently than the previous hypotheses, with the hypothesis buried within the text of the paragraph. I wonder if the authors could weave in the discussion of hunting and food transfer among primates in general and capuchin specifically somewhere in the main part of the introduction prior to introducing their hypotheses.

Response:

Thank you for your feedback. Regarding hypotheses 1 and 2.1 (now presented as 1.1 and 1.2 in the revised manuscript), testing whether pregnant or lactating females have increased vertebrate predation rates than non-pregnant or non-lactating females is not possible from the dataset. However, we have now presented the two hypotheses as alternatives concerning sex biases on predation rates: either males will have higher predation rates, or females will (or neither of the two). We hope this revised format can clarify the need for alternative hypotheses regarding sex biases in predation rates.

Regarding hypotheses 5 and 6 (now presented as 5.1 and 5.2), we have re-framed these as null and alternative as suggested by the reviewer.

Regarding the hypothesis on food transfer and hunting: we have included this hypothesis with the others and added the discussion on hunting and food transfer in the main part of the introduction prior to the hypotheses, as suggested by the reviewer.

3. Methods:

The statistical modeling approach is used here appears appropriate and well justified. Maybe I missed this, but why is rainfall in the conditional model but not in the zero inflation model? Was that an a priori decision or was it from AIC comparisons? I assume, perhaps incorrectly, that humidity and rainfall or rainfall and fruit/insect availability covary somewhat. Did the authors test for correlations among predictors? Could this be addressed with a sentence or two in the methods?

Response:

Rainfall is in the conditional model but not in the zero inflation model because this model structure improved BIC and AIC substantially (and it also helped a lot with the overall modelling architecture, as models tended to not be able to converge in the opposite solution).

Correlations among predictor variables were assessed using Spearman’s rank correlation coefficient and variance inflation factors. Spearman’s ρ indicated only low to moderate pairwise associations, and all VIF values were below 1.25, suggesting negligible multicollinearity. The strongest association was a moderate correlation between insect availability and rainfall (ρ=0.45). However, low VIF values indicated that the model was still able to distinguish between the seasonal increase in insect availability and the direct effect of rainfall on capuchin predation activity. In addition, model comparison based on AIC values showed that models including both environmental variables (rainfall, temperature, and humidity) and food availability variables (fruit and insect availability) consistently outperformed models including environmental variables alone. Because our a priori model selection procedure was based on comparing biologically motivated candidate models and retaining the model with the strongest AIC support, both the environmental variables and the fruit and insect availability variables were therefore kept in the final model. We have addressed this explicitly in the methods (subsection Data analysis: statistical modelling) and added a supplementary table with VIF values (S1 Table).

4. Results:

With the caveat that I can’t evaluate the results completely without the figures, the results are clearly reported.

Response: We have now included all relevant figures.

5. Discussion:

I have no major concerns about the discussion. A minor suggestion is to integrate Table 5 into the results.

Response: Thank you for your feedback. However, we believe Table 5 is better suited for a visual summary of results in the Discussion section than for the Results. The Results already thoroughly detail the information in Table 5, so including it in this section would be repetitive. In contrast, the presence of Table 5 in the Discussion makes the dissection of the main results of this paper easier for the reader, as there are many hypotheses addressed in this article.

Academic editor

1. Please ensure that your manuscript meets PLOS ONE's style requirements, including those for file naming. The PLOS ONE style templates can be found at https://journals.plos.org/plosone/s/file?id=wjVg/PLOSOne_formatting_sample_main_body.pdf and https://journals.plos.org/plosone/s/file?id=ba62/PLOSOne_formatting_sample_title_authors_affiliations.pdf.

Response: All style templates have been checked and applied. Image files have been re-formatted as per instructions.

2. Please include a complete copy of PLOS’ questionnaire on inclusivity in global research in your revised manuscript. Our policy for research in this area aims to improve transparency in the reporting of research performed outside of researchers’ own country or community. The policy applies to researchers who have travelled to a different country to conduct research, research with Indigenous populations or their lands, and research on cultural artefacts. The questionnaire can also be requested at the journal’s discretion for any other submissions, even if these conditions are not met. Please find more information on the policy and a link to download a blank copy of the questionnaire here: https://journals.plos.org/plosone/s/best-practices-in-research-reporting . Please upload a completed version of your questionnaire as Supporting Information when you resubmit your manuscript.

Response: The questionnaire has been completed and is uploaded as Checklist_S1.pdf. We reference this checklist in our updated Methods section, subsection “Inclusivity in global research”.

3. In your Methods section, please provide additional information regarding the permits you obtained for the work. Please ensure you have included the full name of the authority that approved the field site access and, if no permits were required, a brief statement explaining why.

Response: Our “Ethical note” section has been updated to provide additional information on the permits obtained for the work and the authority that approved field access.

4. Thank you for stating in your Funding Statement:

“This work was supported by University La Sapienza of Rome (NS), EU FP6 NEST Programme ANALOGY (NS: No 029088), FAPESP (MPV: 06/51578-9, OMF: 08/52293-3 and PI: 06/51577-2 and 08/55684-3), CNPq; CAPES (NS: 017/2012), ISTC-CNR, Ethoikos srl. The funders had no role in study design, data collection and analysis, decision to publish, or preparation of the manuscript.”.

Please provide an amended statement that declares *all* the funding or sources of support (whether external or internal to your organization) received during this study, as detailed online in our guide for authors at http://journals.plos.org/plosone/s/submit-now. Please also include the statement “There was no additional external funding received for this study.” in your updated Funding Statement.

Please include your amended Funding Statement within your cover letter. We will change the online submission form on your behalf.

Response: We have included the amended Funding Statement in our cover letter (Cover Letter Plos One with Amended Funding Statement.docx).

5. Please upload a copy of Figure 1-6, to which you refer in your text. If the figure is no longer to be included as part of the submission please remove all reference to it within the text.

Response: We have now uploaded copies of Figures 1-6 as .tiff files and in correct formatting.

6. If the reviewer comments include a recommendation to cite specific previously published works, please review and evaluate these publications to determine whether they are relevant and should be cited. There is no requirement to cite these works unless the editor has indicated otherwise.

Response: All citation recommendations by reviewers were evaluated as relevant and were included as references in the updated manuscript.

Attachment Submitted filename: Response to Reviewers.docx

# Peer review: Decision Letter 1

**DOI:** 10.1371/journal.pone.0355204.r003

**Authors:** Brittany N. Florkiewicz

2 Jun 2026

PONE-D-25-66742R1

Small vertebrate consumption by capuchin monkeys (Sapajus libidinosus): Insights into the human predatory pattern

PLOS One

Dear Dr. Carvalho,

Thank you for submitting your manuscript to PLOS ONE. After careful consideration, we feel that it has merit but does not fully meet PLOS ONE’s publication criteria as it currently stands. Therefore, we invite you to submit a revised version of the manuscript that addresses the points raised during the review process.

Please submit your revised manuscript by Jul 16 2026 11:59PM. If you will need more time than this to complete your revisions, please reply to this message or contact the journal office at plosone@plos.org. When you're ready to submit your revision, log on to https://www.editorialmanager.com/pone/ and select the 'Submissions Needing Revision' folder to locate your manuscript file.

Please include the following items when submitting your revised manuscript:

- A letter that responds to each point raised by the academic editor and reviewer(s). You should upload this letter as a separate file labeled 'Response to Reviewers'.
- A marked-up copy of your manuscript that highlights changes made to the original version. You should upload this as a separate file labeled 'Revised Manuscript with Track Changes'.
- An unmarked version of your revised paper without tracked changes. You should upload this as a separate file labeled 'Manuscript'.

If you would like to make changes to your financial disclosure, please include your updated statement in your cover letter. Guidelines for resubmitting your figure files are available below the reviewer comments at the end of this letter.

If applicable, we recommend that you deposit your laboratory protocols in protocols.io to enhance the reproducibility of your results. Protocols.io assigns your protocol its own identifier (DOI) so that it can be cited independently in the future. For instructions see: https://journals.plos.org/plosone/s/submission-guidelines#loc-laboratory-protocols. Additionally, PLOS ONE offers an option for publishing peer-reviewed Lab Protocol articles, which describe protocols hosted on protocols.io. Read more information on sharing protocols at https://plos.org/protocols?utm_medium=editorial-email&utm_source=authorletters&utm_campaign=protocols.

As the corresponding author, your ORCID iD is verified in the submission system and will appear in the published article. PLOS supports the use of ORCID, and we encourage all coauthors to register for an ORCID iD and use it as well. Please encourage your coauthors to verify their ORCID iD within the submission system before final acceptance, as unverified ORCID iDs will not appear in the published article. *Only*  the individual author can complete the verification step; PLOS staff *cannot*  verify ORCID iDs on behalf of authors.

We look forward to receiving your revised manuscript.

Kind regards,

Brittany N. Florkiewicz, Ph.D.

Academic Editor

PLOS One

**Journal Requirements:**

1. If the reviewer comments include a recommendation to cite specific previously published works, please review and evaluate these publications to determine whether they are relevant and should be cited. There is no requirement to cite these works unless the editor has indicated otherwise.

2. Please review your reference list to ensure that it is complete and correct. If you have cited papers that have been retracted, please include the rationale for doing so in the manuscript text, or remove these references and replace them with relevant current references. Any changes to the reference list should be mentioned in the rebuttal letter that accompanies your revised manuscript. If you need to cite a retracted article, indicate the article’s retracted status in the References list and also include a citation and full reference for the retraction notice.

**Additional Editor Comments:**

I appreciate your patience, and I sincerely apologize for the delay. We needed to find a new reviewer because one of our previous reviewers was unavailable. Both reviewers, one previous and one new, provided additional minor comments for you to address, which are sent as attachments. Therefore, I would like to request that you address their comments and concerns. Once you have revised the manuscript accordingly, I will review the changes before issuing a final decision on its publication. Thank you for resubmitting your manuscript, and I look forward to reviewing the revised version!

[Note: HTML markup is below. Please do not edit.]

Reviewers' comments:

Reviewer's Responses to Questions

**Comments to the Author**

1. If the authors have adequately addressed your comments raised in a previous round of review and you feel that this manuscript is now acceptable for publication, you may indicate that here to bypass the “Comments to the Author” section, enter your conflict of interest statement in the “Confidential to Editor” section, and submit your "Accept" recommendation.

Reviewer #1: All comments have been addressed

Reviewer #3: (No Response)

**********

2. Is the manuscript technically sound, and do the data support the conclusions?

The manuscript must describe a technically sound piece of scientific research with data that supports the conclusions. Experiments must have been conducted rigorously, with appropriate controls, replication, and sample sizes. The conclusions must be drawn appropriately based on the data presented.

Reviewer #1: Yes

Reviewer #3: Yes

**********

3. Has the statistical analysis been performed appropriately and rigorously?

Reviewer #1: Yes

Reviewer #3: Yes

**********

4. Have the authors made all data underlying the findings in their manuscript fully available?

The PLOS Data policy requires authors to make all data underlying the findings described in their manuscript fully available without restriction, with rare exception (please refer to the Data Availability Statement in the manuscript PDF file). The data should be provided as part of the manuscript or its supporting information, or deposited to a public repository. For example, in addition to summary statistics, the data points behind means, medians and variance measures should be available. If there are restrictions on publicly sharing data—e.g. participant privacy or use of data from a third party—those must be specified.

Reviewer #1: Yes

Reviewer #3: No

**********

5. Is the manuscript presented in an intelligible fashion and written in standard English?

PLOS ONE does not copyedit accepted manuscripts, so the language in submitted articles must be clear, correct, and unambiguous. Any typographical or grammatical errors should be corrected at revision, so please note any specific errors here.

Reviewer #1: Yes

Reviewer #3: Yes

**********

6. Review Comments to the Author

Please use the space provided to explain your answers to the questions above. You may also include additional comments for the author, including concerns about dual publication, research ethics, or publication ethics. (Please upload your review as an attachment if it exceeds 20,000 characters)

**Reviewer #1:**  Dear Dr. Carvalho and coauthors,

The manuscript was substantially improved, and all comments/suggestions/cavets were incorporated in this revised version. The text is well-written, the hypotheses are clear, and the methods and data analysis are very well described. The graphics and table are excellent (unfortunately). I don't know why they are not in the previous submission. The discussion is clear and direct, and the authors brought up issues related to the potential absence of data. Excellent job.

I made a few comments/suggestions in the manuscript - see attached - but I could recommend that all those suggestions that were included at the end of each paragraph in the discussion can be moved to the last topic. The text is ok, but in almost all paragraphs, some suggestions for new studies were proposed. So, please, and if possible, move it to the last topic (after discussion). I can be wrong, but I believe that is the better place.

Congratulations.

**Reviewer #3:**  (No Response)

**********

7. PLOS authors have the option to publish the peer review history of their article (what does this mean?). If published, this will include your full peer review and any attached files.

If you choose “no”, your identity will remain anonymous but your review may still be made public.

**Do you want your identity to be public for this peer review?**  For information about this choice, including consent withdrawal, please see our Privacy Policy.

Reviewer #1: **Yes:**  João Pedro Souza-Alves

Reviewer #3: No

**********

[NOTE: If reviewer comments were submitted as an attachment file, they will be attached to this email and accessible via the submission site. Please log into your account, locate the manuscript record, and check for the action link "View Attachments". If this link does not appear, there are no attachment files.]

To ensure your figures meet our technical requirements, please review our figure guidelines: https://journals.plos.org/plosone/s/figures

You may also use PLOS’s free figure tool, NAAS, to help you prepare publication quality figures: https://journals.plos.org/plosone/s/figures#loc-tools-for-figure-preparation.

NAAS will assess whether your figures meet our technical requirements by comparing each figure against our figure specifications.

Attachment Submitted filename: PONE-D-25-66742_R1_revised.pdf

Attachment Submitted filename: Review_PONE-D-25-66742.pdf

# Author comment: Author response to Decision Letter 2

**DOI:** 10.1371/journal.pone.0355204.r004

16 Jul 2026

Dear reviewers and editor, we thank you kindly for your comments, they very much have helped us improve this manuscript. We have addressed each of the comments directly on the Response to Reviewers files, as well as below. We hope the edits address all your concerns and we greatly appreciate your feedback.

Reviewer #1:

Dear Dr. Carvalho and coauthors,

The manuscript was substantially improved, and all comments/suggestions/cavets were incorporated in this revised version. The text is well-written, the hypotheses are clear, and the methods and data analysis are very well described. The graphics and table are excellent (unfortunately). I don't know why they are not in the previous submission. The discussion is clear and direct, and the authors brought up issues related to the potential absence of data. Excellent job.

I made a few comments/suggestions in the manuscript - see attached - but I could recommend that all those suggestions that were included at the end of each paragraph in the discussion can be moved to the last topic. The text is ok, but in almost all paragraphs, some suggestions for new studies were proposed. So, please, and if possible, move it to the last topic (after discussion). I can be wrong, but I believe that is the better place.

Congratulations.

Response: Thank you very much for your kind comments, your feedback has been very helpful and has greatly improved our manuscript. We have moved most suggestions for future research in the discussion to the section “Future directions”, as requested. We have only left one minor suggestion for future work in the discussion (line 680) because we believe it fits better in the context of the paragraph it is in, rather than as a standalone suggestion.

We address each of your specific comments below.

General comments:

Firstly, congratulations to the authors on this work, which clearly involved extensive fieldwork. Regarding the manuscript text, I agree with the previous Reviewer 2 that the manuscript would benefit from a thorough general review to improve clarity and, specifically, to address overly long sentences. For example, lines 170 to 175 consist of a single six-line sentence. Please break down such long sentences into shorter phrases. As a rule of thumb, try to keep a "one sentence,

one idea" approach throughout the text.

Response: thank you very much for your congratulations and your feedback. We have gone through the text and reviewed it for clarity, with particular care to address long sentences. We hope this improves readability.

Specific comments:

Query 1: Please ensure that the header row is repeated on every page for Table 1.

Response: Thank you for your comment; we have now implemented this.

Query 2: Line 152: Please specify the country where “Fazenda Boa Vista” is located. The same applies to “Santa Rosa” on line 164.

Response: Thank you for your comment; we have now added this.

Query 3: Line 194: The font size and color of the numbers are inconsistent with the rest of the text. Similarly, lines 571, 685, and 686 contain words with formatting inconsistencies (different font sizes/colors). Please standardize the typography.

Response: Thank you for pointing this out, we have now made sure the typography is consistent throughout the document.

Query 4: In Hypotheses 4.1 and 4.2, it is stated that vertebrate predation rates increase during periods of fruit and insect scarcity because vertebrates replace these other high-protein food items. Does this imply that fruits and insects are preferred over vertebrates? Considering that vertebrates generally offer higher protein content than insects and fruits, wouldn't the predators naturally prefer vertebrates and only rely on other items when vertebrates are harder to find?

Please clarify this ecological rationale.

Response: Thank you very much for your comment. These hypotheses point towards the following ecological rationale: hunting vertebrates likely requires greater energy expenditure and results in unpredictable returns compared to foraging for insects and fruits. Therefore, we hypothesize that fruits and insects are generally preferred for their higher net energy yield per unit of foraging time - except, potentially, in periods where their availability is low enough that the nutritional yield of hunting vertebrates becomes “worthwhile” (Fedigan, 1990). We have added an explanation of this ecological rationale in our introduction ahead of our hypotheses (lines 183-188), which we hope better clarifies this for readers.

Query 5: Please insert the equation immediately after it is first mentioned in the text.

Response: Thank you for pointing this out, we have now made sure the equation is inserted directly after it is first mentioned in the text.

Query 6: Table 5: I agree with the previous Reviewer 2 that Table 5 belongs in the Results section rather than the Discussion. While I understand the authors' intent to summarize findings within the Discussion, this can be achieved via a concise textual summary. Presenting raw data in a table format is unsuited for the Discussion section, as it shifts the focus back to data description rather than critical discussion.

Response: please see below.

Query 7 : In the Discussion section, I missed a structured and explicit discussion regarding the initial hypotheses. Please clearly state which hypotheses were corroborated or refuted and directly connect these outcomes with the existing literature.

Response for Queries 6 and 7: Thank you very much for your comments. In light of your feedback, we have decided to eliminate Table 5 and instead integrate the discussion regarding the initial hypotheses into the text in the “Discussion” section. We explicitly address whether the results support or not our initial hypotheses in writing, rather than in a table format, and connect these outcomes with the literature. We believe this is a good compromise, as we did not believe that a table explicitly stating whether hypotheses were corroborated or refuted would have fit well in the Results section either. We hope this addresses both your queries.

Responses to comments left directly on the manuscript:

Thank you very much for your comments, we have gone through them and made all the changes suggested. The only exceptions are the following: you commented on Figures 4 and 5 to “Please, insert the dots (samples) in the graphic to facilitate the verification of the dispersion.”

We tried adding the points this way, but we felt that it made the main pattern much harder to discern and the figures more difficult to interpret, especially for fruit availability. The regression curve is based on the scaled predictor (0–1), whereas the raw observations span a much wider range (0–6), so the points become somewhat misleading visually and they compress what we really want to know/see (i.e., the curve). One possible solution would be to display the points more vertically (e.g., with some jitter), but we still think the result looks considerably worse than the original figures. We have attached the figures for your reference in the Response to Reviewers file.

Since the response variable consists of discrete values (0–6 predation events) rather than a continuous distribution, we believe that overlaying the raw points on the regression curve may give a less clear impression of the relationship. Therefore, although we understand the value of showing the dispersion and we very much appreciate the reviewer's comments, we are not sure this is the best representation for this data.

Reviewer #3:

Have the authors made all data underlying the findings in their manuscript fully available?

Reviewer #3: No

Thank you for your feedback. You can find the data underlying the findings in S2 File: Study Data.

Attachment Submitted filename: Response to Reviewers R2.docx

# Editor report: Decision Letter 2

**DOI:** 10.1371/journal.pone.0355204.r005

**Authors:** Brittany N. Florkiewicz

20 Jul 2026

Small vertebrate consumption by capuchin monkeys (Sapajus libidinosus): Insights into the human predatory pattern

PONE-D-25-66742R2

Dear Dr. Carvalho,

We’re pleased to inform you that your manuscript has been judged scientifically suitable for publication and will be formally accepted for publication once it meets all outstanding technical requirements.

Within one week, you’ll receive an e-mail detailing the required amendments. When these have been addressed, you’ll receive a formal acceptance letter and your manuscript will be scheduled for publication.

An invoice will be generated when your article is formally accepted. Please note, if your institution has a publishing partnership with PLOS and your article meets the relevant criteria, all or part of your publication costs will be covered. Please make sure your user information is up-to-date by logging into Editorial Manager at Editorial Manager® and clicking the ‘Update My Information' link at the top of the page. For questions related to billing, please contact billing support.

If your institution or institutions have a press office, please notify them about your upcoming paper to help maximize its impact. If they’ll be preparing press materials, please inform our press team as soon as possible -- no later than 48 hours after receiving the formal acceptance. Your manuscript will remain under strict press embargo until 2 pm Eastern Time on the date of publication. For more information, please contact onepress@plos.org.

Kind regards,

Brittany N. Florkiewicz, Ph.D.

Academic Editor

PLOS One

Additional Editor Comments (optional):

Thank you for addressing the additional requests from the reviewers. Your manuscript is now suitable for publication, and I believe it will be of great interest to the primate research community. Congratulations!

Reviewers' comments:

# Editor report: Acceptance letter

**DOI:** 10.1371/journal.pone.0355204.r006

**Authors:** Brittany N. Florkiewicz

PONE-D-25-66742R2

PLOS One

Dear Dr. Carvalho,

I'm pleased to inform you that your manuscript has been deemed suitable for publication in PLOS One. Congratulations! Your manuscript is now being handed over to our production team.

At this stage, our production department will prepare your paper for publication. This includes ensuring the following:

* All references, tables, and figures are properly cited

* All relevant supporting information is included in the manuscript submission,

* There are no issues that prevent the paper from being properly typeset

You will receive further instructions from the production team, including instructions on how to review your proof when it is ready. Please keep in mind that we are working through a large volume of accepted articles, so please give us a few days to review your paper and let you know the next and final steps.

Lastly, if your institution or institutions have a press office, please let them know about your upcoming paper now to help maximize its impact. If they'll be preparing press materials, please inform our press team within the next 48 hours. Your manuscript will remain under strict press embargo until 2 pm Eastern Time on the date of publication. For more information, please contact onepress@plos.org.

You will receive an invoice from PLOS for your publication fee after your manuscript has reached the completed accept phase. If you receive an email requesting payment before acceptance or for any other service, this may be a phishing scheme. Learn how to identify phishing emails and protect your accounts at https://explore.plos.org/phishing.

If we can help with anything else, please email us at customercare@plos.org.

Thank you for submitting your work to PLOS ONE and supporting open access.

Kind regards,

PLOS ONE Editorial Office Staff

on behalf of

Dr. Brittany N. Florkiewicz

Academic Editor

PLOS One
